# 4-Azafluorenone and α-Carboline Fluorophores with Green and Violet/Blue Emission

**DOI:** 10.3390/molecules24132378

**Published:** 2019-06-27

**Authors:** Marek Cigáň, Peter Danko, Henrich Brath, Matúš Čakurda, Roman Fišera, Jana Donovalová, Juraj Filo, Martin Weis, Ján Jakabovič, Miroslav Novota, Anton Gáplovský

**Affiliations:** 1 Faculty of Natural Sciences, Institute of Chemistry, Comenius University, Ilkovičova 6, Mlynská dolina CH-2, SK-842 15 Bratislava, Slovakia; 2 SYNKOLA, Ltd., Ilkovičova 6, 842 15, Bratislava, Slovakia; 3 Institute of Electronics and Photonics, Slovak University of Technology, Ilkovičova 3, SK-81219 Bratislava, Slovakia

**Keywords:** 4-azafluorenone, α-carboline, fluorescence, aggregation-induced emission, organic thin films and thin film devices

## Abstract

The emission properties of three 4-azafluorenone and five new α-carboline fluorophores in both solution and thin solid films were investigated. Fluorescence of the azafluorenone is clearly enhanced in thin solid films due to the presence of phenyl/biphenyl rotors, and these derivatives can be classified as green Aggregation-Induced Emission luminogens (AIEgens) with a non-emissive heteroaromatic core structure. Compared to azafluorenones, emission of α-carbolines is hypsochromically shifted to the blue region of the electromagnetic spectrum, and most of these derivatives exhibit strong violet-blue fluorescence in both solution and thin solid film layers. Further, the effective mobility and electroluminescence of new α-carbolines were investigated in prepared organic field-effect transistors and organic light-emitting diodes, respectively.

## 1. Introduction

The development of luminescent materials allowed gaining enormous knowledge that has undoubtedly promoted high-tech innovations and benefited the whole world [1]. For the vast majority of practical applications, luminophores are used as films and aggregates. Due to intense intermolecular π−π stacking interactions in the aggregate state, luminophores with aromatic rings often suffer from the phenomenon called aggregation-caused quenching (ACQ), particularly those with a disc or rod-like shape, and their emission is significantly reduced compared to the solution.

Fortunately, aggregation-induced emission (AIE) is another photophysical phenomenon associated with chromophore aggregation [1,2,3]. Whereas the emissive chromophores as molecular species are called “luminophores”, those non-emissive as molecules but emissive as aggregates are named “luminogens”. Upon aggregate formation, the emission of luminogens without independent double bonds is induced or rejuvenated by restricted intramolecular rotation (RIR) or vibratory motions (RIV; in-plane/out-of-plane bending, flapping, stretching, scissoring, wagging, twisting, rocking, etc., vibrations) or the synergistic effects of both motions (RIM; restricted intramolecular motion). As revealed by the Thomson Reuters Essential Science Indicators, AIE has been ranked No. 3 among the Top 100 Research Frontiers in the field of Chemistry and Materials Science in 2013, reflecting the increasing global interest towards AIE research [1]. The exploitation of aggregation-induced emission luminogens (AIEgens) is both of great academic and practical importance and can stimulate rapid development in the areas of optoelectronics, bio-imaging, nanoscience, etc. [2]. Only recently, Jeffrey S. Moore et al. demonstrated a simple, robust, and sensitive fluorescence-based approach for autonomous detection of damage in polymeric materials and composites enabled by AIE [4].

Compared with the pure hydrocarbon aromatic rings, heterocycle rings usually have lone pair electrons or empty orbits [3]. The AIE phenomenon of several heterocycle-based AIE systems with high stability due to their whole aromatic conjugated structure, such as phenyl-substituted siloles, pyrazines, pyrroles, and oxazoliums, has been investigated to date, although most of them are difficult to synthesize and purify [2]. Moreover, their emissions are mainly centered in the blue light region, which greatly hampers their practical applications in the field of biological imaging. This makes the development of new heterocycle-based AIEgens very promising, but challenging. Houk and coworkers predicted that N-heteroacenes could act as n-type semiconducting or electron-accepting materials through theoretical calculation [5]. Moreover, N-substituted acenes are believed to be less sensitive to degradation through oxidation or dimerization [6,7,8]. Actually, the number, position, and valence states of N atoms in backbones of azacenes have a strong effect on their properties [9,10,11].

Efficient blue organic light-emitting devices (OLEDs) play an important role in both full-color display and solid-state lighting [12,13,14]. Especially deep blue emitting materials not only act as the energy-transfer donor for a low energy dopant to generate green, red, or white light, but also increase the color gamut and reduce power consumption [15,16,17,18]. Fluorene has been intensively studied as an attractive building block due to its high photoluminescence efficiency, high carrier mobility, and easy modification. Many fluorene-based blue emitters and their derivatives have been reported and exhibited impressive device performance [18,19]. Similarly, the fluorenone unit as a strong electron acceptor was recently used in thermally-activated delayed fluorescence (TADF) materials [20]. TADF material is a kind of noble metal-free fluorescent material able to transform triplet excitons into singlet excitons through reverse intersystem crossing (RISC) to achieve 100% internal quantum efficiency in modern blue OLEDs [20,21,22,23]. Efficient blue OLEDs play an important role in both full-color display and solid-state lighting [18]. Although azafluorenone was discovered decades ago and its derivatives have received widespread attention in various research fields, the application of azafluorenone and its derivatives in OLEDs is rarely reported. Only recent results showed the high potential of azafluorenone and its analogues in the building of efficient host materials [24].

Phosphorescent organic light-emitting diodes (PhOLEDs) have attracted considerable attention due to high-efficiency device performance in flat-panel display applications. However, the development of high-efficiency blue PhOLEDs still remains a challenge because a larger energy bandgap and higher triplet energy are required for carrier transport materials and blue dopants, when compared to red- and green-emitting layer materials [25]. Among high triplet energy host materials, carbazole has been widely used as the high triplet energy moiety because of high triplet energy, easy synthesis, and facile functionalization. However, it was difficult to balance holes and electrons in the emitting layer just by using carbazole-based host materials due to the strong hole transport character of carbazole. Therefore, carboline was developed as the high triplet energy unit to compensate for the poor electron transport properties of carbazole [26]. However, further development of the high triplet energy host materials derived from carboline is required due to insufficient device performances.

**Scheme 1:**
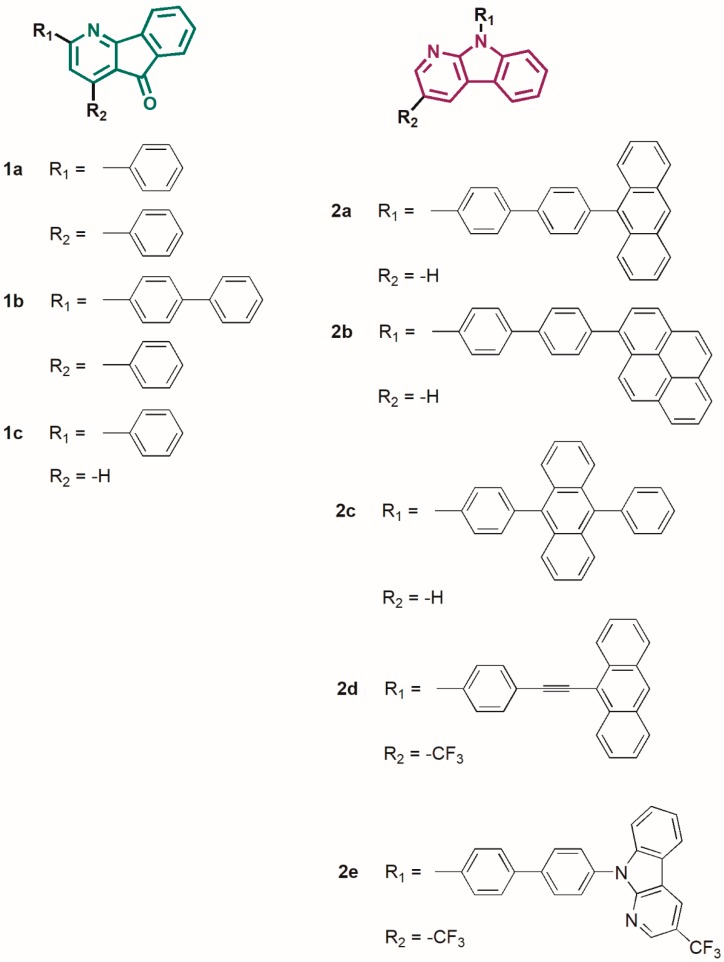
Molecular structure of the studied 4-azafluorenones **1a**–**1c** and α-carbolines **2a**–**2e**.

Herein, we report the results of a comprehensive investigation of the emission properties of three (one new) 4-azafluorenone and five new α-carboline fluorophores (Scheme 1) in both solution and thin solid films. Furthermore, the effective mobility and electroluminescence of new α-carbolines in prepared organic field-effect transistors and organic light-emitting diodes were also investigated.

## 2. Results and Discussion

### 2.1. Emission Characteristics

**Figure 1:**
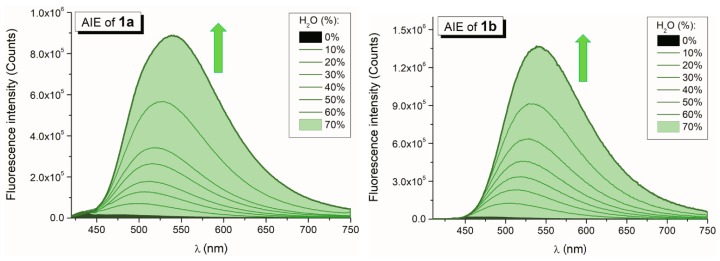
Fluorescence spectra of **1a** (**left**) and **1b** (**right**) in THF/water mixtures with different water fractions. AIE, aggregation-induced emission.

**Figure 2:**
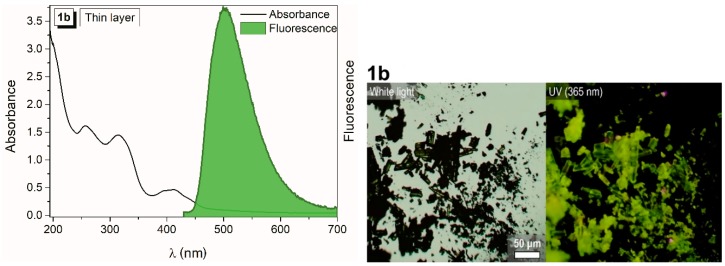
Absorption and emission spectrum of **1b** in the thin solid film layer on the quartz substrate (*λ*_EXC_ = *λ*_A_) (**left**) and micrograph of synthesized azafluorenone **1b** under visible (white) light and UV light (365 nm). The micrograph illustrates the crystals and fluorescence of **1b** in thin solid film (**right**).

Azafluorenone derivatives **1a**–**1c** exhibited light absorption in the UV-A region of electromagnetic spectrum and only poor emission in solution (low fluorescent quantum yields; Table 1). This behavior was very similar to 9-fluorenone with a near 100% triplet quantum yield of fluorenone in the non-polar solvent due to the efficient ISC (intersystem crossing) processes in the S1 state, particularly in the non-polar solvents [27,28]. Contrary to parent 4-azafluorenone, the increased amount of water as a co-solvent in **1a**–**1c** THF solutions led to a significant increase in emission intensity, and the 4-azafluorenones thus exhibited the typical feature of aggregation-induced emission luminogens (AIEgens; Figure 1 and Figure S1). Because the AIE appeared only in the presence of freely-rotatable phenyl or biphenyl substituents, we assumed that this behavior resulted particularly from the restriction of intramolecular rotations (RIR) and not the restriction of intramolecular vibrations (RIV). The presence of the freely-rotatable phenyl and biphenyl substituent was reflected particularly in almost equal values of solution emission maxima (*λ*_E_) of disubstituted **1a**, **1b**, and monosubstituted **1c** derivative (Table 1). At a water content above 70–80%, the compounds started to precipitate from the solution. The emission of **1a**–**1c** was clearly enhanced also in thin solid film layers, and the studied azafluorenones therefore can be classified as new green AIEgens with a non-emissive heteroaromatic core structure (Figure 2). Particularly the azafluorenone **1b** with a biphenyl rotor has a really high emission enhancement factor (*α*_AIE_), exceeding a value of 100 (Table 1).

The photophysical characteristics of new α-carboline derivatives **2a**–**2e** are shown in Table 2 and Table 3. Due to the non-planar geometry of the ground state, which hampered effective π-conjugation throughout the molecule (Table S1, Figures S2–S6), absorption of α-carbolines **2a**–**2c** and **2e** fell again into the near UV-A region of the electromagnetic spectrum. Compared to azafluorenone derivatives **1a**–**1c**, the fluorescence of **2a**–**2e** was hypsochromically shifted to the blue region of the electromagnetic spectrum in both the solution and thin solid film layers.

**Figure 3:**
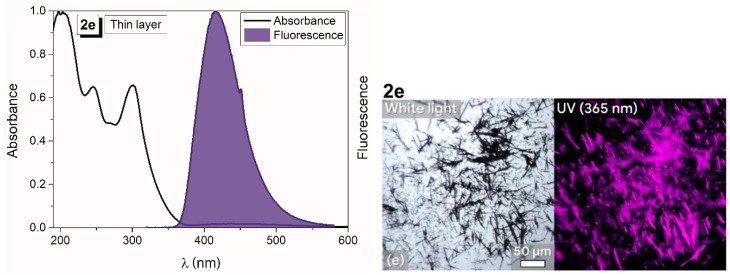
Absorption and emission spectrum of **2e** in the thin solid film layer on the quartz substrate (*λ*_EXC_ = *λ*_A_) (**left**) and the micrograph of synthesized carboline **2e** under visible (white) light and UV light (365 nm). The micrograph illustrates the crystals and fluorescence of **2e** in thin solid film (**right**).

Luminophores **2a** and **2b** preserved their solution emission efficiency also in thin solid films, and only carboline **2e** behaved as a violet-blue AIEgen with an *α*_AIE_ factor value of 7.4 (Figure 3).

**Figure 4:**
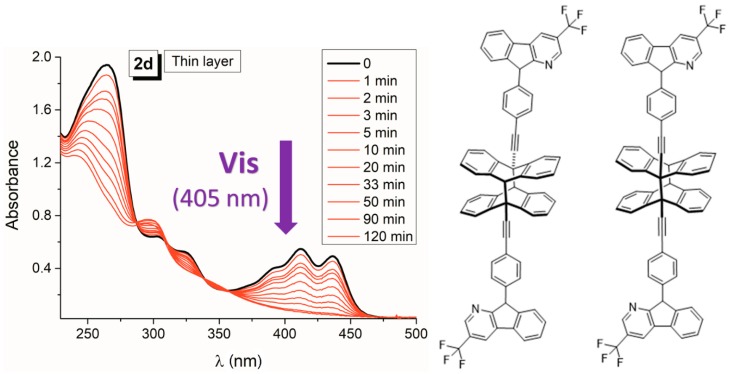
Evolution of the absorption spectrum of carboline **2e** in the thin solid film layer on the quartz substrate during its irradiation with Vis light of a 405-nm wavelength (four epoxy-encased LED diodes; LED405E from Thorlabs) (**left**) and the proposed structure of the anthracene dimer due to photochemically-allowed [4π_s_+ 4π_s_] dimerization through anthracene subunits during the **2e** thin solid film irradiation with Vis light (*λ*_irr_ = 405 nm) (**right**).

**Figure 5:**
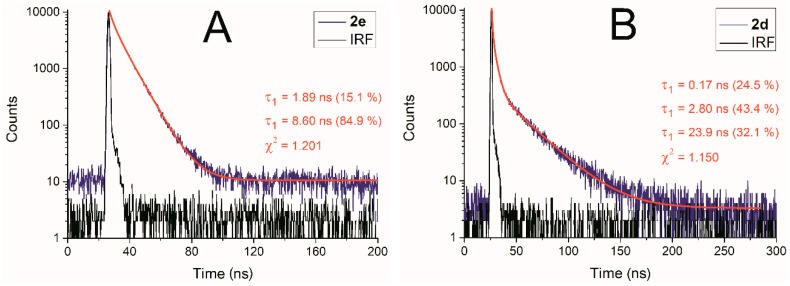
Fluorescence decay profiles of the carbolines **2e** (**A**) and **2d** (**B**)in thin solid films obtained using an excitation wavelength of 340 nm and 402.8 nm, respectively (monitored at emission maximum; IRF, instrument response function).

Carboline **2c** exhibited the aggregation-caused quenching (ACQ) phenomenon, and its fluorescent quantum yield significantly decreased from approximately 0.8 in solution to the value of 0.2 in the thin solid film (Table 3). As expected, luminophore **2d** with a rigid phenylethynyl spacer absorbed light in the violet region of the electromagnetic spectrum due to increased conjugation of anthracene with the phenylethynyl linker and exhibited really strong emission in the solid state with almost 100% efficiency (*Φ*_F_ ~ 1; Table 3). Interestingly, when irradiated with visible (Vis) light of a 405-nm wavelength, luminophore **2d** in thin solid film underwent a photochemically-allowed [4π_s_ + 4π_s_] dimerization through anthracene subunits, with the typical decrease of the long-wavelength absorption band in the Vis region and the appearance of a new strong light anthracene dimer absorption around 250 nm (Figure 4) [29]; contrary to the Diels–Alder dimer of 9-phenylethynylanthracene with the typical vibrational structure of the anthracene absorption band between 350 and 400 nm [30]. This Vis light-driven dimerization phenomenon in the solid state and its back thermally-driven reaction could be important for reversible optical writing and data storage applications [31,32,33]; however, the switching system needs deeper investigation, which is out of the scope of this paper. Bi- and tri-exponential fluorescence decay behavior in the solid films versus mono-exponential fluorescence decay in both polar and non-polar solutions, together with evident bathochromic shift of the emission maximum, clearly indicated aggregation of the carbolines **2a**–**2e** in the solid state (Table 3, Figure 5).

Although none of the studied carbolines exhibited the feature of delayed fluorescence (long fluorescence lifetime component due to the back ISC from the triplet to singlet excited state), the carboline luminophores **2a**–**2e** were further investigated as emissive layers in organic light-emitting diodes (OLEDs) and also as transport layers in organic field-effect transistors (OFETs).

### 2.2. Surface Potential Measurement

The surface potential evaluation by the Kelvin probe is recognized as a well-established method for estimation of charge transport energy levels. In principle, the surface potential depicts the energy difference between the work function of the metal substrate and the energy level of an organic semiconductor. Since the work function, 5.2 eV, of the selected metal (Pd) is close to the calculated HOMO level, the surface potential reflects hole transport through the metal/organic interface. Table 4 illustrates the HOMO energy levels of synthesized organic semiconductors. Subsequently, the optical energy gap was estimated using the Tauc plot from the absorbance spectra of the organic film deposited on the quartz substrate. Hence, the energies of LUMO levels, summarized in Table 4, were evaluated by the combination of the surface potential measurement and optical absorbance.

### 2.3. Organic Field-Effect Transistors

The output and transfer characteristics have been recorded for voltage ranging from −40 V to 40 V; however, the output current was significantly lower than 1 nA, which represents effective mobility on the level of 10^−6^ or lower. It should be noted that the same result provided a combination of low mobility and high contact resistance of the fabricated devices. Nevertheless, device optimization is beyond the scope of the present work.

Thermal stability is one of the key parameters for molecules used in organic electronics. As shown in Figures S7–S14, the studied 4-azafluorenone and α-carboline derivatives exhibited sufficient thermal stability, which ranged from 284 °C to 392 °C for 4-azafluorenones **1a**–**1c** and from 366 °C to 456 °C for α-carbolines **2a**–**2e**, respectively (the thermal stability was determined from the extrapolated onset temperature *T*_o_).

### 2.4. Organic Light-Emitting Diodes

**Figure 6:**
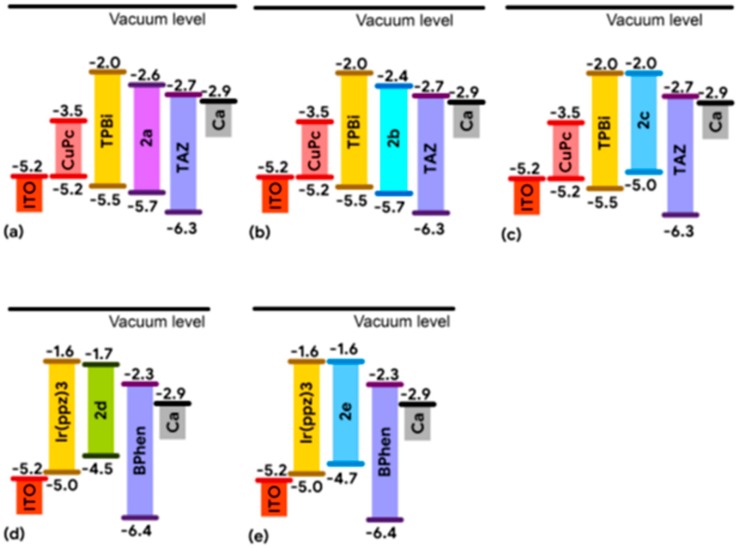
Energy band diagrams of OLED devices using (**a**) **2a**, (**b**) **2b**, (**c**) **2c**, (**d**) **2d**, and (**e**) **2e** as the emissive layer.

Figure 6 illustrates the energy band diagrams of the designed OLED devices. It should be noted here that since the deviation of HOMO for Compounds **2a**–**2e** varied from −4.5 to −5.7 eV, the design should be adjusted to the specific energy levels of material used as an emissive one.

**Figure 7:**
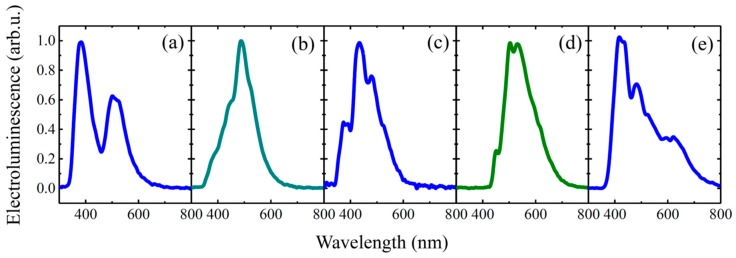
Normalized electroluminescence spectra of (**a**) **2a**, (**b**) **2b**, (**c**) **2c**, (**d**) **2d**, and (**e**) **2e**.

The electroluminescence (EL) spectra of fabricated OLED devices with carboline **2a**–**2e** emissive layers are depicted in Figure 7. It should be noted here that the EL spectra were independent of the applied voltage, and only intensity increased proportional to the current density. Hence, all plotted spectra were normalized for better comparison. Even though the devices were not stable for long-period measurement, the devices exhibited reproducible characteristics, and spectra were identical for the group of fabricated devices (eight devices for each derivative).

Table 4 summarizes the optical energy gap and the energy transitions corresponding to the observed electroluminescence peaks. In most cases, the energy of emitted light was slightly lower than the energy gap, which is a common phenomenon of emitting materials. Only the derivative **2a** exhibited separated EL with energy of 3.25 eV, which was higher than the energy gap. The origin of this emission should be in exciplex formation on the interface of the **2a** and 5-(4-biphenylyl)-3-(tert-butylphenyl)-1,2,4-triazole (TAZ), which serves as an electron transport and hole blocking layer. Furthermore, the OLED device with the **2d** emissive layer showed the presence of exciplex on the **2d**-4,7-diphenyl-1,10-phenanthroline (BPhen) interface. The observed emission was in good agreement with the difference of the BPhen LUMO level and **2d** HOMO level. As a result, the EL emission apparently differed from the photoluminescence emission due to the mutual interaction between the emissive layer and other layers in the OLED device.

**Figure 8:**
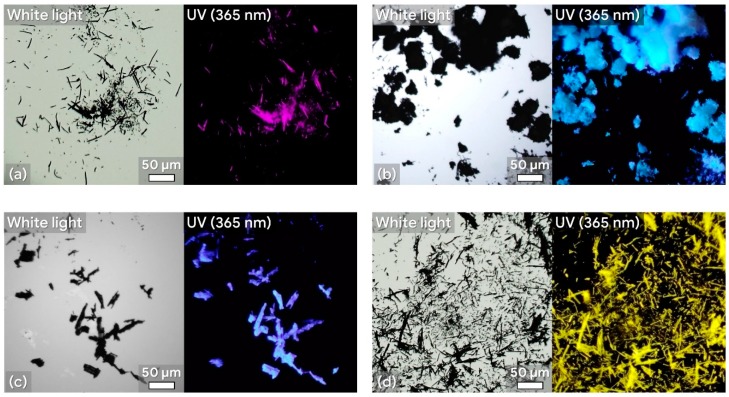
Micrographs of synthesized carboline derivatives **2a**–**2d** under visible (white) light and UV light (365 nm). The micrographs illustrate the crystals and photoluminescence of (**a**) **2a**, (**b**) **2b**, (**c**) **2c**, and (**d**) **2d**.

Micrographs depicted in Figure 8 illustrate crystals and photoluminescence. Obviously, the broad emission spectra of some derivates led to the white-like emission even though the blue regions were present there. Since the derivate **2d** did not exhibit blue emission, the color appeared yellowish.

## 3. Materials and Methods

### 3.1. Synthesis

All chemicals and solvents were purchased from Sigma-Aldrich (Merck KGaA, Darmstadt, Germany), Fluorochem (Derbyshire, UK) and Merck (Darmstadt, Germany) chemical companies. FTIR spectra were recorded on a Cary 630 FTIR (Agilent technologies, Santa Clara, CA, USA) using the ATR technique. Elemental analyses were obtained on an Elementar vario MICRO cube (Elementar, Langenselbold, Germany). Melting points were recorded on a Kofler apparatus Electrothermal IA-9200 (The Lab Warehouse, Grays, UK). NMR spectra were recorded in a 5-mm NMR tube on a Varian VNMRS 600-MHz spectrometer (600 MHz for ^1^H and 150 MHz for ^13^C, Agilent, Santa Clara, CA, USA) in DMSO-*d*_6_ or CDCl_3_, with tetramethylsilane (TMS) as an internal standard. High resolution mass spectrometry (HRMS) analyses were performed on a Thermo Scientific Orbitrap Fusion Mass Spectrometer (ThermoFisher Scientific, Waltham, MA, USA). Thermogravimetric (TGA) analysis was carried out on a LINSEIS STA PT 1600 analyzer (Linseis, Selb, Germany).

#### 3.1.1. 4-Azafluorenones

**Scheme 2:**
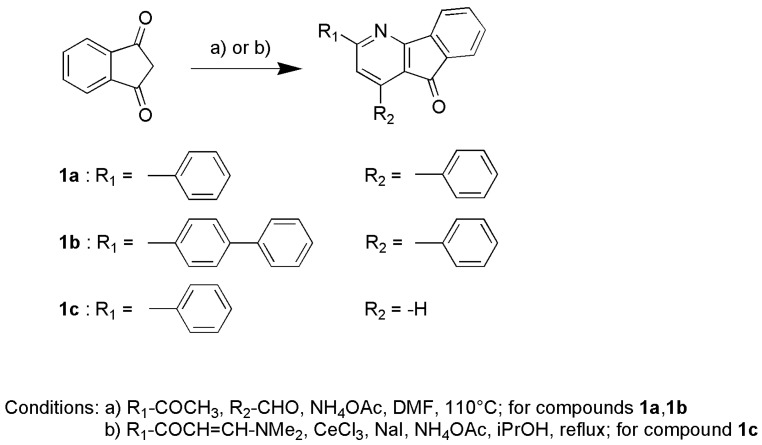
Synthesis of the studied 4-azafluorenones **1a**–**1c**.

Synthesis of **1a**–**1c**, general scheme (Scheme 2):

2,4-diphenyl-5*H*-indeno[1,2-*b*]pyridin-5-one (**1a**) 

Acetophenone (1.18 mL,1.216 g, 10 mmol, 1 mol eq.), benzaldehyde (1.01 mL, 1.05 g, 10 mmol, 1 mol eq.), and ammonium acetate (1.54 g, 20 mmol,2 mol eq.) were added to a stirred solution of 1*H*-indene-1,3(2*H*)-dione (1.462 g, 10 mmol, 1 mol eq.) in anhydrous DMF (40 mL) under argon. The mixture was warmed up to 100 °C and stirred five hours at this temperature. The reaction mixture was cooled to RT and DMF removed under reduced pressure. Water (20 mL) and EtOAc (20 mL) were added to the remaining wax and stirred for 30 min. The mixture was extracted with EtOAc (3 × 20 mL). The combined organic phases were dried over Na_2_SO_4_ and concentrated under reduced pressure. The crude product was isolated by LCC (50 g SiO_2_; DCM in Hex 0/100 to 100/0) [34,35].

*2,4-diphenyl-5H-indeno[1,2-b]pyridin-5-one*: yield 16.5%, yellow crystalline solid, m.p.: 167–169 °C; ^1^H-NMR (600 MHz, DMSO-*d_6_*): *δ* (ppm) 8.31 (m, 2H), 7.96 (d, *J* = 7.3 Hz, 1H), 7.83 (s, 1H), 7.76 (m, 2H), 7.73 (dtr, *J* = 7.7, 7.3 Hz, 1H), 7.65 (d, *J* = 7 Hz, 1H), 7.49–7.57 (m, 7H); ^13^C-NMR (150 MHz, DMSO-*d_6_*): *δ* (ppm) 190.6, 166.0, 160.5, 149.4, 142.6, 137.9, 135.8, 135.36, 135.32, 131.9, 130.8, 130.0, 129.8 (2C), 129.3 (2C), 128.5 (2C), 127.9 (2C), 124.0, 122.7, 121.5, 121.1; HRMS *m*/*z* calculated for C_24_H_15_NO [M + H]^+^: 334.1232, found: 334.1223; IR (neat): ῡ = 1710, 1550, 746, 687 cm^−1^.

2-([1,1′-biphenyl]-4-yl)-4-phenyl-5*H*-indeno[1,2-*b*]pyridin-5-one (**1b**)

Biphenyl-4-acetaldehyde (1.963 g, 10 mmol, 1 mol eq.), benzaldehyde (1.01 mL,10 mmol, 1 mol eq.), and ammonium acetate (1.54 g, 20 mmol, 2 mol eq.) were added to a stirred solution of 1*H*-indene-1,3(2*H*)-dione 1 (1.462 g, 10 mmol, 1 mol eq.) in anhydrous DMF (40 mL) under argon. The mixture was warmed up to 110 °C and stirred five hours at this temperature. The reaction mixture was cooled to RT and DMF removed under reduced pressure. Water (20 mL) and EtOAc (20 mL) were added to the remaining wax and stirred 30 min. The mixture was extracted with EtOAc (5 × 10 mL). The combined organic phases were dried over Na_2_SO_4_ and concentrated under reduced pressure. The crude product was isolated by LCC (50 g SiO_2_; Hex to DCM).

*2-([1,1′-biphenyl]-4-yl)-4-phenyl-5H-indeno[1,2-b]pyridin-5-one*: yield 17.6%, yellow crystalline solid, m.p.: 214–216 °C; ^1^H-NMR (600 MHz, DMSO-*d_6_*): *δ* (ppm) 8.44 (d, *J* = 8.5 Hz, 2H), 8.00 (d, *J* = 7.3 Hz, 1H), 7.92 (s, 1H), 7.86 (d, *J* = 8.5 Hz, 1H), 7.80–7.76 (m, 4H), 7.74 (dtr, *J* = 7.6, 7.3 Hz, 1H), 7.76 (d, *J* = 7.3 Hz, 1H), 7.56 (dtr, *J* = 7.6, 7.3 Hz, 1H), 7.54–7.48 (m, 5H), 7.41 (tr, *J* = 7.6, 7.3 Hz, 1H); ^13^C-NMR (150 MHz, DMSO-*d_6_*): *δ* (ppm) 190.6, 166.1, 160.0, 149.5, 142.6, 142.3, 139.7, 136.9, 135.8, 135.4, 135.3, 132.0, 130.0, 129.9 (2C), 129.5 (2C), 128.5 (4C), 128.4, 127.5 (2C), 127.2 (2C), 124.0, 122.7, 121.4, 121.2; HRMS *m*/*z* calculated for C_30_H_19_NO [M + H]^+^: 410.1544, found: 410.1537; IR (neat): ῡ = 1702, 1546, 746, 689 cm^−1^.

2-phenyl-5*H*-indeno[1,2-*b*]pyridin-5-one (**1c**)

Dimethylamino-1-phenyl-2-propen-1-one (876 mg, 5 mmol, 1 mol eq.), ammonium acetate (611 mg, 10 mmol, 2 mol eq.), sodium iodide (150 mg, 1 mmol, 0.2 mol eq.), and cerium chloride (370 mg, 1.5 mmol 0.3 mol eq.) were added to a stirred solution of 1*H*-indene-1,3(2*H*)-dione (730 mg, 5 mmol, 1 mol eq.) in anhydrous isopropanol (25 mL) under argon. The mixture was refluxed 24 h. The reaction mixture was cooled to RT, and isopropanol was removed under reduced pressure. Water (20 mL) and EtOAc (20 mL) were added to the remaining wax and stirred 30 min. The mixture was extracted with EtOAc (3 × 20 mL). The combined organic phases were dried overNa_2_SO_4_ and concentrated on a rotavap. The crude product was purified by flash liquid chromatography (FLC) (50 g SiO_2_; Hex to DCM) [36].

*2-phenyl-5H-indeno[1,2-b]pyridin-5-one*: yield 24%, yellow crystalline solid, m.p.: 148–150 °C, ^1^H-NMR (600 MHz, DMSO-*d_6_*): *δ* (ppm) 8.22 (d, *J* = 7.9 Hz, 2H), 8.04 (d, *J* = 7.9 Hz, 1H), 7.95 (d, *J* = 7.9 Hz, 1H), 7.93 (d, *J* = 7.3 Hz, 1H), 7.74–7.70 (m, 2H), 7.57–7.50 (m, 4H); ^13^C-NMR (150 MHz, DMSO-*d_6_*): *δ* (ppm) 191.3, 165.0, 161.1, 143.3, 138.0, 136.1, 135.3, 133.0, 131.9, 130.9, 129.4 (2C), 127.6 (2C), 126.7, 124.3, 121.2, 120.5; HRMS: *m*/*z* calculated for C_18_H_11_NO [M + H]^+^: 258.0919, found: 258.0911; IR (neat): ῡ = 1708, 1568, 1408, 745, 687 cm^−1^.

#### 3.1.2. α-Carbolines

**Scheme 3:**
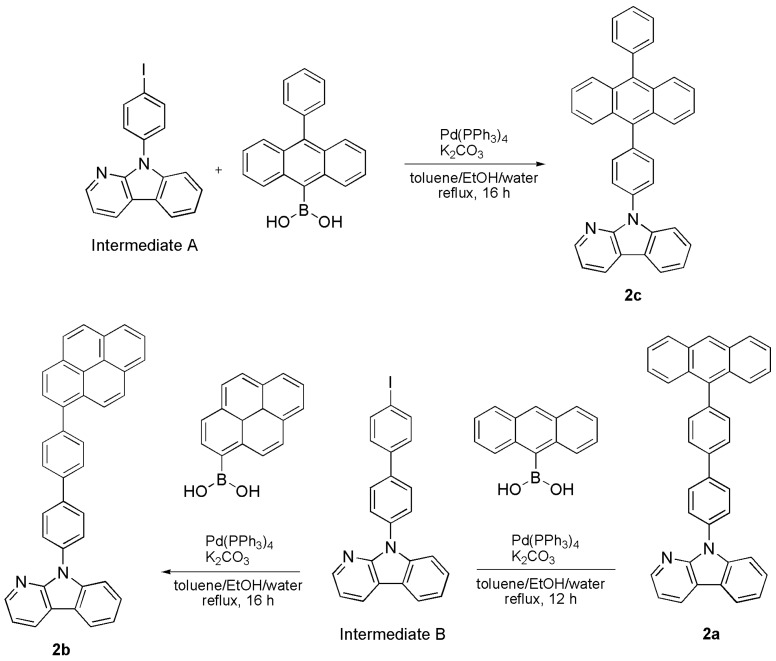
Synthesis of the studied α-carbolines **2a**–**2c**.

A. Synthesis of **2a**–**2c**, general scheme (Scheme 3):

Preparation of Intermediates A and B:

Preparation of 9-(4-iodophenyl)-9*H*-pyrido[2,3-*b*]indole (Intermediate A) and 9-(4′-iodo-[1,1′-biphenyl]-4-yl)-9*H*-pyrido[2,3-*b*]indole (Intermediate B)

General Procedure A: 9-(4′-iodo-[1,1′-biphenyl]-4-yl)-9*H*-pyrido[2,3-*b*]indole (Intermediate B)

To a solution of 9*H*-pyrido[2,3-*b*]indole (1.00 g, 5.95 mmol, 1 mol eq.) in toluene/dry (60 mL) were added 3.62 g of 4,4′-diiodobiphenyl (8.93 mmol, 1.5 mol eq.), 6.32 g of K_3_PO_4_ (7.15 mmol, 5 mol eq.), 0.36 mL of trans-1,2-diaminocyklohexane (340 mg, 2.98 mmol, 0.5 mol eq.), and 225 mg of CuI (1.19 mmol., 0.2 mol eq.). The resulting mixture was refluxed under argon for 18 h. After consumption of all starting material (monitored by TLC), the reaction mixture was cooled to RT, filtered through Celite, and the filtration cake was washed with DCM. The crude product was isolated by FLC (CombiFlash; 330 g SiO_2_; 100 mL/min; Hex/EtOAc 3:1 *v*/*v*).

*9-(4′-iodo-[1,1′-biphenyl]-4-yl)-9H-pyrido[2,3-b]indole:* yield 1.06 g, 40%, white solid; m.p.: 210.1–212.3 °C [EtOAc/Hex]; ^1^H-NMR (CDCl_3_, 600 MHz): δ (ppm) 8.51 (dd, *J* = 4.8, 1.6 Hz, 1H), 8.41 (dd, *J* = 7.6, 1.6 Hz, 1H), 8.15 (d, *J* = 7.8 Hz, 1H), 7.82 (dm, *J* = 8.4 Hz, 2H), 7.79 (dm, *J* = 8.6 Hz, 2H), 7.74 (dm, *J* = 8.6 Hz, 2H), 7.56 (d, *J* = 8.2 Hz, 1H), 7.50 (ddd, *J* = 8.2, 7.0, 1.0 Hz, 1H), 7.42 (dm, *J* = 8.4 Hz, 2H), 7.36 (ddd, *J* = 8.0, 7.0, 1.0 Hz, 1H), 7.27–7.25 (m, 1H); ^13^C-NMR (CDCl_3_, 150 MHz): δ (ppm) 151.8, 146.3, 140.0, 139.9, 139.3, 138.0, 135.8, 129.0, 128.5, 128.2, 127.7, 127.1, 121.0, 120.93, 120.90, 116.5, 116.2, 110.4, 93.4; HRMS: *m*/*z* calculated for C_23_H_15_N_2_I [M + H]^+;^ 447.0353, found: 447.0352.

9-(4-iodophenyl)-9*H*-pyrido[2,3-*b*]indole (Intermediate A)

Intermediate A was prepared according to General Procedure A.

*9-(4-iodophenyl)-9H-pyrido[2,3-b]indole:* yield 620 mg, 47%, white solid; m.p.: 145.0–146.7 °C [EtOAc/Hex]; ^1^H-NMR (CDCl_3_, 600 MHz): δ (ppm) 8.47 (dd, *J* = 5.0, 1.6 Hz, 1H), 8.38 (dd, *J* = 7.7, 1.6 Hz, 1H), 8.12 (ddd, *J* = 8.6, 7.9, 0.9 Hz, 1H), 7.93 (dm, *J* = 8.6 Hz, 2H), 7.50–7.47 (m, 2H), 7.43 (dm, *J* = 8.6 Hz, 2H), 7.37–7.32 (m, 1H), 7.25 (q, *J* = 7.7 Hz, 1H); ^13^C-NMR (CDCl_3_, 150 MHz): δ (ppm) 151.8, 146.6, 139.8, 139.0, 136.2, 129.3, 128.7, 127.3, 121.3, 121.2, 116.8, 116.6, 110.4, 92.7; HRMS: *m*/*z* calculated for C_17_H_11_N_2_I [M + H]^+;^ 371.0040, found: 371.0039.

**Scheme 4:**
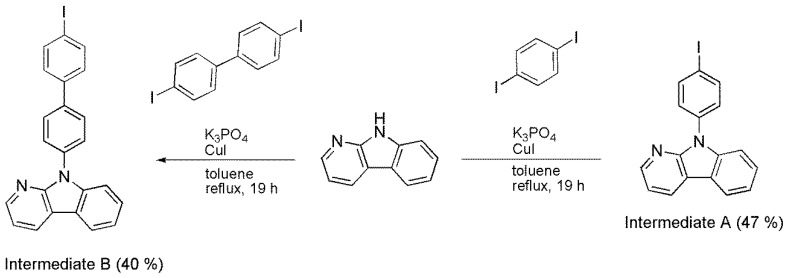
Synthesis of the intermediates A and B.

Reaction scheme (Scheme 4):

Preparation of final products **2a**–**2c** (Suzuki coupling reactions):

9-(4′-(anthracen-9-yl)-[1,1′-biphenyl]-4-yl)-9*H*-pyrido[2,3-*b*]indole (**2a**)

General Procedure B

In a 50-mL round-bottom flask, (intermediate B) (781 mg, 1.75 mmol, 1 mol eq.), anthracene-9-boronic acid (583 mg, 2.63 mmol, 1.5 mol eq.), aqueous solution of K_2_CO_3_ (2.2 mL, 4.38 mmol, 2.5 mol eq.), EtOH (8 mL), and toluene (16 mL) were stirred under argon for 5 min. Then, Pd(PPh_3_)_4_ (61 mg, 0.053 mmol, 0.05 mol eq.) was added. The flask was evacuated and backfilled with argon three times, and the resultant reaction mixture was stirred at reflux (oil bath) for 18 h. After cooling to RT, the solvent was evaporated under reduced pressure. The solid material was dissolved in DCM (30 mL), washed with water (3 × 10 mL), and dried over Na_2_SO_4_. After filtration and evaporation of the solvent, the crude product was purified by FLC (CombiFlash, 120 g SiO_2_, 75 mL/min, Hex/DCM 1:1 *v*/*v*).

*9-(4′-(anthracen-9-yl)-[1,1′-biphenyl]-4-yl)-9H-pyrido[2,3-b]indole:* yield 575 mg, 66%, pale yellow solid, m.p.: 312.4–316.8 °C [EtOAc/Hex]; ^1^H-NMR (CDCl_3_, 600 MHz): δ (ppm) 8.55 (dd, *J* = 4.8, 1.6 Hz, 1H), 8.53 (s, 1H), 8.43 (dd, 7.7, 1.6 Hz, 1H), 8.18–8.15 (m, 1H), 8.08 (dm, *J* = 8.6 Hz, 2H), 7.99 (dm, *J* = 8.6 Hz, 2H), 7.89 (dm, *J* = 8.2 Hz, 2H), 7.82 (dm, *J* = 8.6 Hz, 2H), 7.78 (dd, *J* = 8.9, 0.5 Hz, 2H), 7.62 (d, *J* = 8.3 Hz, 1H), 7.56 (dm, *J* = 8.2 Hz, 2H), 7.53 (ddd, *J* = 8.3, 7.2, 1.1 Hz, 1H), 7.51–7.47 (m, 2H), 7.42–7.36 (m, 3H), 7.28 (q, *J* = 7.7 Hz, 1H); ^13^C-NMR (CDCl_3_, 150 MHz): δ (ppm) 146.1, 140.3, 140.1, 139.5, 138.1, 136.6, 135.4, 131.8, 131.4, 130.2, 128.7, 128.5, 128.4, 127.7, 127.18, 127.15, 126.8, 125.4, 125.2, 121.03, 121.0, 120.9, 116.1, 110.6; HRMS: *m*/*z* calculated for C_37_H_24_N_2_ [M + H]^+^: 497.2012, found: 497.2012.

9-(4′-(pyren-1-yl)-[1,1′-biphenyl]-4-yl)-9*H*-pyrido[2,3-*b*]indole (**2b**)

Compound **2b** was prepared according to General Procedure B.

*9-(4′-(pyren-1-yl)-[1,1′-biphenyl]-4-yl)-9H-pyrido[2,3-b]indole:* yield 308 mg, 53%, pale orange solid, m.p.: 235.6–237.4 °C [EtOAc/Hex]; ^1^H-NMR (CDCl_3_, 600 MHz): δ (ppm) 8.55 (dd, *J* = 4.8, 1.6 Hz, 1H), 8.43 (dd, *J* = 7.6, 1.6 Hz, 1H), 8.3 (d, *J* = 9.3 Hz, 1H), 8.27 (d, *J* = 7.8 Hz, 1H), 8.22 (d, *J* = 7.7 Hz, 1H), 8.19 (d, *J* = 7.4 Hz, 1H), 8.17 (d, *J* = 7.8 Hz, 1H), 8.12 (m, 1H), 8.08 (d, *J* = 9.3 Hz, 1H), 8.06 (d, *J* = 7.9 Hz, 1H), 8.04 (t, *J* = 7.6 Hz, 1H), 7.97 (dm, *J* = 8.2 Hz, 2H), 7.88 (dm, *J* = 8.2 Hz, 2H), 7.81 (dm, *J* = 8.2 Hz, 2H), 7.78 (dm, *J* = 8.2 Hz, 2H), 7.62 (d, *J* = 8.2 Hz, 1H), 7.53 (ddd, *J* = 8.2, 7.2, 1.0 Hz, 1H), 7.38 (ddd, *J* = 8.2, 7.2, 1.0 Hz, 1H), 7.28 (dd, *J* = 7.7 Hz, 1H); ^13^C-NMR (CDCl_3_, 150 MHz): δ (ppm) 145.9, 140.5, 140.3, 140.1, 139.4, 137.2, 131.5, 131.2, 131.0, 130.7, 128.53, 128.51, 127.7, 127.61, 127.60, 127.5, 127.4, 127.3, 127.2, 126.0, 125.3, 125.2, 125.0, 124.93, 124.90, 124.7, 121.1, 120.8, 116.1, 110.6; HRMS: *m*/*z* calculated for C_39_H_24_N_2_ [M + H]^+^: 521.2012, found: 521.2012.

9-(4-(10-phenylanthracen-9-yl)phenyl)-9*H*-pyrido[2,3-*b*]indole (**2c**)

Compound **2c** was prepared according to General Procedure B.

*9-(4-(10-phenylanthracen-9-yl)phenyl)-9H-pyrido[2,3-b]indole:* yield 371 mg, 92%, pale yellow solid, m.p.: 265.5–268.0 °C [EtOAc/Hex]; ^1^H-NMR (CDCl_3_, 600 MHz): δ (ppm) 8.61 (dd, *J* = 4.8, 1.5 Hz, 1H), 8.47 (dd, *J* = 7.7, 1.5 Hz, 1H), 8.2 (d, *J* = 7.7 Hz, 1H), 7.95 (dm, *J* = 8.2 Hz, 2H), 7.9 (dm, *J* = 8.3 Hz, 2H), 7.78–7.16 (m, 5H), 7.65–7.60 (m, 2H), 7.6–7.55 (m, 2H), 7.53–7.49 (m, 2H), 7.43–7.35 (m, 5H), 7.32 (q, *J* = 7.7 Hz, 1H); ^13^C-NMR (CDCl_3_, 150 MHz): δ (ppm) 146.3, 140.1, 139.0, 138.3, 137.5, 136.2, 135.6, 132.6, 131.3, 129.91, 128.6, 128.4, 127.5, 127.2, 127.1, 127.0, 125.2, 125.1, 121.1, 121.0, 116I.8, 116.2, 110.7; HRMS: *m*/*z* calculated for C_37_H_24_N_2_ [M + H]^+;^ 497.2012, found: 497.2011.

**Scheme 5:**
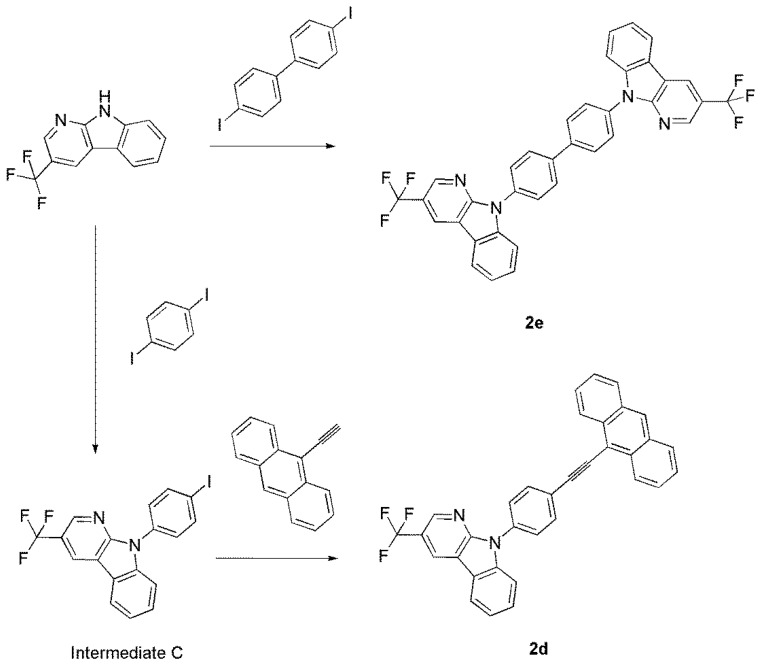
Synthesis of the studied α-carbolines **2d** and **2e**.

B. Synthesis of **2d** and **2e**, general scheme (Scheme 5):

9-(4-iodophenyl)-3-trifluoromethyl-9*H*-pyridino[2,3-*b*]indole (Intermediate C)

A portion of 3-trifluoromethyl-9*H*-pyridino[2,3-*b*]indole^1^ (826 mg, 3.5 mmol, 1 mol eq.), 1,4-diiodonenzene (1.24 g, 4.375 mmol, 1.25 mol eq.), K_3_PO_4_(3.715 g, 17.5 mmol, 5 mol eq.), CuI (9.135 g, 0.7 mmol, 0.5 mol eq.), and trans-1,2-diaminocyclohexane (200 mg, 0.21 mL, 1.75 mmol, 0.5 mol eq.) were suspended in dry toluene (25 mL) under an argon atmosphere, and the mixture was stirred at 110 °C for 18 h. After finishing the reaction, the mixture was filtered through Celite and washed with DCM. The organic phase was concentrated on a rotavap. The crude product was purified by FLC (CombiFlash, 120 g SiO_2_, 75 mL/min, Hex/DCM 1:1 *v*/*v*).

*9-(4-iodophenyl)-3-trifluoromethyl-9H-pyridino[2,3-b]indole:* yield 65.1%, white solid, m.p.: 151.4–153.2 °C; ^1^H-NMR (600 MHz, DMSO-*d*_6_): *δ* 8.72 (d, *J* = 1.2 Hz, 1H), 8.61 (d, *J* = 1.8 Hz, 1H), 8.17 (d, *J* = 7.8 Hz, 1H), 7.97 (m, 2H), 7.56 (dtr, *J* = 8.4 Hz, 7.0 Hz, 1H), 7.49 (d, *J* = 8.4 Hz, 1H), 7.43–7.40 (m, 3H); ^13^C-NMR (150 MHz, DMSO-*d_6_*): *δ* (ppm) 152.8, 143.68, 143.65, 140.5, 138.9, 135.3, 130.0, 128.9, 127.3, 126.7, 124.5, 122.84, 122.35, 120.41, 118.76 (q), 115.93, 111.01, 94.32; HRMS: *m*/*z* calculated for C_18_H_10_F_3_IN_2_ [M + H]: 438.914, found: 438.9913.

**Scheme 6:**
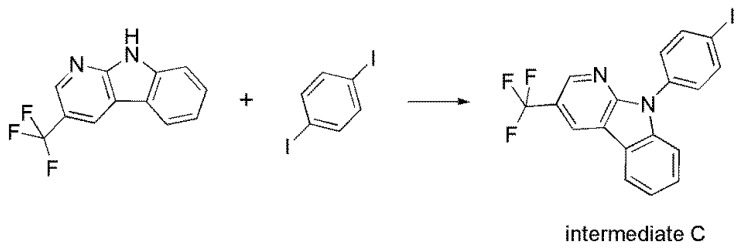
Synthesis of the intermediate C.

Reaction scheme (Scheme 6):

*Note:* The corresponding starting α-carboline was prepared according to the literature [37] in two steps with a satisfactory yield.

9-[4-(anthracene-9′-ethynyl)phenyl]- 3-trifluoromethyl-9*H*-pyridino[2,3-*b*]indole (**2d**)

9-(4-iodophenyl)-3-trifluoromethyl-9*H*-pyridino[2,3-*b*]indole (391 mg, 1.0 mmol, 1 mol eq.), 9-ethynylanthracene (242 mg, 1.2 mmol, 1.2 mol eq.), and PdCl_2_(PPh_3_)_2_ (20 mg, 0.03 mmol, 3 mol%) were dissolved in freshly-degassed triethylamine (10 mL) under argon atmosphere. After 5 min, copper (I) iodide (11.8 mg, 0.025 mmol, 2.5 mol%) was added. The reaction mixture was stirred at 70 °C for 16 h. The crude product was purified by FLC (CombiFlash 50 g silica gel) and gave the title compound (145 mg, 56.6%) as a yellow solid. The chromatographed product was crystallized from EtOAc (yellow powder, 84 mg. 32.78%).

*9-[4-(anthracene-9*′*-ethynyl)phenyl]-3-trifluoromethyl-9H-pyridino[2,3-b]indole:* yield: 32.78%, yellow powder, m.p.: 242.0–244.5 °C, ^1^H-NMR (600 MHz, DMSO-*d*_6_): *δ* 9.20 (d, *J* = 1 Hz, 1H), 8.86 (d, *J* = 1 Hz, 1H), 8.76 (s, 1H), 8.68 (d, *J* = 8.4 Hz, 2H), 8.52 (d, *J* = 7.8 Hz, 2H), 8.22 (d, *J* = 8.4 Hz, 2H), 8.16 (d, *J* = 8.4 Hz, 2H), 7.90 (d, *J* = 8.4 Hz, 2H), 7.76 (dt, *J* = 7.2, 1.0 Hz, 2H), 7.67–7.63 (m, 4H), 7.50 (dt, *J* = 7.2, 1.0 Hz, 1H); ^13^C-NMR (150 MHz, DMSO-*d_6_*): *δ* (ppm) 153.2, 143.9 (t, *J* = 3.9 Hz), 140.8, 135.7, 133.2, 132.9, 131.4, 129.0, 128.4, 128.3, 127.5, 127.0, 126.9, 125.9, 125.9, 125.8, 125.8, 123.7, 122.1, 121.7, 119.8, 119.6, 117.1, 116.1, 11.1, 100.1, 87.7; HRMS: *m*/*z* calculated for C_34_H_19_F_3_N_2_ [M + H]^+^: 513.1573, found: 513.1574.

4,4′-Bis(3-trifluoromethyl-9*H*-pyridino[2,3-*b*]indole-9-yl)-1,1′-biphenyl (**2e**)

A portion of 3-trifluoromethyl-9H-pyridino[2,3-*b*]indole (605 mg, 2.50 mmol, 2.5 mol eq.), 4,4′-diiodobiphenyl (507 mg, 1.0 mmol, 1 mol eq.), K_3_PO_4_(5.31 g, 25 mmol, 20 mol eq.), CuI (80 mg, 0.5 mmol, 0.4 mol eq.), and trans-1,2-diaminocyclohexane (286 mg, 0.30 mL, 2.5 mmol, 2 mol eq.) were suspended in dry toluene (36 mL) under an argon atmosphere, and the mixture was stirred at 110 °C for 18 h. After finishing the reaction, the mixture was filtered through Celite and washed with DCM. The crude product was purified by FLC (CombiFlash, 120 g SiO_2_, 75 mL/min, Hex/DCM 3:1 to 2:3 *v*/*v*).

*4,4′-Bis(3-trifluoromethyl-9H-pyridino[2,3-b]indole-9-yl)-1,1′-biphenyl:* yield 420 mg, 67.46%, white solid, m.p.: >325 °C (dec) °C, ^1^H-NMR (600 MHz, DMSO-*d*_6_): *δ* (ppm) 9.20 (d, *J* = 1.8 Hz, 1H), 8.85 (d, *J* = 1.8 Hz, 1H), 8.51 (d, *J* = 7.8 Hz, 1H), 8.12 (dd, *J* = 8.4, 1.8 Hz, 2H), 7.88 (d, *J* = 8.4 Hz, 2H), 7.66–7.63 (m, 1H), 7.49 (t, *J* = 7.8 Hz, 1H); not sufficiently soluble for ^13^C-NMR in either DMSO-*d*_6_ or CDCl_3_; HRMS: *m*/*z* calculated for C_36_H_20_F_6_N_4_ [M + H]^+^: 623.1665, found: 623.1665.

### 3.2. Spectroscopic Measurements

Electronic absorption spectra were obtained on an Agilent 8453 diode array spectrophotometer (Hewlett Packard, Palo Alto, CA, USA). The solvents used were HPLC or UV spectroscopy grade (MeOH, for spectroscopy, Acros Organics, Geel, B; CHCl_3_, Uvasol^®^, Merck, Darmstadt, Germany) and were used without further purification. Solution fluorescence was measured in a 1-cm cuvette with an FSP 920 (Edinburgh Instruments, Livingston, UK) spectrofluorometer in a right-angle arrangement. Fluorescence of thin solid film layers on quartz substrate (1 cm × 3 cm) was measured in front-face arrangement (FSP 920 spectrofluorometer; Edinburgh Instruments, UK). The fluorescent quantum yield (*Φ*_F_) of the studied compounds in solution or in thin solid film layer was determined by Equations (1) and (2) using an integrating sphere (Edinburgh Instruments):

(1) $$\Phi_{F}^{X}=\frac{L_{\mathrm{Sam}}}{E_{\mathrm{Ref}}-E_{\mathrm{Sam}}}\left(\%\right)$$

corrected to re-absorption by:

(2) $$\Phi_{F}=\frac{\Phi_{F}^{X}}{1-a+a\Phi_{F}^{X}/100}\;\left(\%\right)$$

where *L*_Sam_ is the area under the detected spectrum in the part of the spectrum where sample emission occurs, *E*_Ref_ is the area under the reflection part of the detected spectrum using the pure solvent (or uncovered quartz substrate) as the reference material (diffuse reflectance), *E*_Sam_ is the area under the reflection part of the detected spectrum after absorption by the sample, and *a* is the reabsorbed area. The time-resolved fluorescence measurements were performed on an FSP 920 (Edinburgh Instruments, UK) spectrofluorometer with a time-correlated single-photon counting (TCSPC) module and a red-sensitive high-speed photomultiplier in Peltier housing, featuring a Hamamatsu H5773-04 detector (R928P detector; Edinburgh Photonics, Livingston, UK). The excitation source was a 342.0-nm picosecond pulsed LED (Model EPLED-340; pulse width: 950.3 ps; Edinburgh Photonics, UK) or a 402.8-nm picosecond pulsed diode laser (Model EPL-405; pulse width: 60.5 ps; Edinburgh Photonics, UK). Fluorescence decay (lifetime) of thin solid film layers on the quartz substrate (1 cm × 3 cm) was measured in front-face arrangement. Reconvolution fit analysis software (F900, Edinburgh Instruments) was used for lifetime data analysis.

### 3.3. Organic Thin Films and Thin Film Devices

Organic thin films and thin film devices were fabricated by thermal evaporation under a high vacuum. All solid substrates such as quartz, glass covered by indium tin oxide (ITO), or silicon wafers, were cleaned in an ultrasonic bath subsequently by acetone, isopropyl alcohol, 20 vol% ethanolamine aqueous solution, and in deionized water prior to further processing. UV/ozone cleaning (UV-2 by SAMCO, Santa Clara, CA, USA) was performed just prior to thin film deposition to remove residual organic contamination. Then, a 100 nm-thick organic layer was thermally evaporated in a vacuum with a pressure lower than 10^−4^ Pa with a fixed deposition rate of 3 nm/min (SPECTROS 100 by Kurt J. Lesker, Jefferson Hills, PA, USA) controlled by a quartz crystal microbalance. The organic film thickness was verified by a mechanical profilometer (Dektak 150 by Bruker, Billerica, MA, USA).

Copper phthalocyanine (CuPc), 2,2′,2′′-(1,3,5-benzinetriyl)-tris(1-phenyl-1-H-benzimidazole) (TPBi), and 4,7-diphenyl-1,10-phenanthroline (BPhen) were supplied by Sigma-Aldrich, whereas 3-(biphenyl-4-yl)-5-(4-tert-butylphenyl)-4-phenyl-4H-1,2,4-triazole (TAZ) and 4,7-diphenyl-1,10-phenanthroline (BPhen) were provided by Ossila company. All materials were used without further purification. The CuPc film of 5 nm was used as a hole injection layer only, whereas other layers were designed as hole-transport layers or electron-transport and hole-blocking layers.

The optical and fluorescence microscopy images were taken by an optical microscope with a 1000× objective and large working distance under white light or UV (365 nm) illumination. Distance/size calibration was done using a 1951 USAF resolution test chart.

The energy level of the highest occupied molecular orbital (HOMO) was estimated using the surface potential measurement by means of the Kelvin probe method. A highly sensitive electrostatic voltmeter 320C and Kelvin probe electrode (Model 3250 by TREK Inc., Lockport, NY, USA) that allowed measuring of surface potential with an accuracy of 1 mV were used. The probe was situated in air approximately 1 mm above the organic layer surface. To perform the surface potential measurement, the palladium layer (100 nm) was deposited on the quartz substrate (1 cm × 3 cm), whereas the organic layer was subsequently deposited on half of the surface to record the surface potential profile and energy difference between the Fermi energy of the metal and the HOMO level of the organic semiconductor. We decided to use palladium due its environmental stability, as well as the well-defined Fermi energy of polycrystalline film (5.22 eV).

The organic light-emitting diodes (OLEDs) were used for estimation of electroluminescence spectra. The ITO was used as the transparent anode for hole injection, whereas the calcium protected by a silver layer was applied as the metal cathode for electron injection. The hole-injection, hole-transport, electron-transport, and electron-injection layers were selected in accordance with the specific values of the HOMO and LUMO energy levels of the emissive layer. Electroluminescence spectra were recorded in a nitrogen atmosphere by a spectrometer (AvaSpec-2048 FiberOptic Spectrometer by Avantes, Apeldoorn, The Netherlands).

The effective mobility was evaluated using organic field-effect transistor (OFET) devices with top-contact bottom-gate geometry. The silicon wafers with a 110-nm thermally-grown silicon dioxide (SiO_2_) layer as a substrate and gate electrode were used. Organic semiconductors were deposited onto a bare and 1,1,1,3,3,3-hexamethyldisilazane-modified (HMDS, Sigma-Aldrich) SiO_2_ surface to verify the film transport properties deposited on the hydrophilic, as well as the hydrophobic gate insulator. After the deposition of the semiconducting layer, copper was thermally evaporated through the shadow mask to form the source and drain electrodes. The OFET devices had channel lengths *L* and a width *W* of 50~200 μm and 2.5 mm, respectively. All output and transfer characteristics were measured in a nitrogen atmosphere using a semiconductor parameter analyzer (B1500A by Keysight, Santa Rosa, CA, USA).

### 3.4. Quantum-Chemical Calculations

Ground state molecular geometry was optimized at the B3LYP/G-311G* level of theory using Spartan ’10, Version 1.1.0 (Wavefunction, Inc., Irvine, CA, USA).

## 4. Conclusions

This paper investigated the emission properties of three 4-azafluorenone and five α-carboline fluorophores in solution and thin solid film layers. Azafluorenone derivatives exhibited typical features of AIEgens with the emission enhancement factor ranging from 10 to 150, whereas most of the α-carboline fluorophores preserved their strong violet-blue solution emission also in thin solid films. Although the prepared α-carbolines had low effective mobility in prepared organic field-effect transistors and the OLED devices with the α-carbolines as emissive layers were not stable for long-period measurement, both types of compounds could represent an important class of new fluorophores/fluorogens in the blue/green emission region or host materials in blue phosphorescent organic light-emitting diodes (PhOLEDs).

## Figures and Tables

**Table 1:** Photophysical properties of the studied azafluorenone derivatives **1a**–**1c**.

Cpd	*λ*_A_ (nm)	*λ*_E_ (nm)	*Φ*_F_ (%)		*α*_AIE_
	Soln	Soln	Soln	Film	
**1a**	386	475	1.0	11.2	11.2
**1b**	388	477	0.5	75.0	150.0
**1c**	376	476	1.0	26.1	26.1

Cpd, compound; *λ*_A_, absorption maximum; *λ*_E_, emission maximum; *Φ*_F_, fluorescent quantum yield; *α*_AIE_, emission enhancement factor (*α*_AIE_ = *Φ*_F(Film)_/*Φ*_F(Solution)_); Soln, THF solution.

**Table 2:** Photophysical (absorption and luminescent) properties of the studied carboline derivatives **2a**–**2e**.

Cpd	*λ*_A_ (nm)		*λ*_F_ (nm)		
	CHCl_3_	MeOH	CHCl_3_	MeOH	Film
**2a**	368	365	425/415 *	408/420 *	450
**2b**	340	342	407	407	455
**2c**	376	372	422/433 *	427/413 *	460
**2d**	403	399	437	432	450
**2e**	285	294	401	414	420

*λ*_A_, long-wavelength absorption maximum; *λ*_F_, fluorescence maximum; *, peak with a slightly smaller intensity due to the vibrational structure of the emission band.

**Table 3:** Fluorescence characteristics of the studied carboline derivatives **2a**–**2e**.

Cpd	*Φ*_F CHCl3_	*Φ*_F CH3OH_	*Φ*_F ss_	τ(CHCl_3_) (ns)	τ(CH_3_OH) (ns)	τ(ss) (ns)
**2a**	0.58	0.29	0.49	τ = 3.52 χ^2^ = 1.181	τ = 3.47 χ^2^ = 1.161	τ_1_ = 1.58 (23.0%) τ_2_ = 4.07 (77.0%) χ^2^ = 1.194
**2b**	0.48	0.53	0.41	τ = 2.91 χ^2^ = 1.149	τ = 5.09 χ^2^ = 1.163	τ_1_ = 0.64 (65.1%) τ_2_ = 2.58 (29.0%) τ_3_ = 13.1 (5.9%) χ^2^ = 1.124
**2c**	0.94	0.70	0.23	τ = 5.05 χ^2^ = 1.172	τ = 5.11 χ^2^ = 1.159	τ_1_ = 1.30 (48.7%) τ_2_ = 3.86 (51.3%) χ^2^ = 1.113
**2d**	0.68	0.74	1.00	τ = 2.95 χ^2^ = 1.155	τ = 3.49 χ^2^ = 1.135	τ_1_ = 0.17 (24.5%) τ_2_ = 2.80 (43.4%) τ_3_ = 23.9 (32.1%) χ^2^ = 1.150
**2e**	0.07	0.07	0.52	Low signal intensity	Low signal intensity	τ_1_ = 1.89 (15.1%) τ_2_ = 8.60 (84.9%) χ^2^ = 1.201

*Φ*_F_, fluorescent quantum yield; τ, fluorescence lifetime; χ^2^, reduced chi-squared (quality of fluorescence lifetime fitting) %, relative fluorescence intensity of the corresponding component; ss, solid state.

**Table 4:** Comparison of optical energy gaps and electroluminescence peaks, together with the energy of frontier orbitals. EL, electroluminescence.

Cpd	*E*_g,opt_ (eV)	EL (eV)	HOMO (eV)	LUMO (eV)
2a	3.08	3.25; 2.45	−5.7	−2.6
2b	3.26	2.55	−5.7	−2.4
2c	3.05	2.85; 2.60	−5.0	−2.0
2d	2.82	2.30	−4.5	−1.7
2e	3.08	2.95; 2.60; 2.00	−4.7	−1.6

HOMO energy, estimated using the surface potential measurement by means of the Kelvin probe method; LUMO energy, calculated from the estimated HOMO energy and optical energy gap of the organic film deposited on the quartz substrate.

## Supplementary Materials

The following are available online: Figure S1: Dependence of the fluorescence intensity of **1a** and **1b** on the water fraction in THF, Table S1: Calculated dihedral angle values between aryl moieties of **2a**–**2e**, Figures S2–S6: Molecular structure and ground state molecular geometry of **2a**–**2e**, Figures S7–S14: Thermogravimetric curves of **1a**–**1c** and **2a**–**2e**, Figures SX1–SX12: ^1^H-NMR, ^13^C-NMR, FTIR, and HRMS spectra of 4-azafluorenones **1a**–**1c;** Figures SX13–SX43: ^1^H-NMR, ^13^C-NMR, FTIR, and HRMS spectra of α-carbolines **2a**–**2e** and Intermediates A, B, and C.

## References

[1] Mei J., Leung N.L.C., Kwok R.T.K., Lam J.W.Y., Tang B.Z. (2015). Aggregation-Induced Emission: Together We Shine, United We Soar!. Chem. Rev..

[2] Chen M., Hu X., Liu J., Li B., Leung N.L.C., Viglianti L., Cheung T.S., Sung H.H.Y., Kwok R.T.K., Williams I.D. (2018). Rational design of red AIEgens with a new core structure from non-emissive heteroaromatics. Chem. Sci..

[3] Shen P., Zhuang Z., Zhao Z., Tang B.Z. (2018). AIEgens based on main group heterocycles. J. Mater. Chem. C.

[4] Robb M.J., Li W., Gergely R.C.R., Matthews C.C., White S.R., Sottos N.R., Moore J.S. (2016). A Robust Damage-Reporting Strategy for Polymeric Materials Enabled by Aggregation-Induced Emission. ACS Cent. Sci..

[5] Winkler M., Houk K.N. (2007). Nitrogen-rich oligoacenes: Candidates for n-channel organic semiconductors. J. Am. Chem. Soc..

[6] Li G., Duong H.M., Zhang Z., Xiao J., Liu L., Zhao Y., Zhang H., Huo F., Li S., Ma J. (2012). Approaching a stable, green twisted heteroacene through “clean reaction” strategy. Chem. Commun..

[7] Wu Y., Yin Z., Xiao J., Liu Y., Wei F., Tan K.J., Kloc C., Huang L., Yan Q., Hu F. (2012). Crystal Structure and Phototransistor Behavior of N-Substituted Heptacence. ACS Appl. Mater. Interfaces.

[8] Li G., Wu Y., Gao J., Wang C., Li J., Zhang H., Zhao Y., Zhao Y., Zhang Q. (2012). Synthesis and Physical Properties of Four Hexazapentacene Derivatives. J. Am. Chem. Soc..

[9] Bunz U.H.F., Engelhart J.U., Lindner B.D., Schaffroth M. (2013). Large N-Heteroacenes: New Tricks for Very Old Dogs?. Angew. Chemie Int. Ed..

[10] Miao Q. (2012). N-Heteropentacenes and N-Heteropentacenequinones: From Molecules to Semiconductors. Synlett.

[11] Li J., Yan F., Gao J., Li P., Xiong W.-W., Zhao Y., Sun X.W., Zhang Q. (2015). Synthesis, physical properties and OLED performance of azatetracenes. Dye. Pigment..

[12] Zhang Y., Lai S.-L., Tong Q.-X., Lo M.-F., Ng T.-W., Chan M.-Y., Wen Z.-C., He J., Jeff K.-S., Tang X.-L. (2012). High Efficiency Nondoped Deep-Blue Organic Light Emitting Devices Based on Imidazole-π-triphenylamine Derivatives. Chem. Mater..

[13] Yang X., Xu X., Zhou G. (2015). Recent advances of the emitters for high performance deep-blue organic light-emitting diodes. J. Mater. Chem. C.

[14] Zhao L., Wang S., Ding J., Wang L. (2018). Solution processible distyrylarylene-based fluorescent dendrimers: Tuning of carbazole-dendron generation leads to nondoped deep-blue electroluminescence. Org. Electron..

[15] Liu H., Bai Q., Li W., Guo Y., Yao L., Gao Y., Li J., Lu P., Yang B., Ma Y. (2016). Efficient deep-blue non-doped organic light-emitting diode with improved roll-off of efficiency based on hybrid local and charge-transfer excited state. RSC Adv..

[16] Wang Z., Li X., Xue K., Li H., Zhang X., Liu Y., Yu Z., Lu P., Chen P. (2016). Towards stable deep-blue emission and low efficiency roll-off in OLEDs based on phenanthroimidazole dimers. J. Mater. Chem. C.

[17] Chen W.-C., Yuan Y., Wu G.-F., Wei H.-X., Tang L., Tong Q.-X., Wong F.-L., Lee C.-S. (2014). Staggered Face-to-Face Molecular Stacking as a Strategy for Designing Deep-Blue Electroluminescent Materials with High Carrier Mobility. Adv. Opt. Mater..

[18] Sun W., Zhou N., Xiao Y., Wang S., Li X. (2018). Novel carbazolyl-substituted spiro[acridine-9,9′-fluorene] derivatives as deep-blue emitting materials for OLED applications. Dye. Pigment..

[19] Mucur S.P., Kök C., Bilgili H., Canımkurbey B., Koyuncu S. (2018). Conventional and inverted organic light emitting diodes based on bright green emmisive polyfluorene derivatives. Polymer (Guildf)..

[20] Gan L., Li X., Cai X., Liu K., Li W., Su S.-J. (2018). D–A–D-type orange-light emitting thermally activated delayed fluorescence (TADF) materials based on a fluorenone unit: simulation, photoluminescence and electroluminescence studies. Beilstein J. Org. Chem..

[21] Ni F., Zhu Z., Tong X., Xie M., Zhao Q., Zhong C., Zou Y., Yang C. (2018). Organic emitter integrating aggregation-induced delayed fluorescence and room-temperature phosphorescence characteristics, and its application in time-resolved luminescence imaging. Chem. Sci..

[22] Huang W., Einzinger M., Zhu T., Chae H.S., Jeon S., Ihn S.-G., Sim M., Kim S., Su M., Teverovskiy G. (2018). Molecular Design of Deep Blue Thermally Activated Delayed Fluorescence Materials Employing a Homoconjugative Triptycene Scaffold and Dihedral Angle Tuning. Chem. Mater..

[23] Zhang Y.-X., Yuan Y., Wang Q., Hu Y., Khan A., Jiang Z.-Q., Liao L.-S. (2018). Highly efficient non-doped deep-blue organic light-emitting diodes by employing a highly rigid skeleton. Dye. Pigment..

[24] Liu X.-Y., Zhang Y.-J., Fei X., Ran Q., Fung M.-K., Fan J. (2019). Diazaspirocycles: novel platforms for efficient phosphorescent organic light-emitting diodes. J. Mater. Chem. C.

[25] Kim S.J., Kim Y.J., Son Y.H., Hur J.A., Um H.A., Shin J., Lee T.W., Cho M.J., Kim J.K., Joo S. (2013). High-efficiency blue phosphorescent organic light-emitting diodes using a carbazole and carboline-based host material. Chem. Commun..

[26] Lee C.W., Im Y., Seo J.-A., Lee J.Y. (2013). Carboline derivatives with an ortho-linked terphenyl core for high quantum efficiency in blue phosphorescent organic light-emitting diodes. Chem. Commun..

[27] Chang C.-W., Sølling T.I., Diau E.W.-G. (2017). Revisiting the photophysics of 9-fluorenone: Ultrafast time-resolved fluorescence and theoretical studies. Chem. Phys. Lett..

[28] Ghosh I., Mukhopadhyay A., Koner A.L., Samanta S., Nau W.M., Moorthy J.N. (2014). Excited-state properties of fluorenones: influence of substituents, solvent and macrocyclic encapsulation. Phys. Chem. Chem. Phys..

[29] Breton G.W., Vang X. (1998). Photodimerization of Anthracene. J. Chem. Educ..

[30] Becker H.-D., Andersson K. (1984). Photochemical Diels—Alder dimerization of 9-phenylethylnylanthracene. J. Photochem..

[31] Tu M., Reinsch H., Rodríguez-Hermida S., Verbeke R., Stassin T., Egger W., Dickmann M., Dieu B., Hofkens J., Vankelecom I. (2018). Reversible Optical Writing and Data Storage in an Anthracene-Loaded Metal-Organic Framework. Angew. Chemie.

[32] Zdobinsky T., Sankar Maiti P., Klajn R. (2014). Support Curvature and Conformational Freedom Control Chemical Reactivity of Immobilized Species. J. Am. Chem. Soc..

[33] Kim M., Hohman J.N., Cao Y., Houk K.N., Ma H., Jen A.K.-Y., Weiss P.S. (2011). Creating favorable geometries for directing organic photoreactions in alkanethiolate monolayers. Science.

[34] Moustafa A.H., Kaddah A.M., El-Abbady S.A., Gado S.H. (1982). Cyclocondensation of Cyanoacetamide and N-Substituted Cyanoacetamides with 2-Arylmethylene indan-1-one and -indan-1,3-diones. J. Prakt. Chemie.

[35] Shrestha A., Park S., Jang H.J., Katila P., Shrestha R., Kwon Y., Lee E.-S. (2018). A new phenolic series of indenopyridinone as topoisomerase inhibitors: Design, synthesis, and structure-activity relationships. Bioorg. Med. Chem..

[36] Chen G., Wang Z., Zhang X., Fan X. (2017). Synthesis of Functionalized Pyridines via Cu(II)-Catalyzed One-Pot Cascade Reactions of Inactivated Saturated Ketones with Electron-Deficient Enamines. J. Org. Chem..

[37] Hostyn S., Van Baelen G., Lemière G.L.F., Maes B.U.W. (2008). Synthesis of α-Carbolines Starting from 2,3-Dichloropyridines and Substituted Anilines. Adv. Synth. Catal..

