# Supplementary material for: 4-Azafluorenone and α-Carboline Fluorophores with Green and Violet/Blue Emission
Source: Molecules. 2019 Jun 27;24(13):2378. doi: 10.3390/molecules24132378 (PMC6651119; doi:10.3390/molecules24132378)
Supplement: Supplementary file 1 [file molecules-24-02378-s001.pdf]

Electronic Supplementary Information (ESI)

4-Azafluorenone and α-carboline fluorophores with green and violet/blue emission

Marek Cigáň [1,*], Peter Danko [2], Henrich Brath [2], Matúš Čakurda [2], Roman Fišera [2], Jana Donovalová [1], Juraj Filo [1], Martin Weis [3], Ján Jakabovič [3], Miroslav Novota [3] and Anton Gáplovský [1]

[1] Faculty of Natural Sciences, Institute of Chemistry, Comenius University, Ilkovičova 6, Mlynská dolina CH-2, SK-842 15 Bratislava, Slovakia; E-Mails: marek.cigan@uniba.sk (M.C.); jana.donovalova@uniba.sk (J.D.); juraj.filo@uniba.sk (J.F.); anton.gaplovsky@uniba.sk (A.G.)

[2] SYNKOLA, Ltd, Ilkovičova 6, 842 15, Bratislava, Slovakia; E-Mails: danko@synkola.sk (P.D.); brath@synkola.sk (H.B.); cakurda@synkola.sk (M.Č.); fisera@synkola.sk (R.F.)

[3] Institute of Electronics and Photonics, Slovak University of Technology, Ilkovičova 3, SK-81219 Bratislava, Slovakia; E-Mails: martin.weis@stuba.sk (M.W.); jan.jakabovic@stuba.sk (J.J.); miroslav.novota@stuba.sk (M.N.)

* Correspondence: marek.cigan@uniba.sk

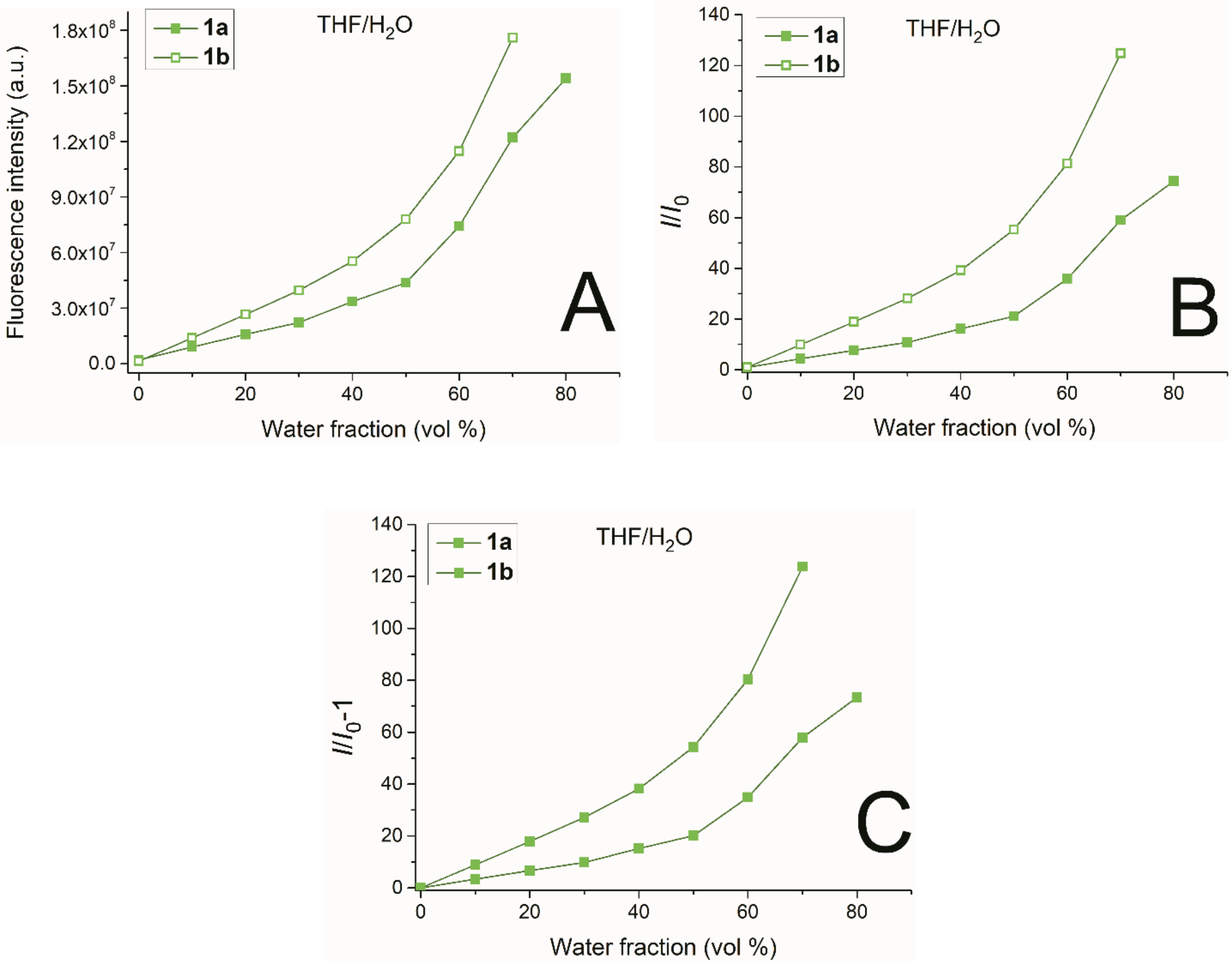

Figure S1. Plots of fluorescence intensity vs water fractions (A), I/I_0 vs water fractions (B) and I/I_0-1 vs water fractions (C) of **1a** and **1b** in THF/water mixtures; I_0 and I are the fluorescence intensities in THF solution and a THF/water mixture, respectively.

Table S1. Calculated dihedral angle values θ between aryl moieties (geometries were optimized at the B3LYP/G-311G* level of theory).

Compd	θ_1	θ_2	θ_3
**2a**	86°	40°	20°
**2b**	55°	39°	49°
**2c**	52°	88°	89°
**2d**	53°	16°	
**2e**	52°	41°	50°

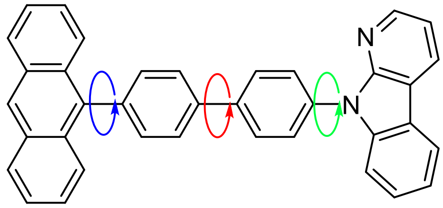

2a

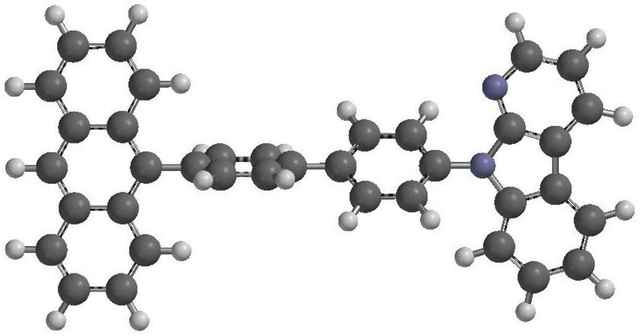

Figure S2. Molecular structure (top) and ground state molecular geometry (bottom) of **2a** (geometry was optimized at the B3LYP/G-311G* level of theory).

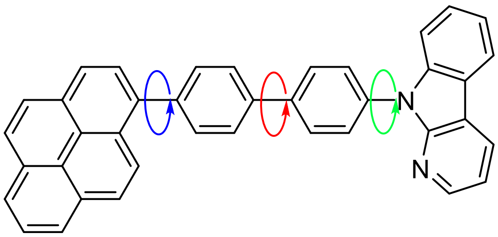

2b

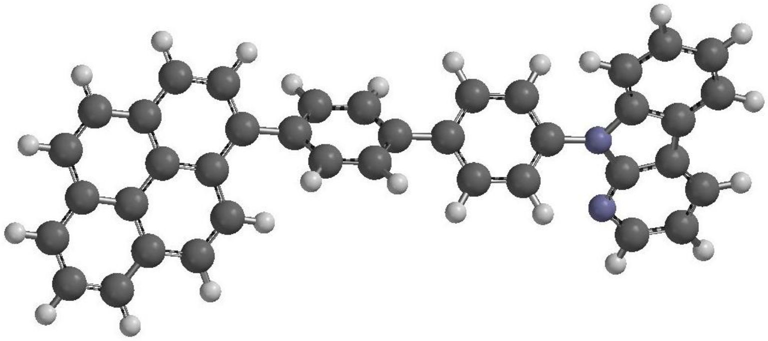

Figure S3. Molecular structure (top) and ground state molecular geometry (bottom) of **2b** (geometry was optimized at the B3LYP/G-311G* level of theory).

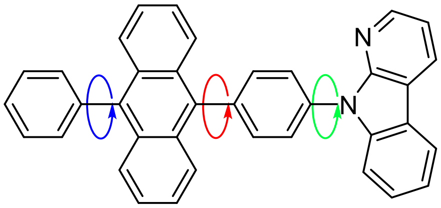

2c

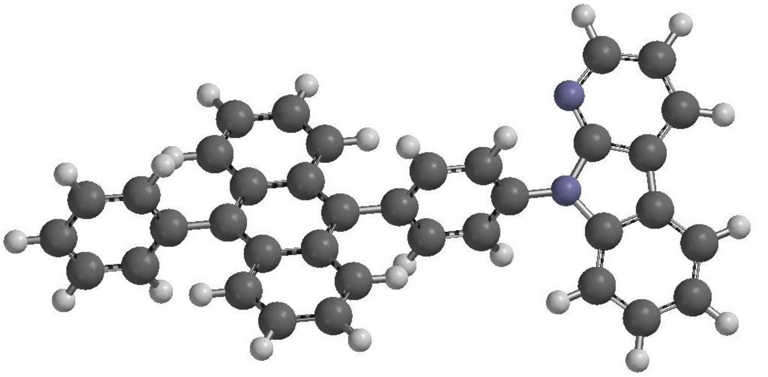

Figure S4. Molecular structure (top) and ground state molecular geometry (bottom) of **2c** (geometry was optimized at the B3LYP/G-311G* level of theory).

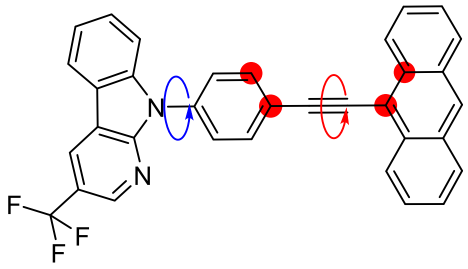

2d

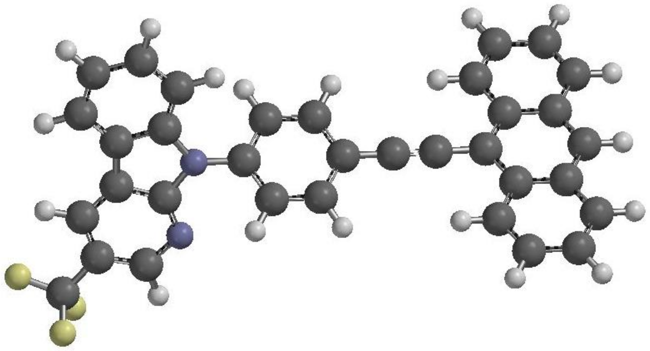

Figure S5. Molecular structure (top) and ground state molecular geometry (bottom) of **2d** (geometry was optimized at the B3LYP/G-311G* level of theory).

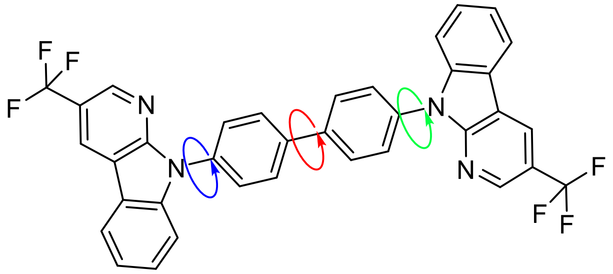

2e

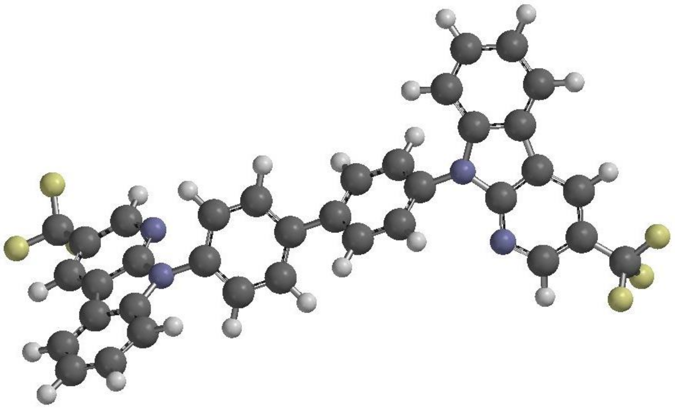

Figure S6. Molecular structure (top) and ground state molecular geometry (bottom) of **2e** (geometry was optimized at the B3LYP/G-311G* level of theory).

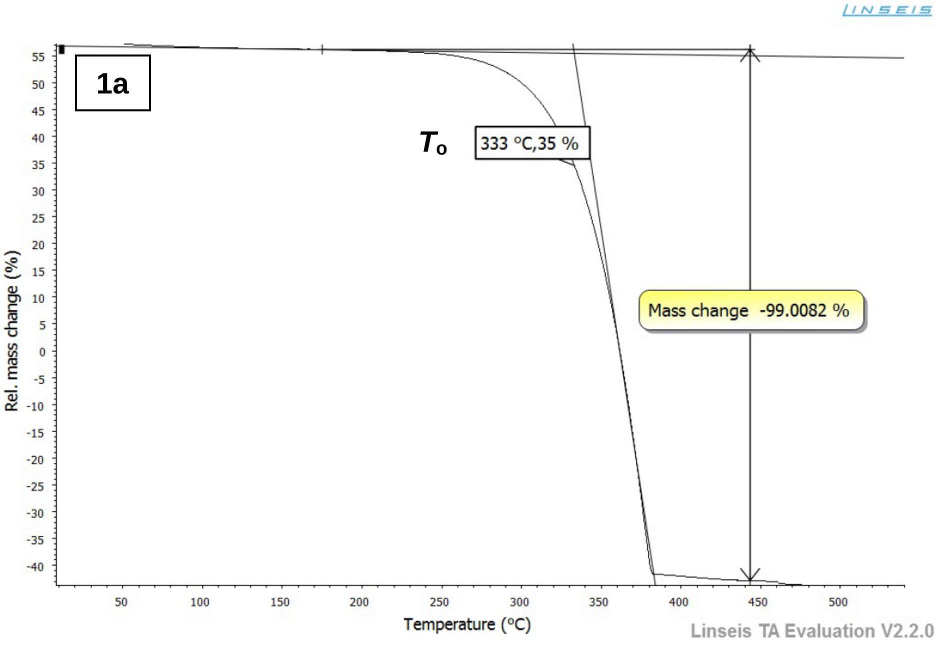

Figure S7. Thermogravimetric curve for **1a** (the thermal stability was determined from the extrapolated onset temperature T_o that denotes the temperature at which the weight loss begins).

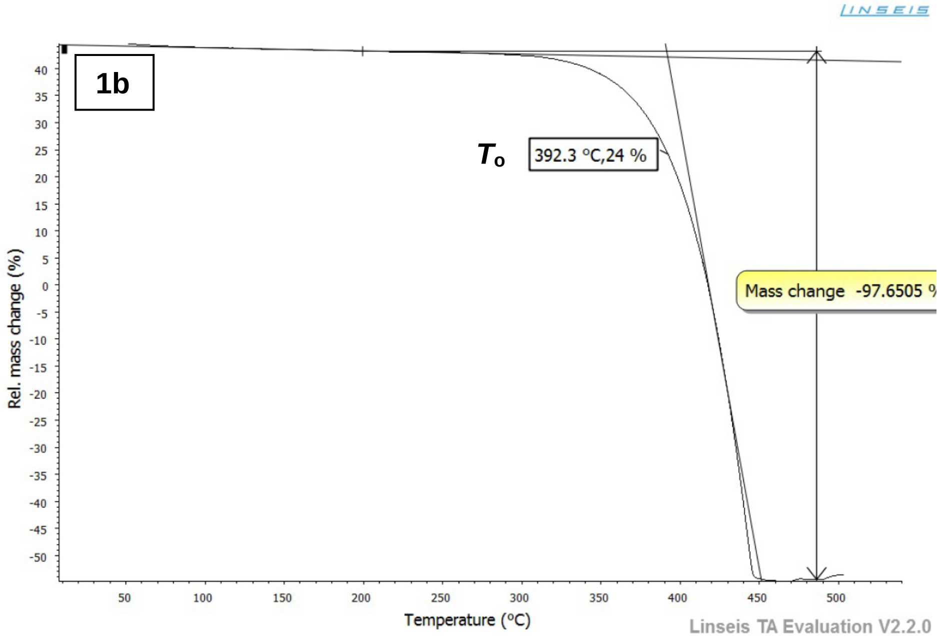

Figure S8. Thermogravimetric curve for **1b** (the thermal stability was determined from the extrapolated onset temperature T_o that denotes the temperature at which the weight loss begins).

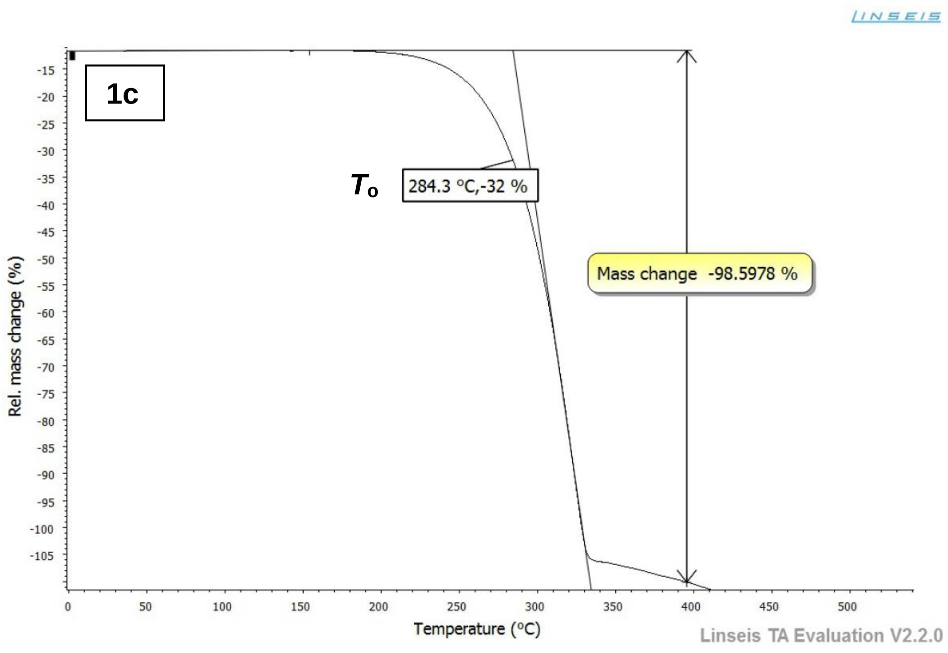

Figure S9. Thermogravimetric curve for **1c** (the thermal stability was determined from the extrapolated onset temperature T_o that denotes the temperature at which the weight loss begins).

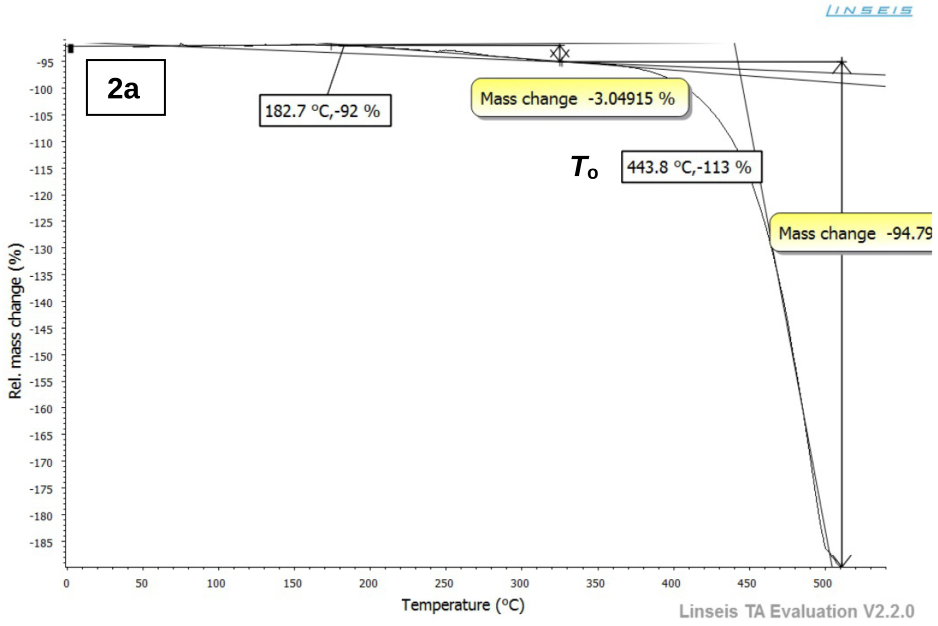

Figure S10. Thermogravimetric curve for **2a** (the thermal stability was determined from the extrapolated onset temperature T_o that denotes the temperature at which the weight loss begins).

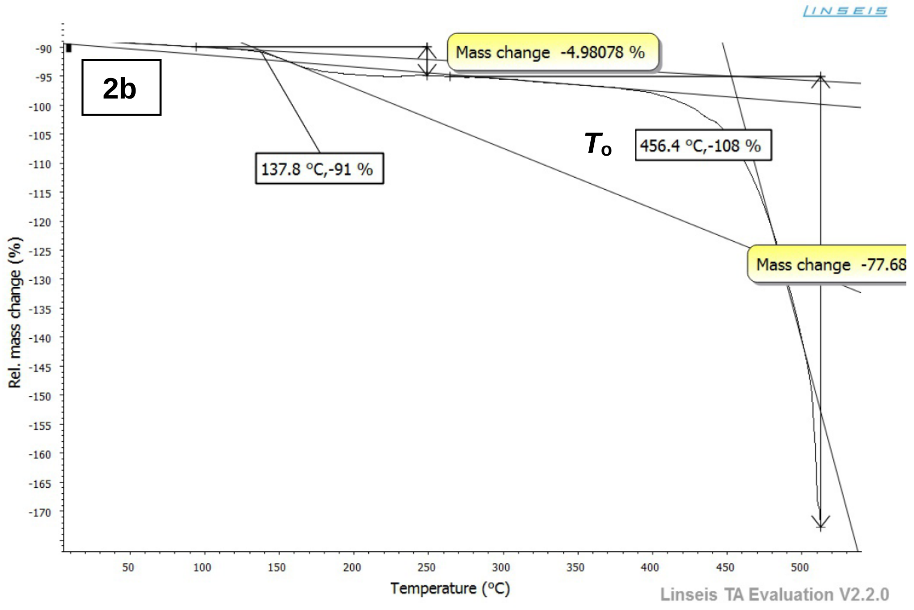

Figure S11. Thermogravimetric curve for **2b** (the thermal stability was determined from the extrapolated onset temperature T_o that denotes the temperature at which the weight loss begins).

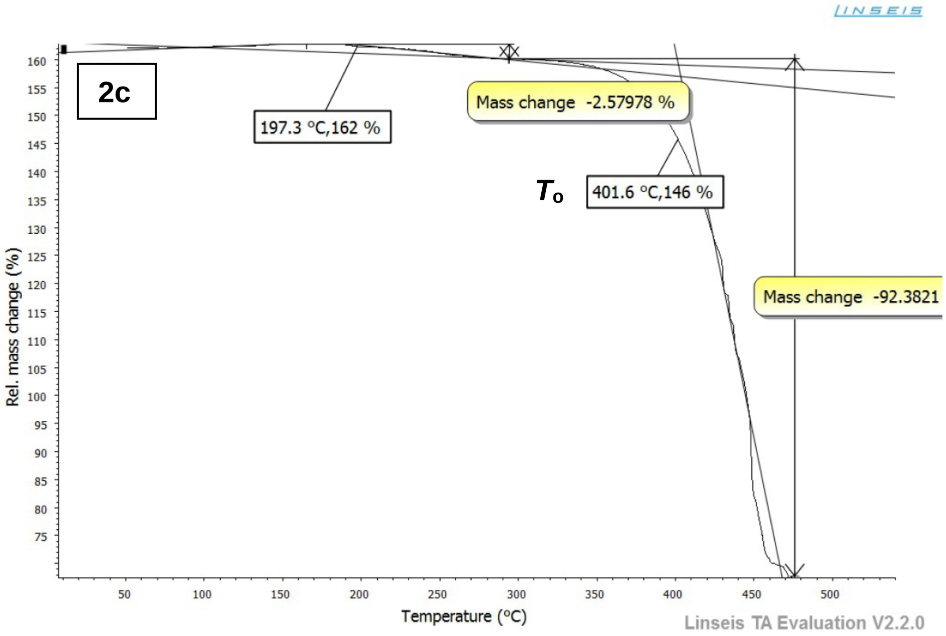

Figure S12. Thermogravimetric curve for **2c** (the thermal stability was determined from the extrapolated onset temperature T_o that denotes the temperature at which the weight loss begins).

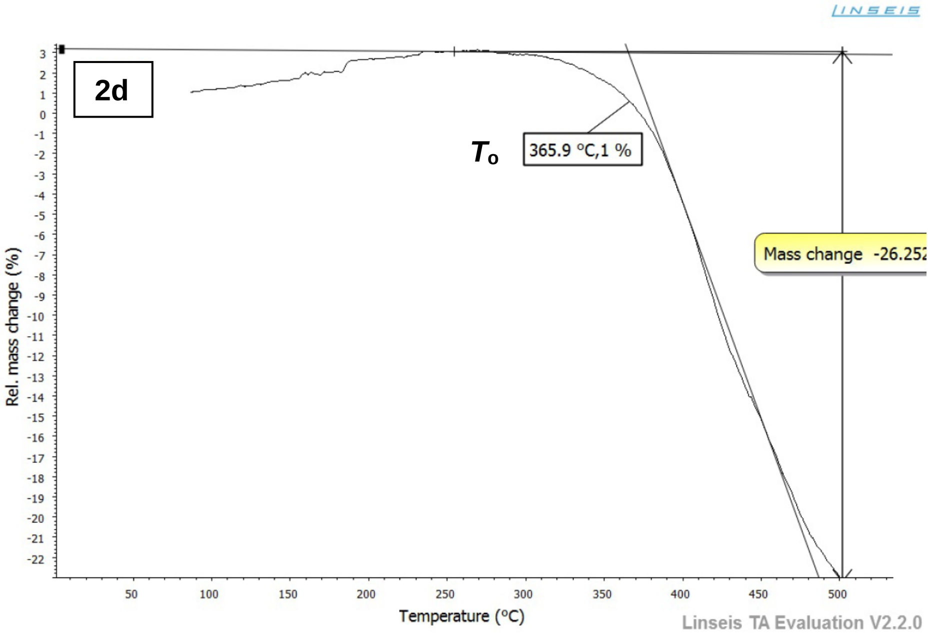

Figure S13. Thermogravimetric curve for **2d** (the thermal stability was determined from the extrapolated onset temperature T_o that denotes the temperature at which the weight loss begins).

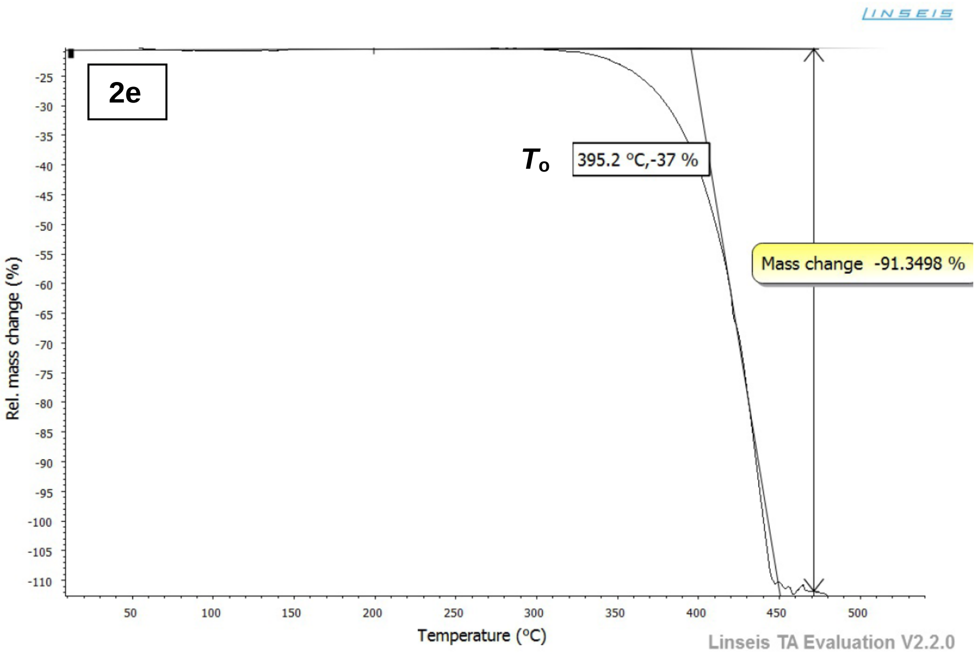

Figure S14. Thermogravimetric curve for **2e** (the thermal stability was determined from the extrapolated onset temperature T_o that denotes the temperature at which the weight loss begins).

4-Azafluorenones:

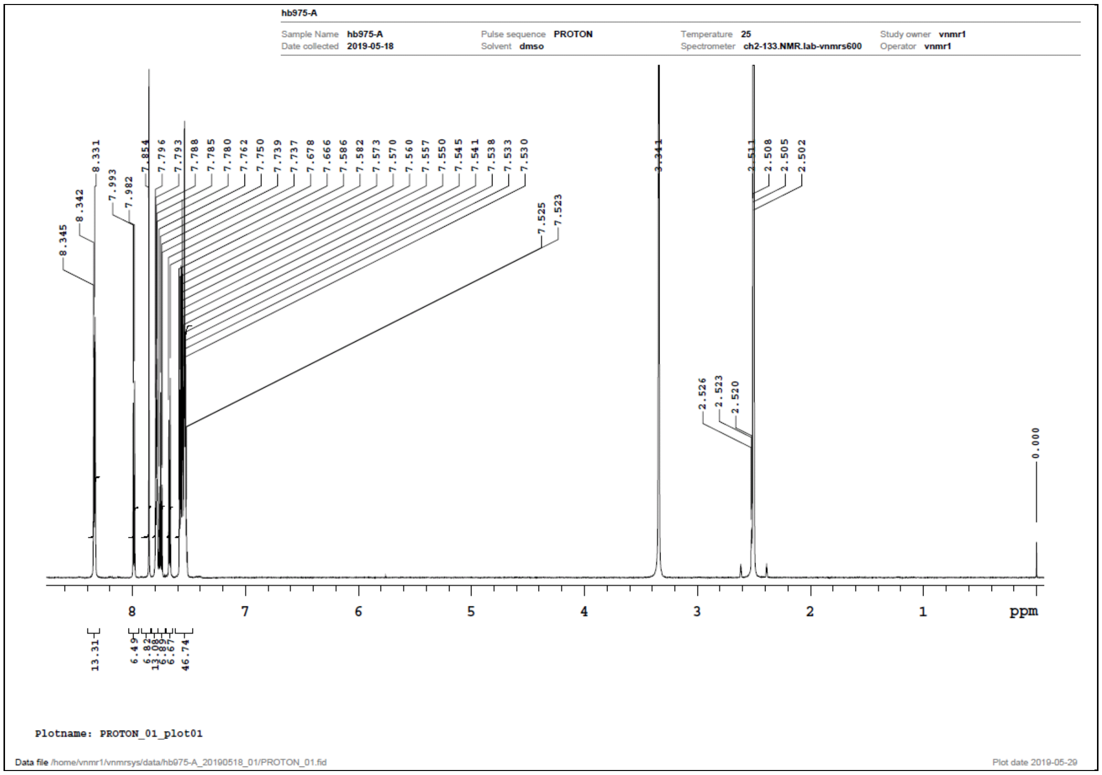

Figure SX1. ^{1}H NMR spectrum of **1a**.

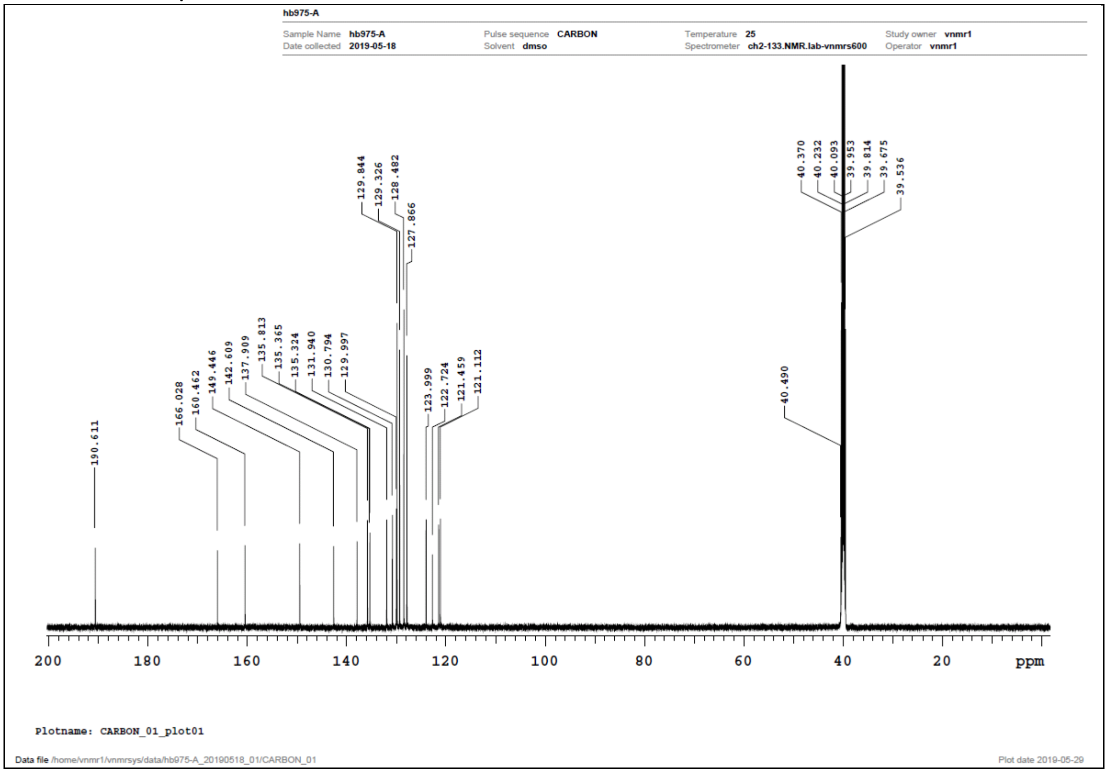

Figure SX2. ^{13}C NMR spectrum of **1a**.

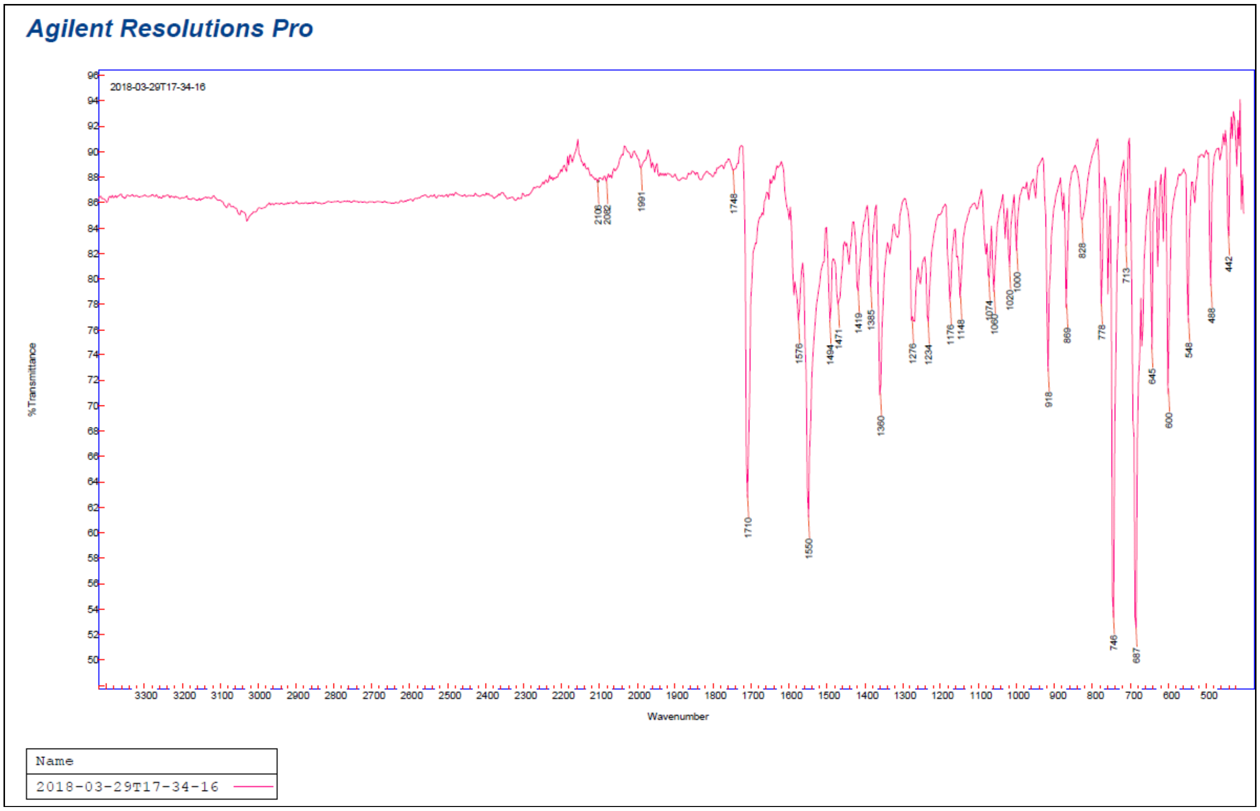

Figure SX3. FTIR spectrum of **1a** (measured by ATR technique).

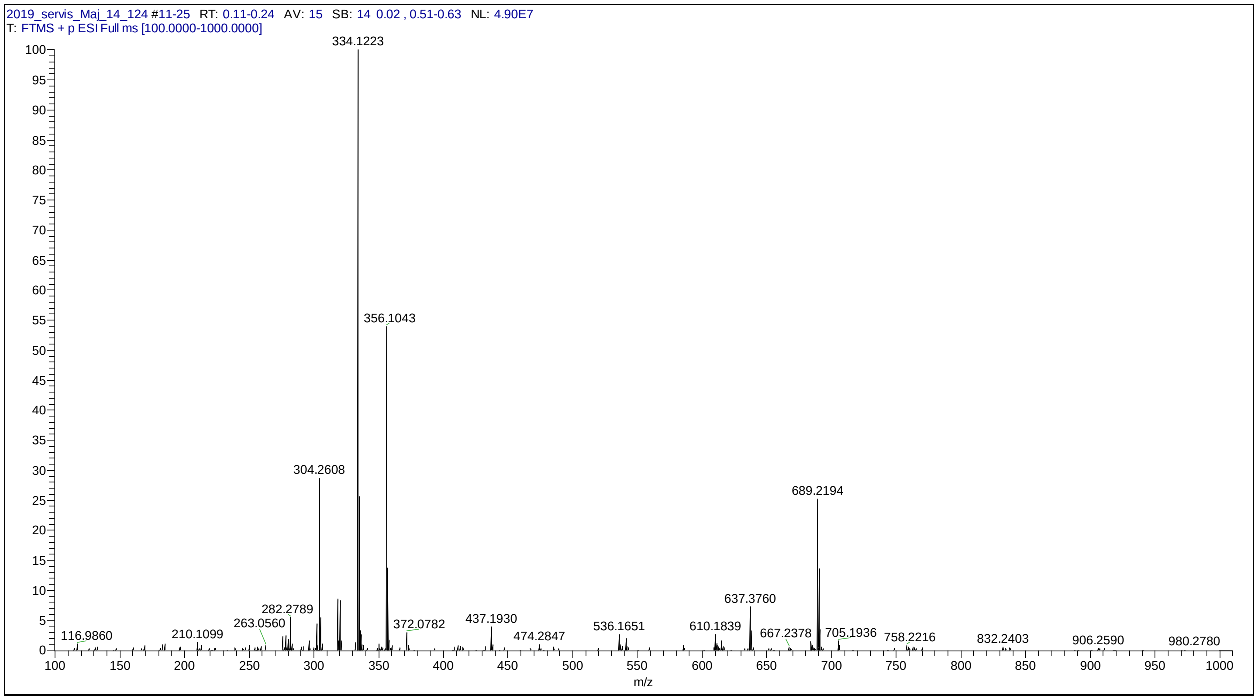

Figure SX4. Mass spectrum of **1a** in MeOH (HRMS, Thermo Scientific Orbitrap Fusion, Positive ionisation mode, Full Scan).

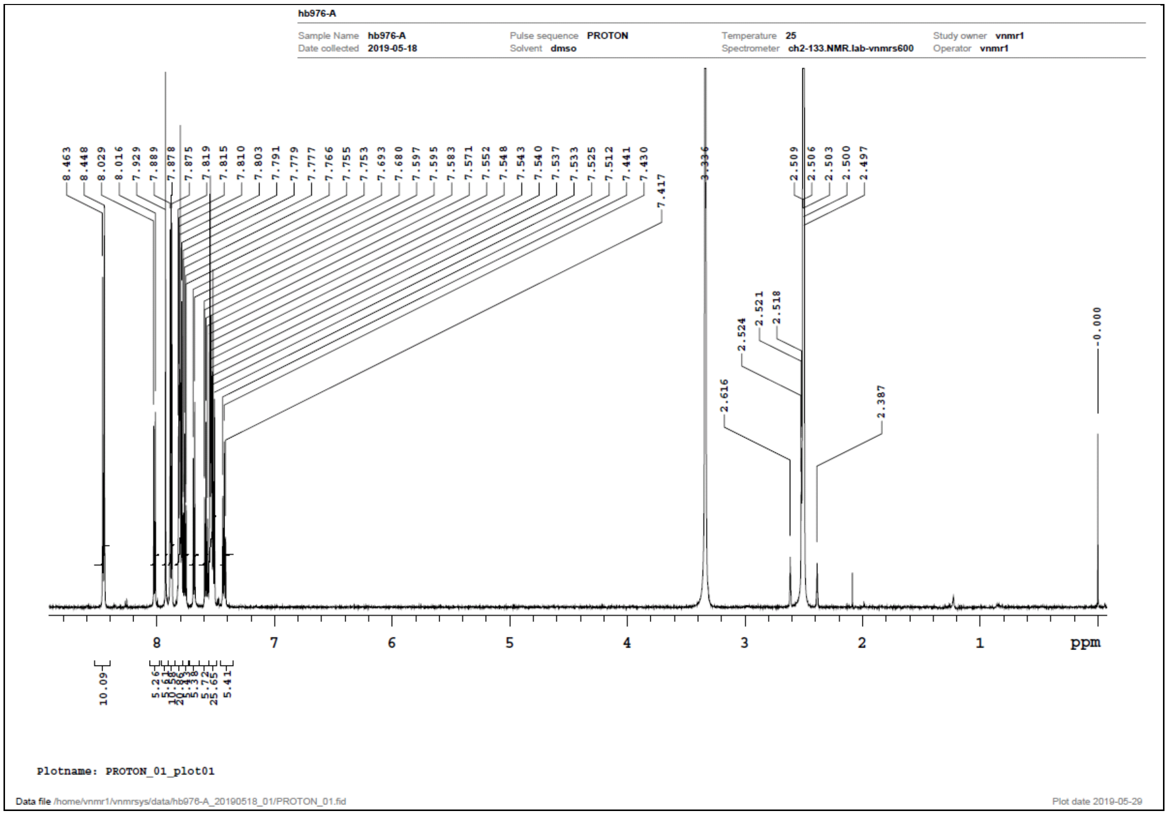

Figure SX5. ^{1}H NMR spectrum of **1b**.

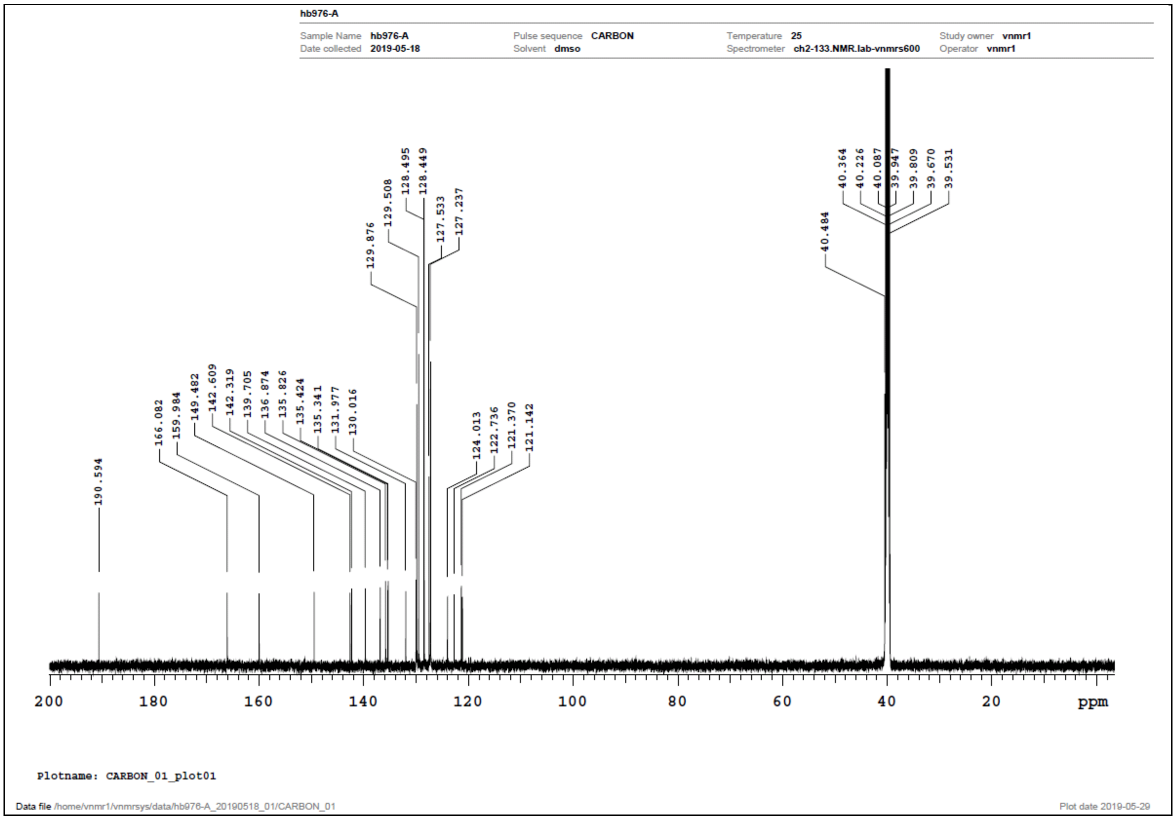

Figure SX6. ^{13}C NMR spectrum of **1b**.

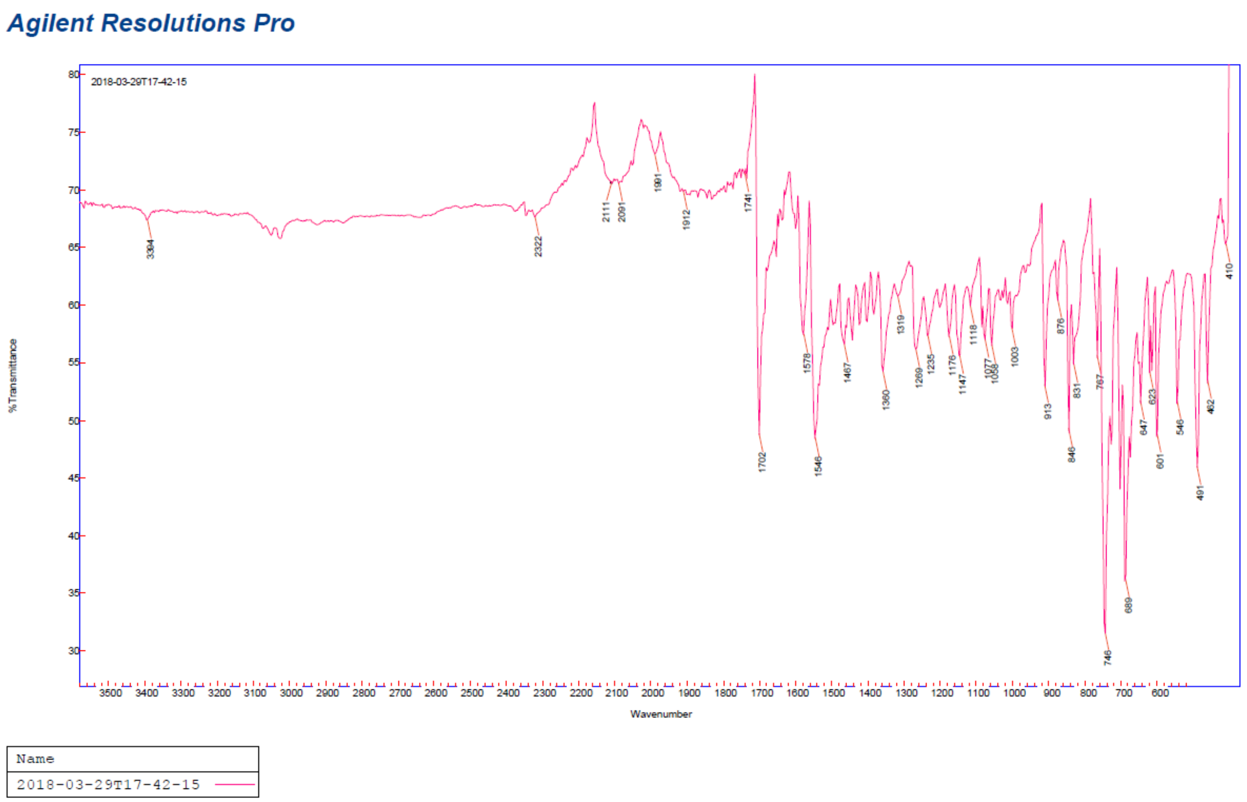

Figure SX7. FTIR spectrum of **1b** (measured by ATR technique).

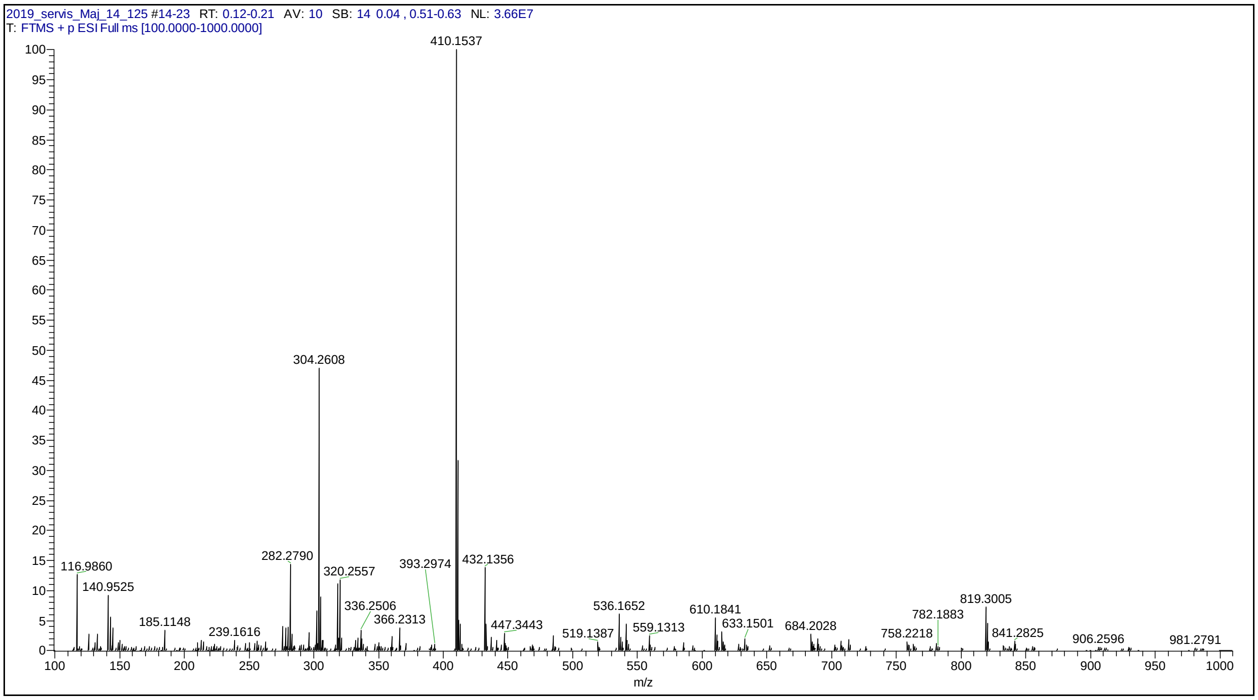

Figure SX8. Mass spectrum of **1b** in MeOH (HRMS, Thermo Scientific Orbitrap Fusion, Positive ionisation mode, Full Scan).

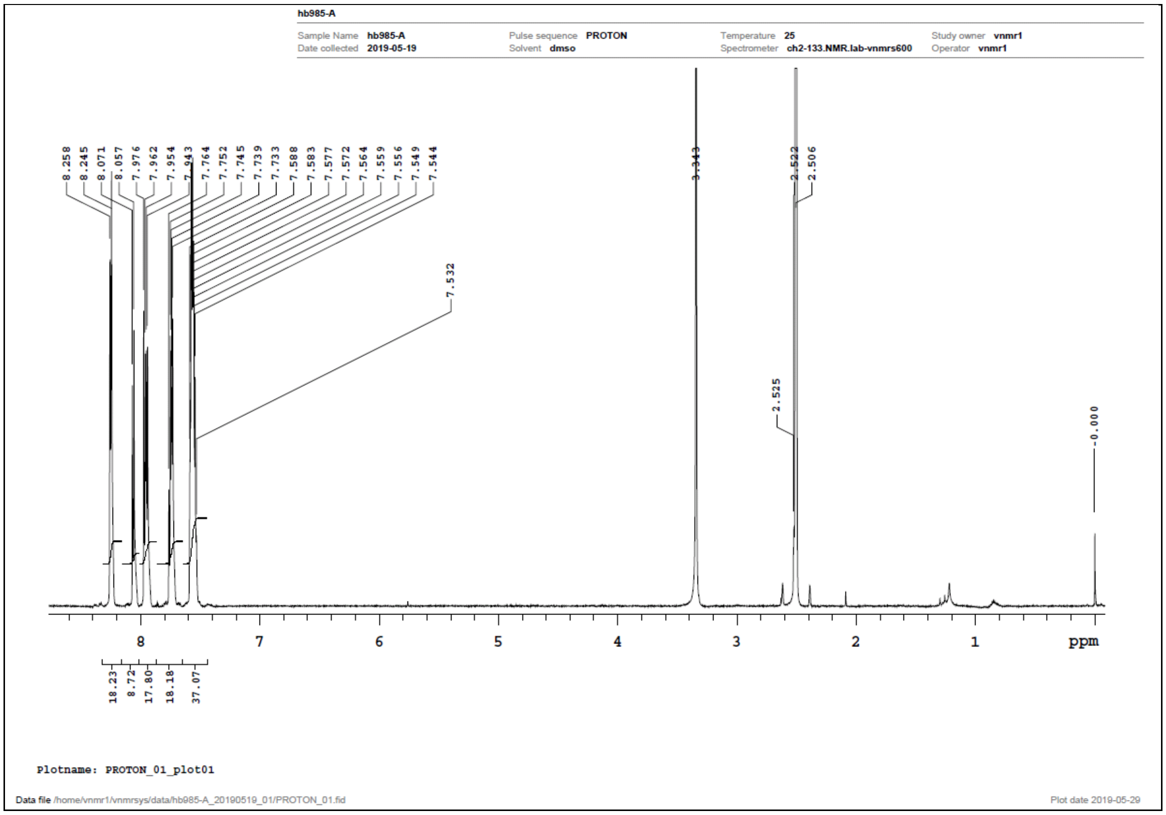

Figure SX9. ^{1}H NMR spectrum of **1c**.

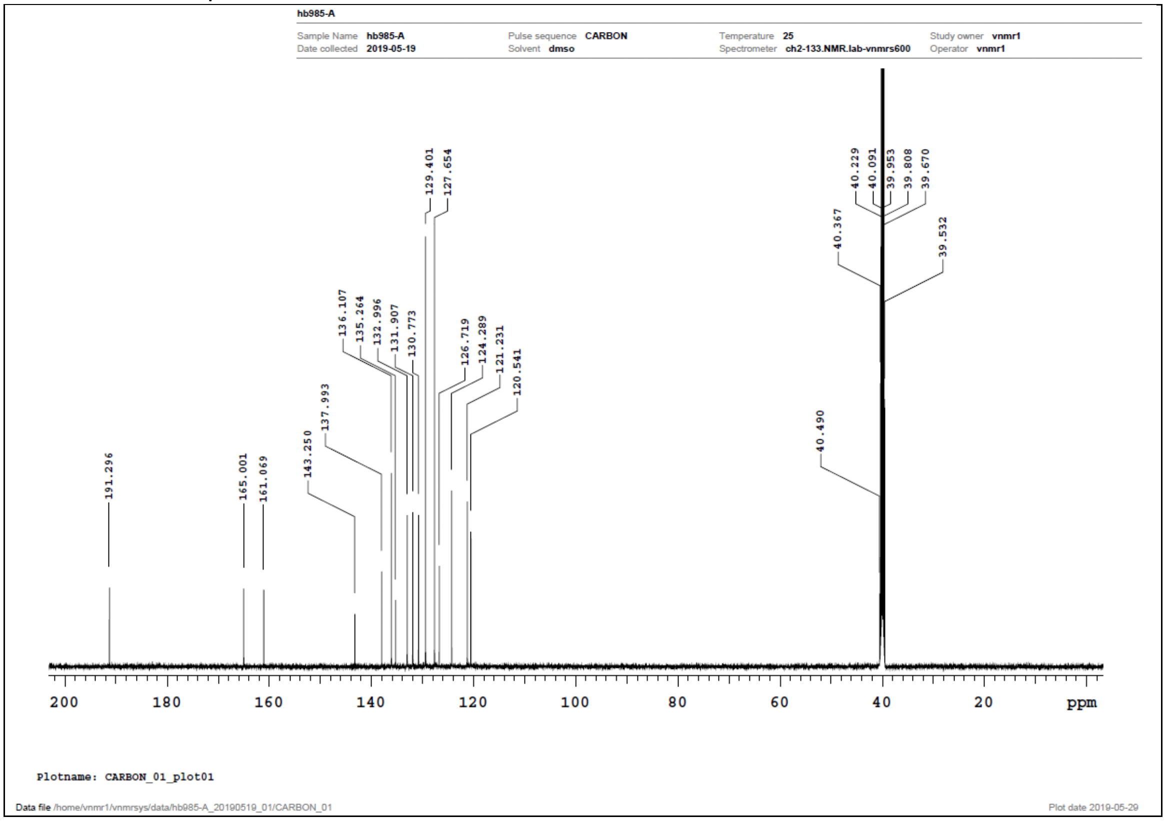

Figure SX10. ^{13}C NMR spectrum of **1c**.

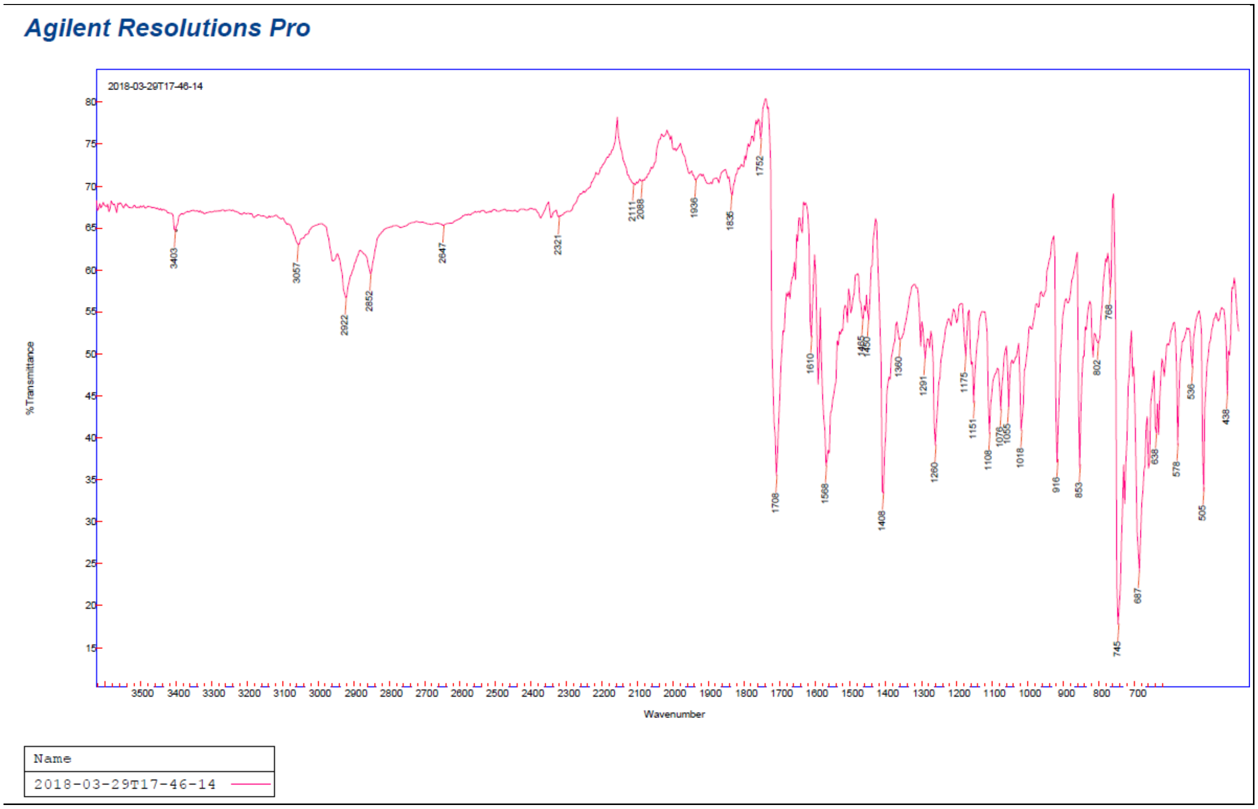

Figure SX11. FTIR spectrum of **1c** (measured by ATR technique).

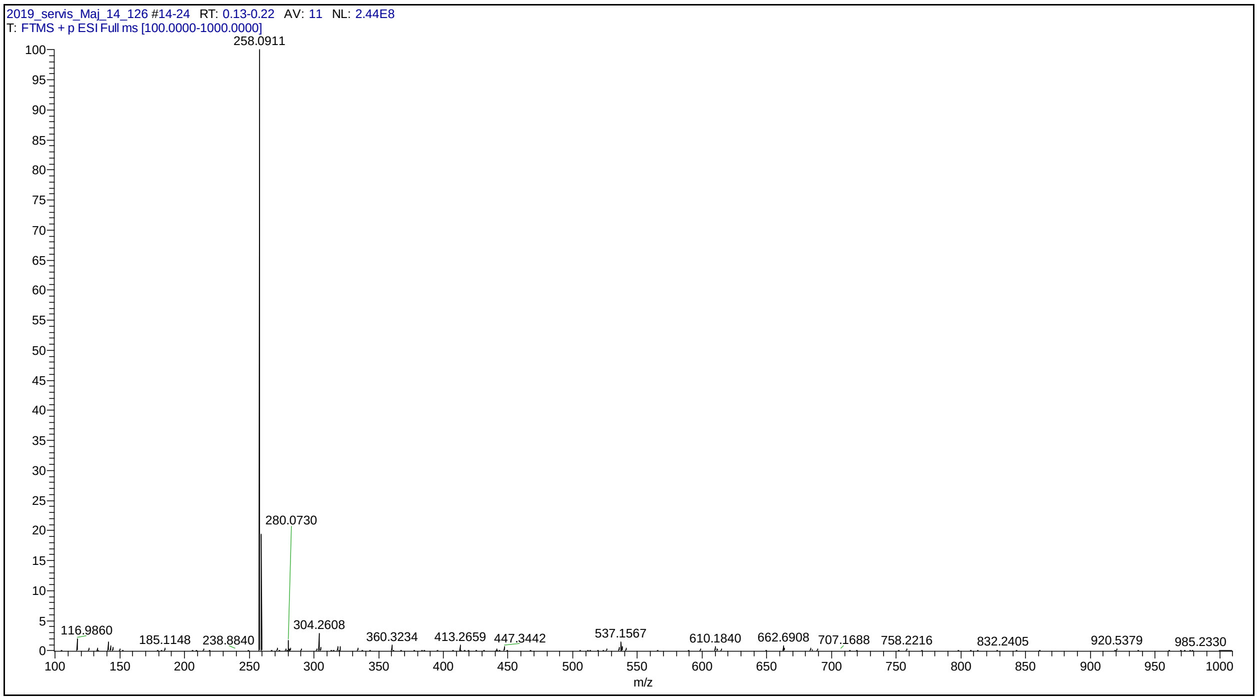

Figure SX12. Mass spectrum of **1c** in MeOH (HRMS, Thermo Scientific Orbitrap Fusion, Positive ionisation mode, Full Scan).

α-Carbolines:

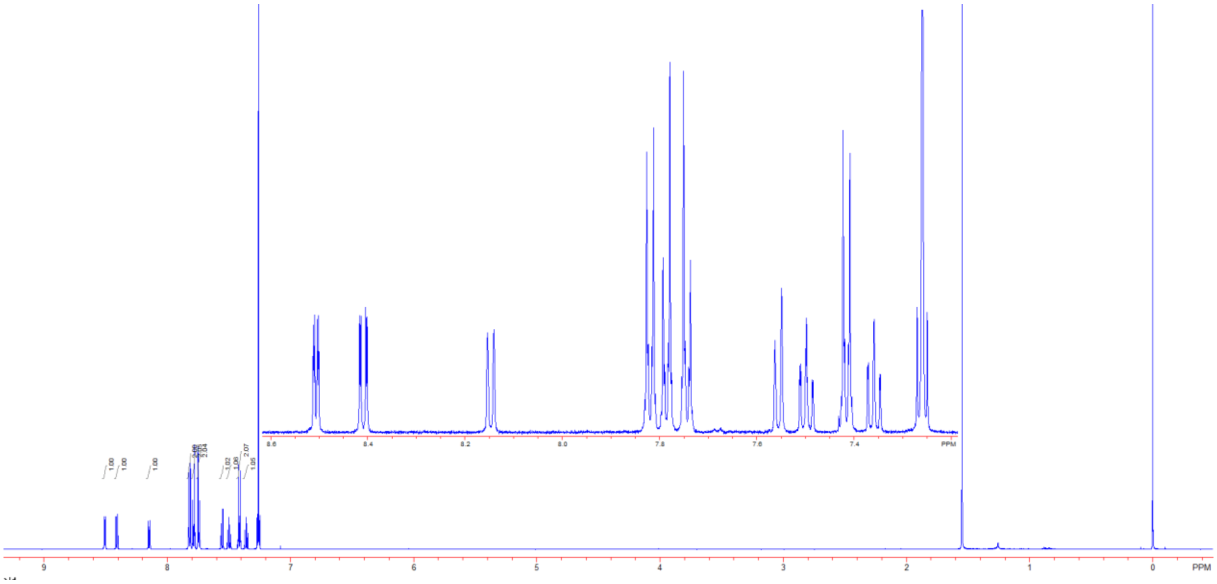

Figure SX13. ^{1}H NMR spectrum of intermediate A in $CDCl_3$.

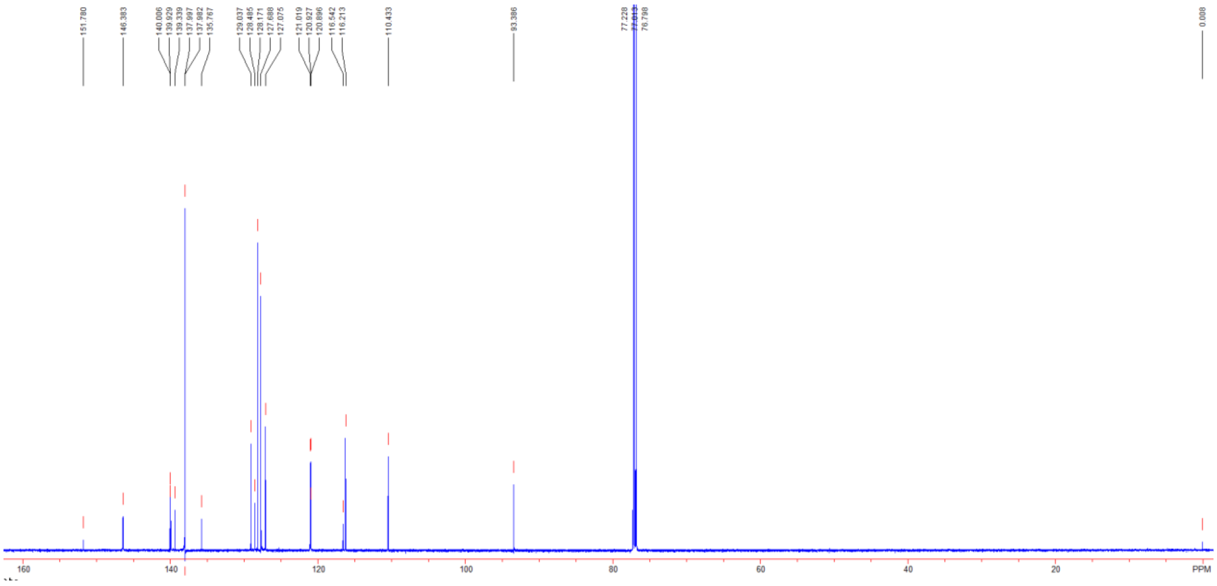

Figure SX14. ^{13}C NMR spectrum of intermediate A in $CDCl_3$.

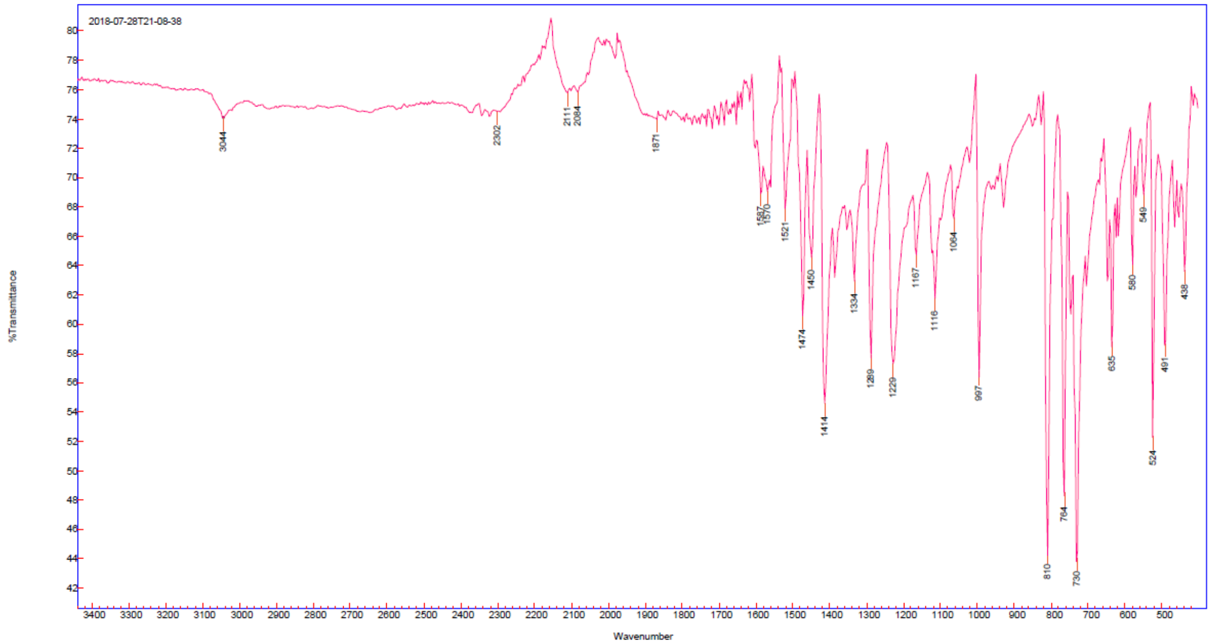

Figure SX15. FTIR spectrum of intermediate A (measured by ATR technique).

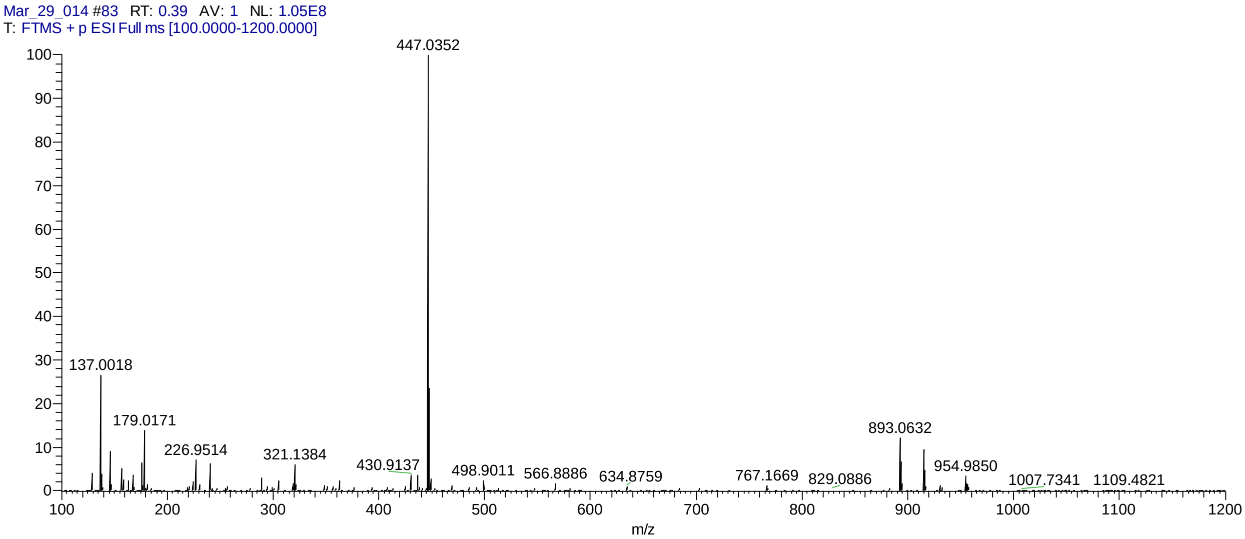

Figure SX16. Mass spectrum of intermediate A in MeOH (HRMS, Thermo Scientific Orbitrap Fusion, Positive ionisation mode, Full Scan).

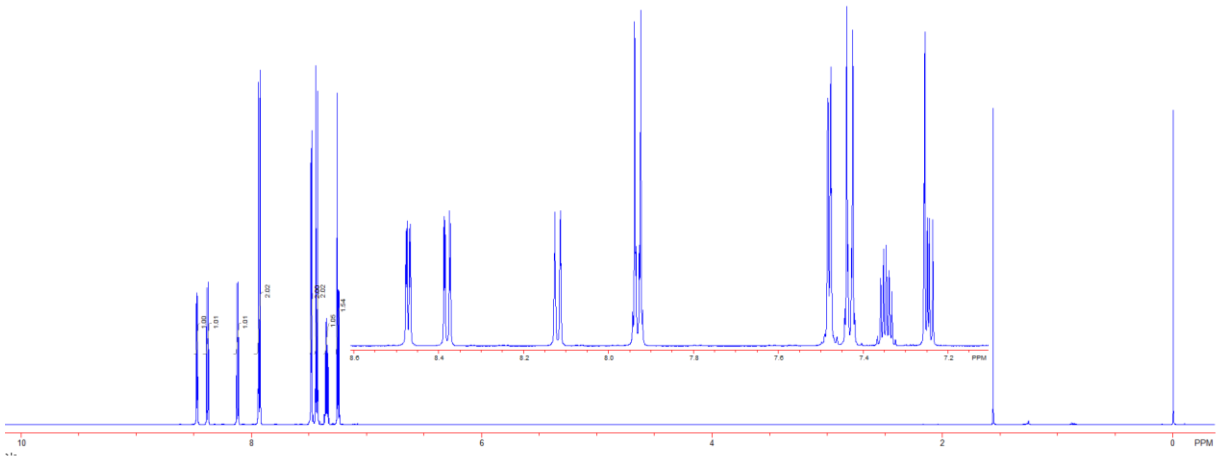

Figure SX17. ^{1}H NMR spectrum of intermediate B in $CDCl_3$.

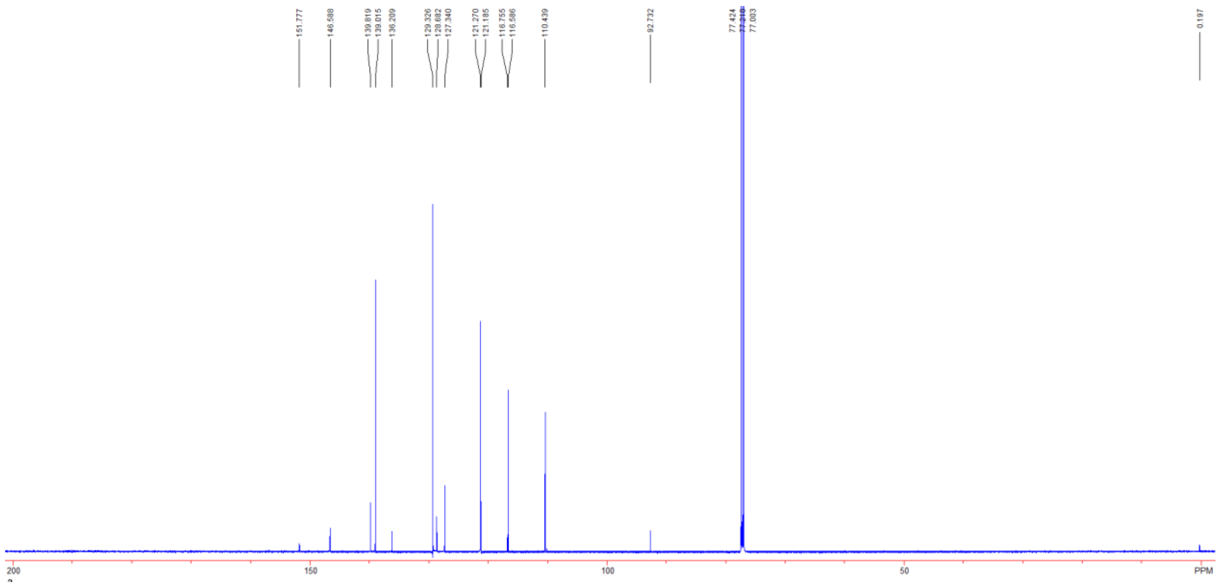

Figure SX18. ^{13}C NMR spectrum of intermediate B in $CDCl_3$.

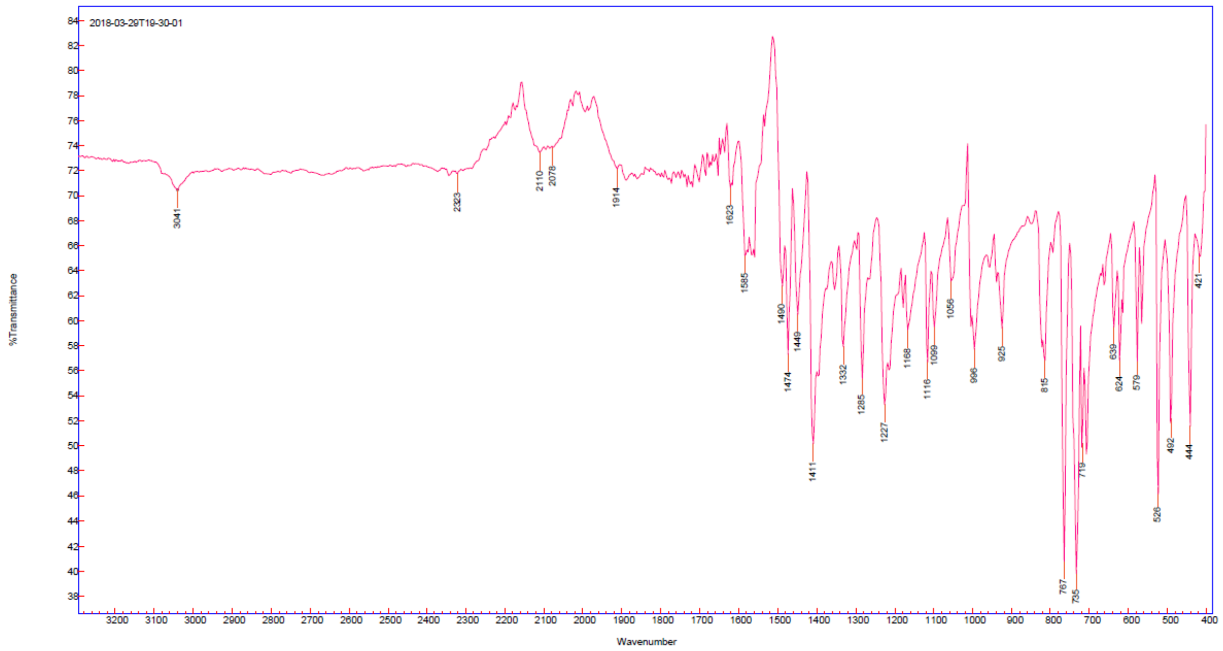

Figure SX19. FTIR spectrum of intermediate B (measured by ATR technique).

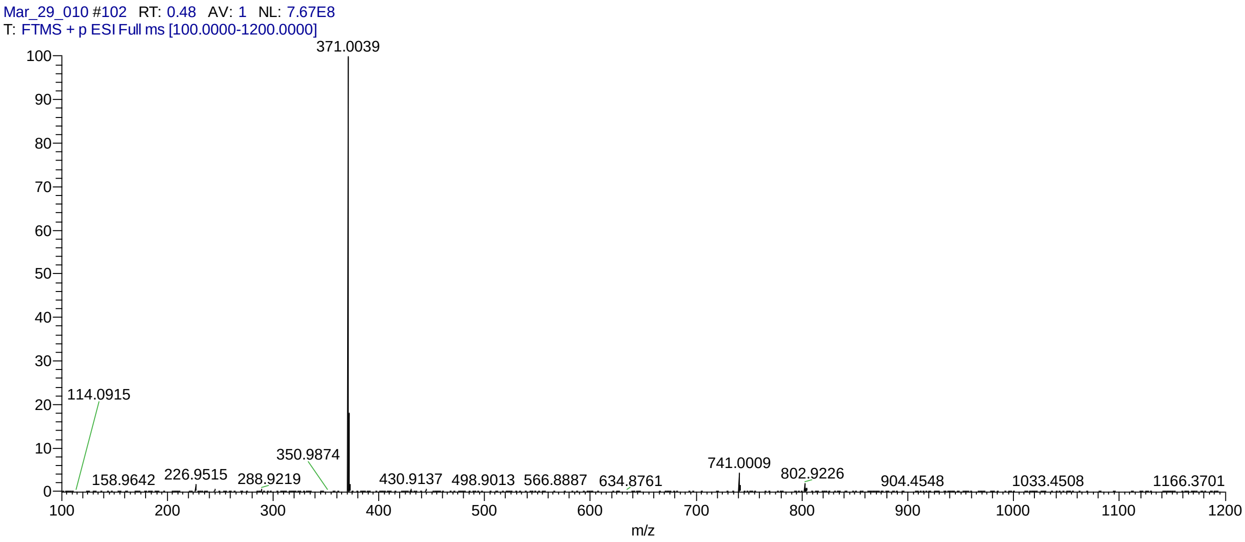

Figure SX20. Mass spectrum of intermediate B in MeOH (HRMS, Thermo Scientific Orbitrap Fusion, Positive ionisation mode, Full Scan).

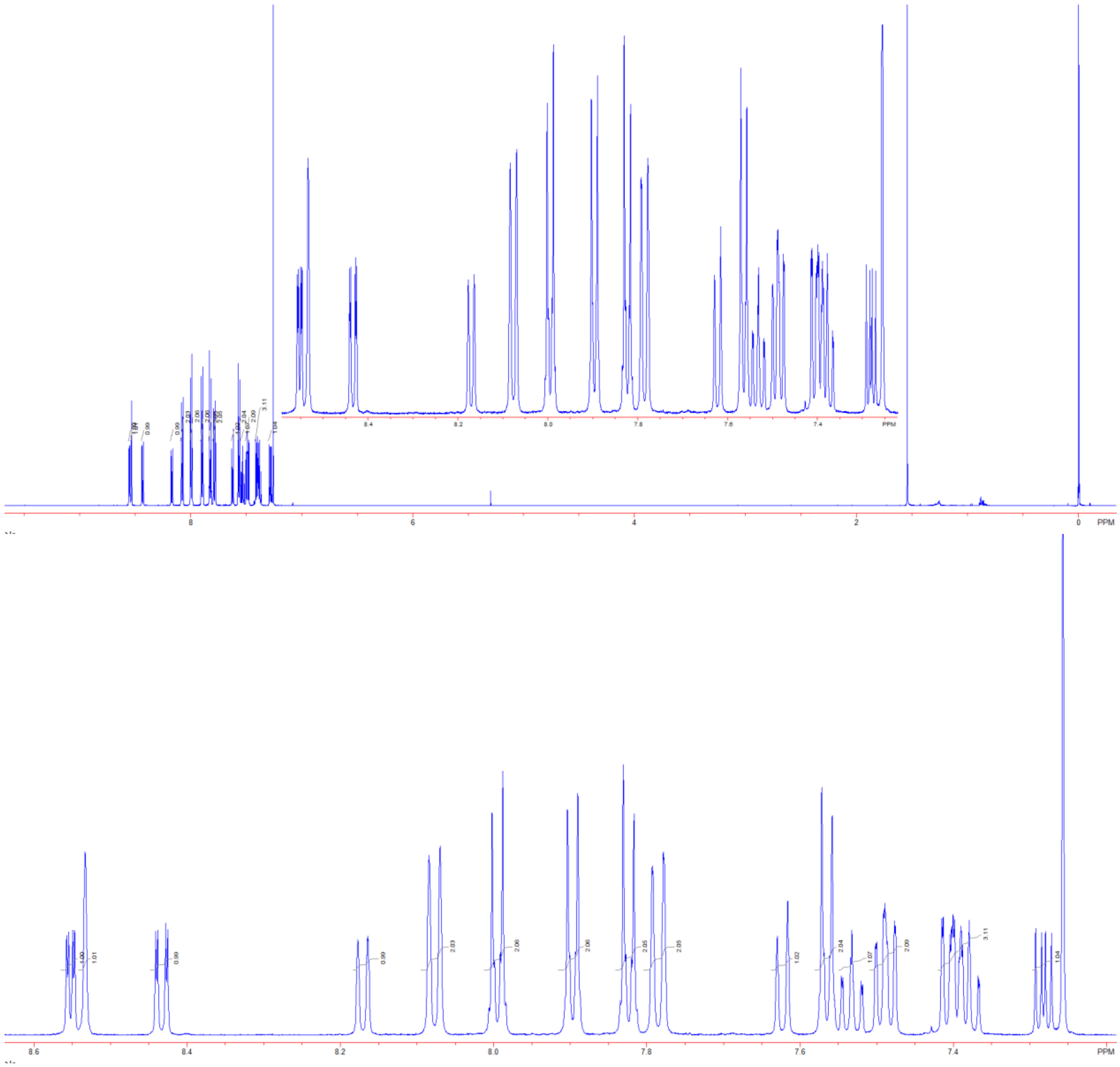

Figure SX21. ^{1}H NMR spectrum of **2a** in $CDCl_3$.

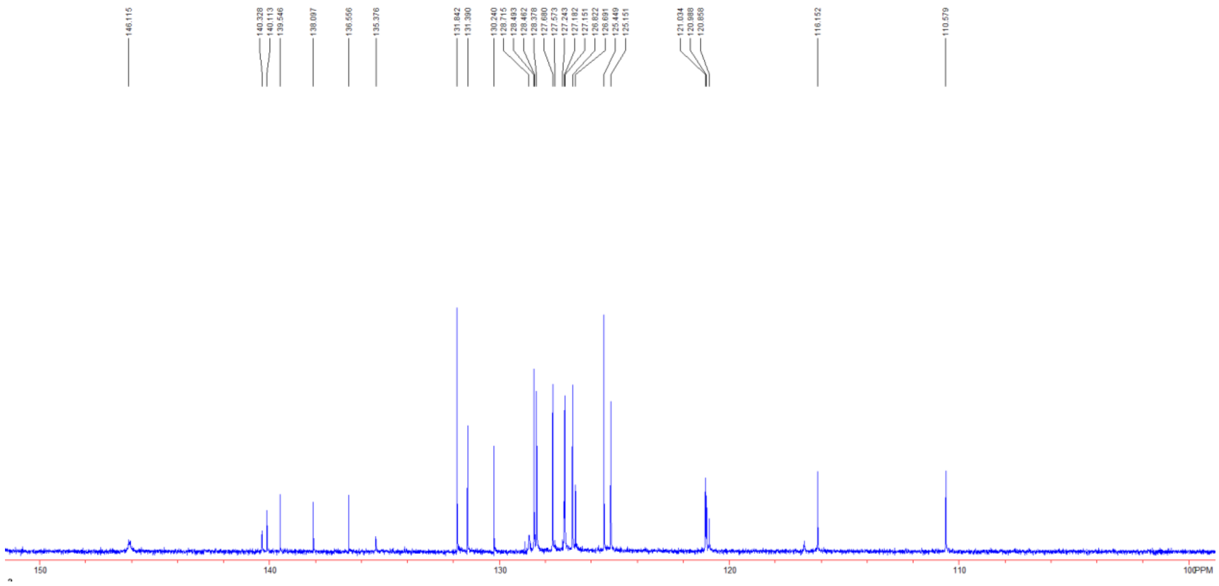

Figure SX22. ^{13}C NMR spectrum of **2a** in $CDCl_3$.

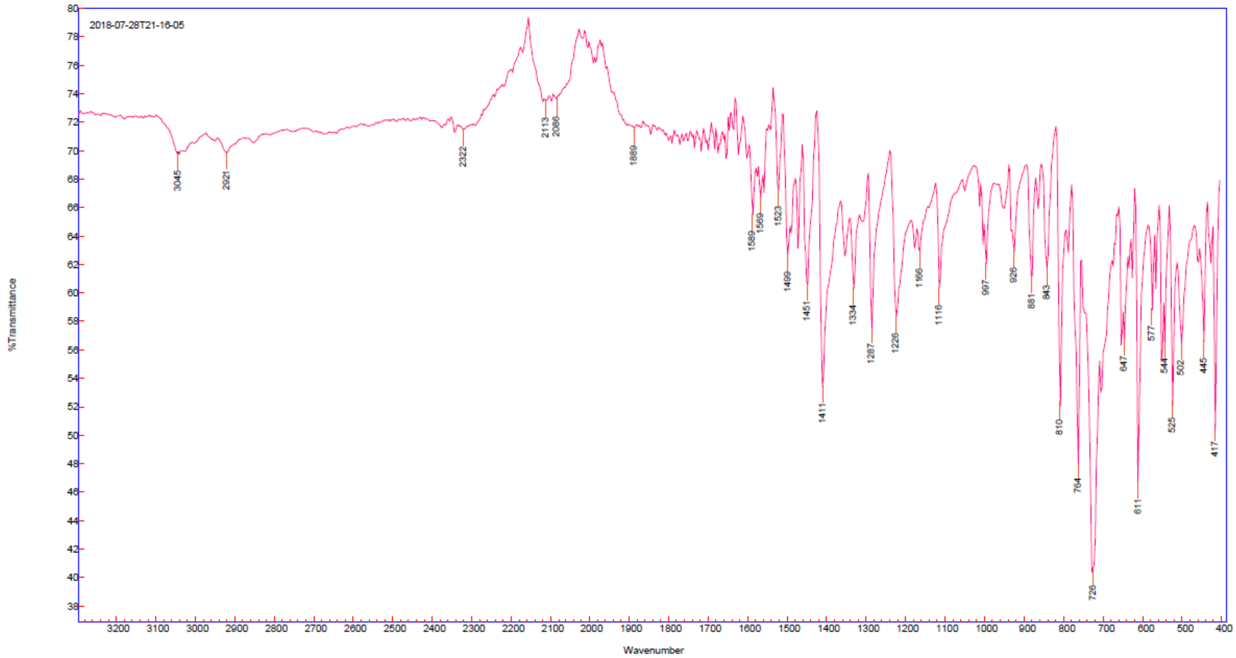

Figure SX23. FTIR spectrum of **2a** (measured by ATR technique).

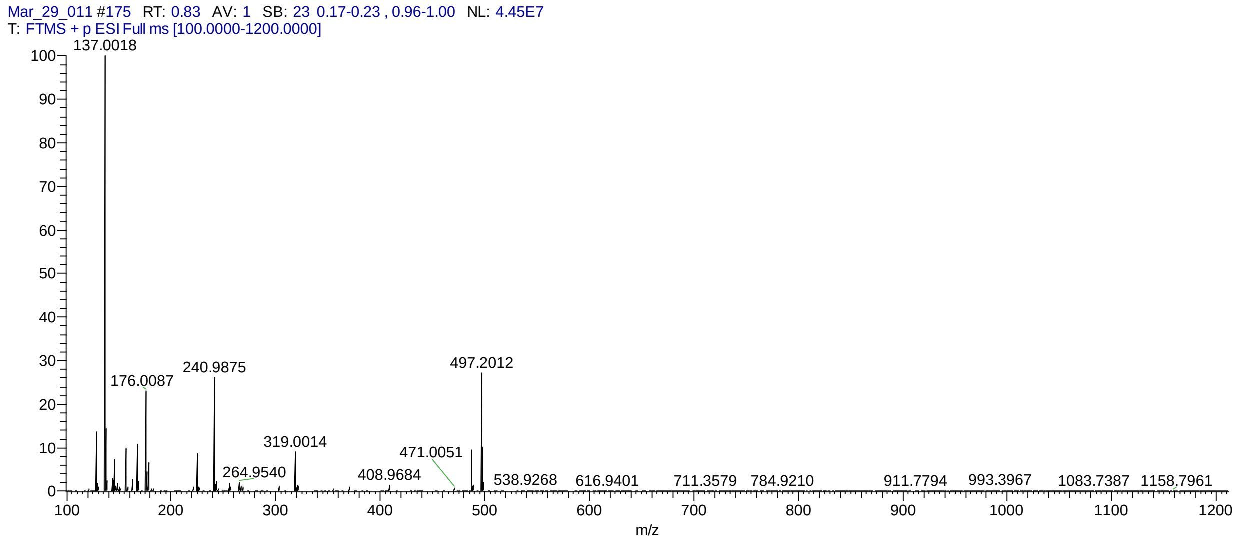

Figure SX24. Mass spectrum of **2a** in MeOH (HRMS, Thermo Scientific Orbitrap Fusion, Positive ionisation mode, Full Scan).

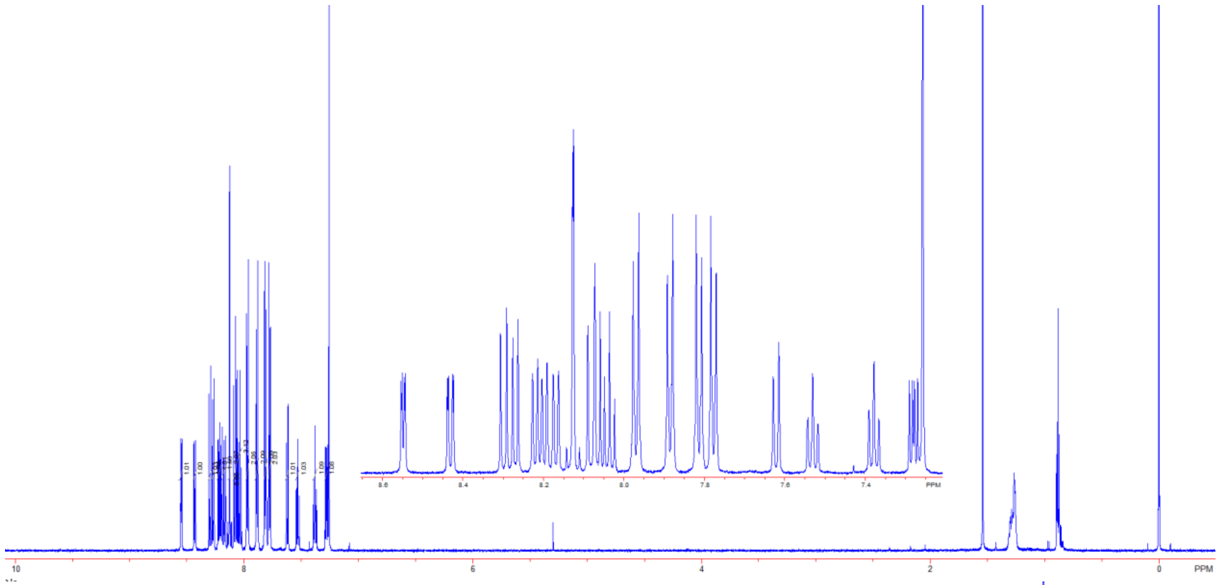

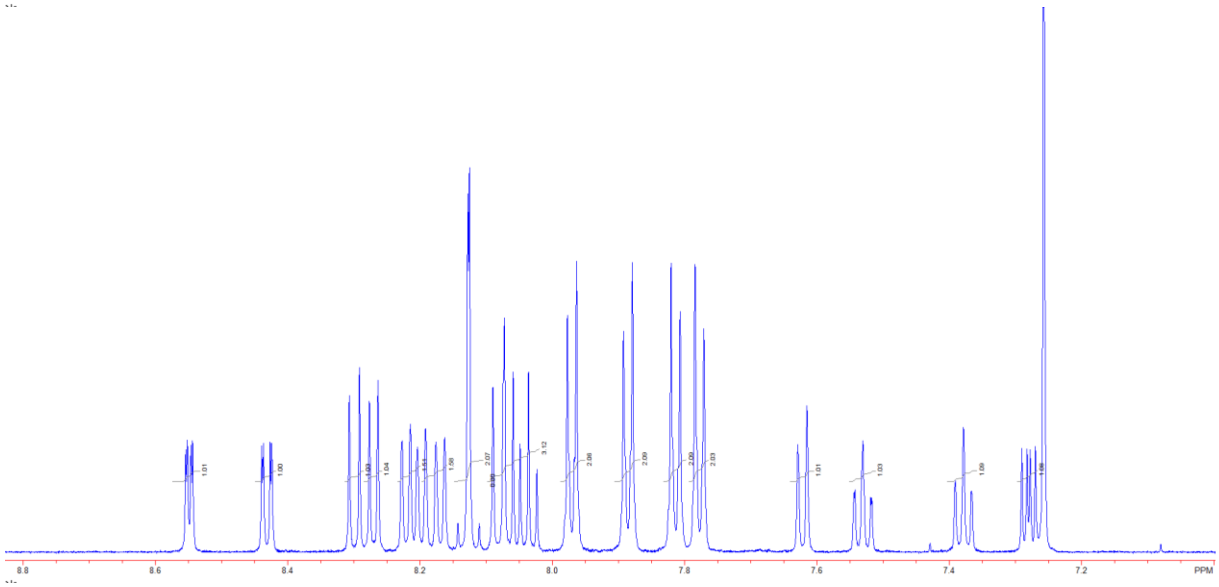

Figure SX25. ^{1}H NMR spectrum of **2b** in $CDCl_3$.

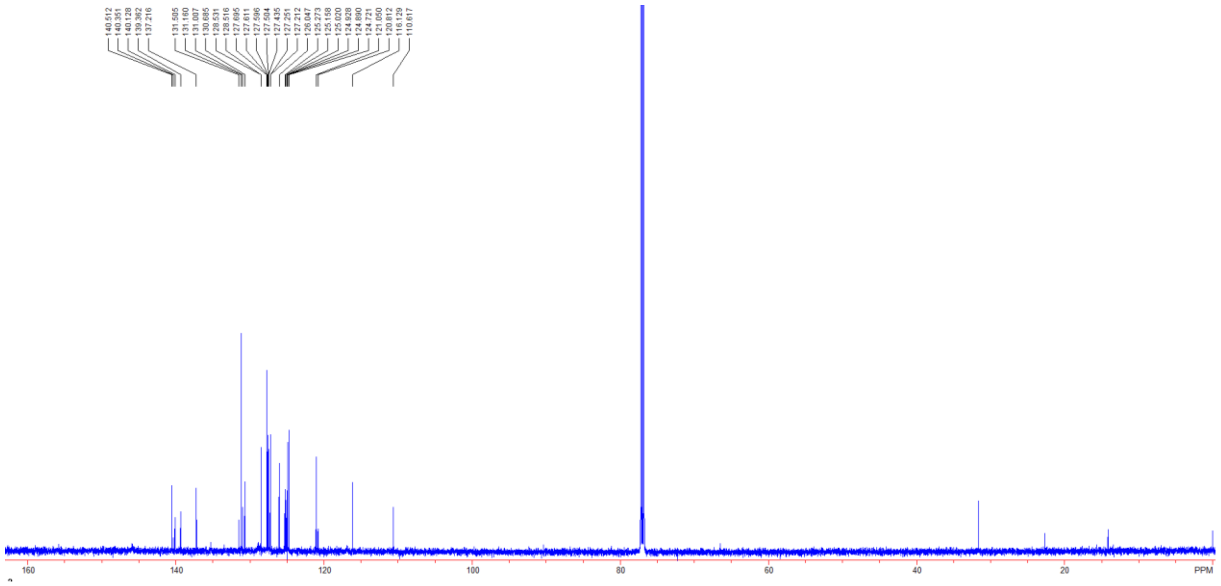

Figure SX26. ^{13}C NMR spectrum of **2b** in $CDCl_3$.

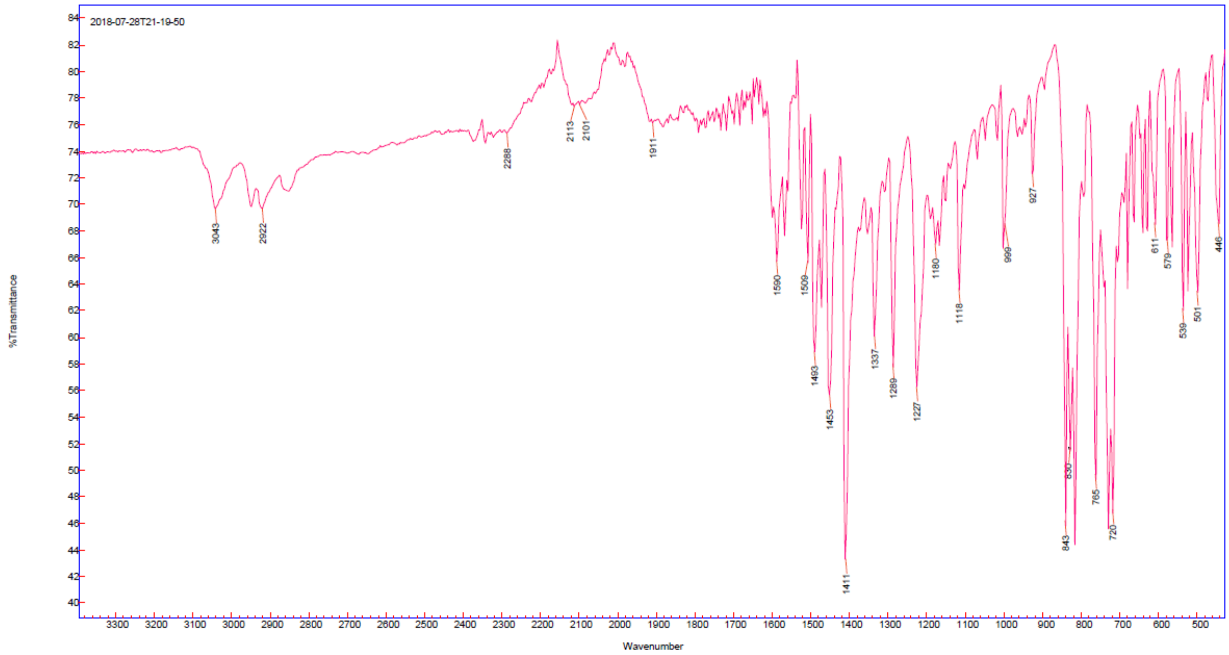

Figure SX27. FTIR spectrum of **2b** (measured by ATR technique).

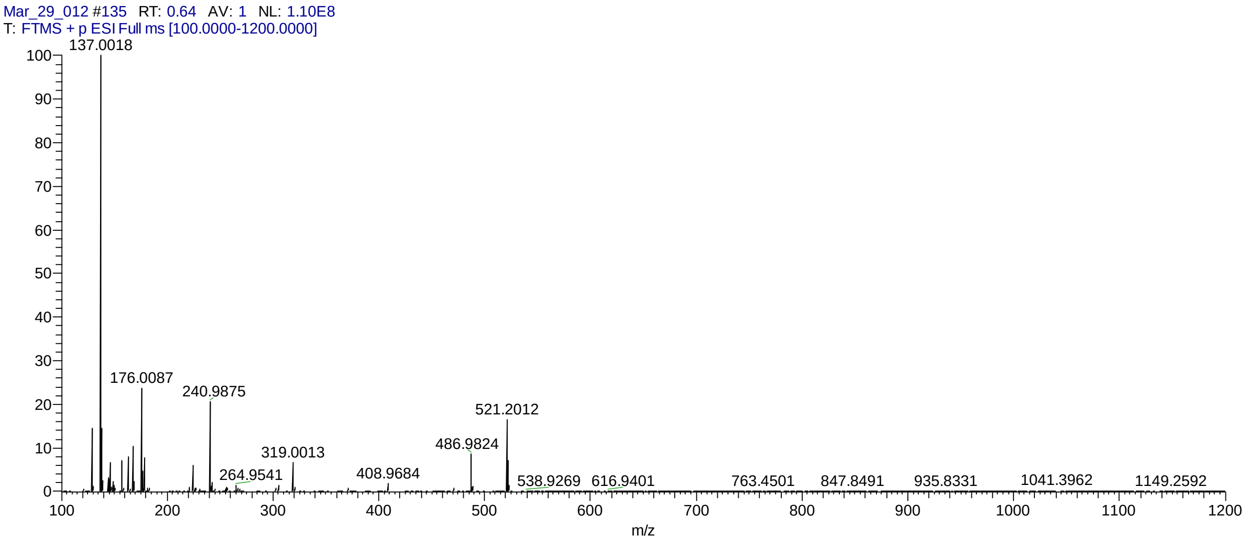

Figure SX28. Mass spectrum of **2b** in MeOH (HRMS, Thermo Scientific Orbitrap Fusion, Positive ionisation mode, Full Scan).

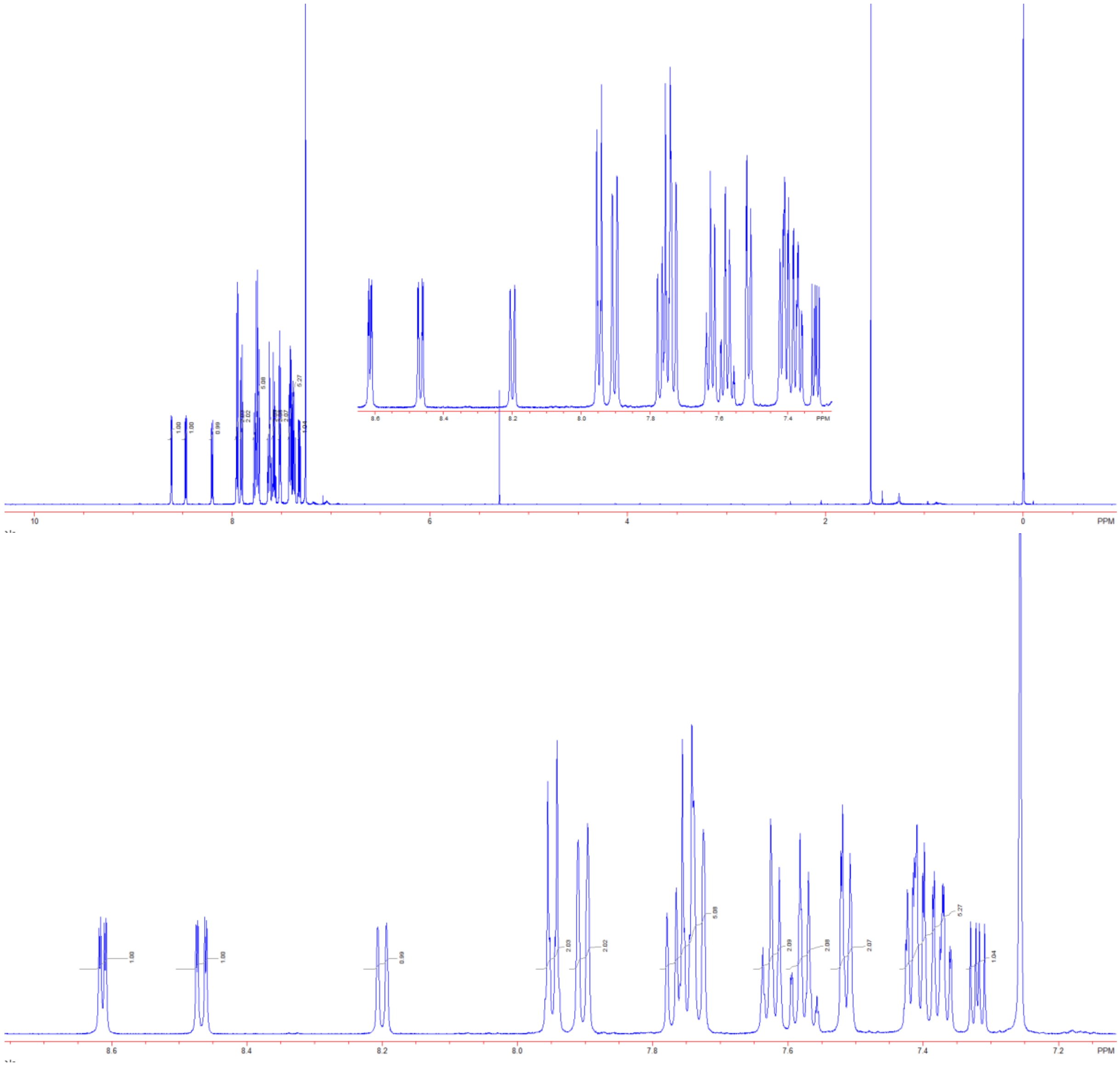

Figure SX29. ^{1}H NMR spectrum of **2c** in $CDCl_3$.

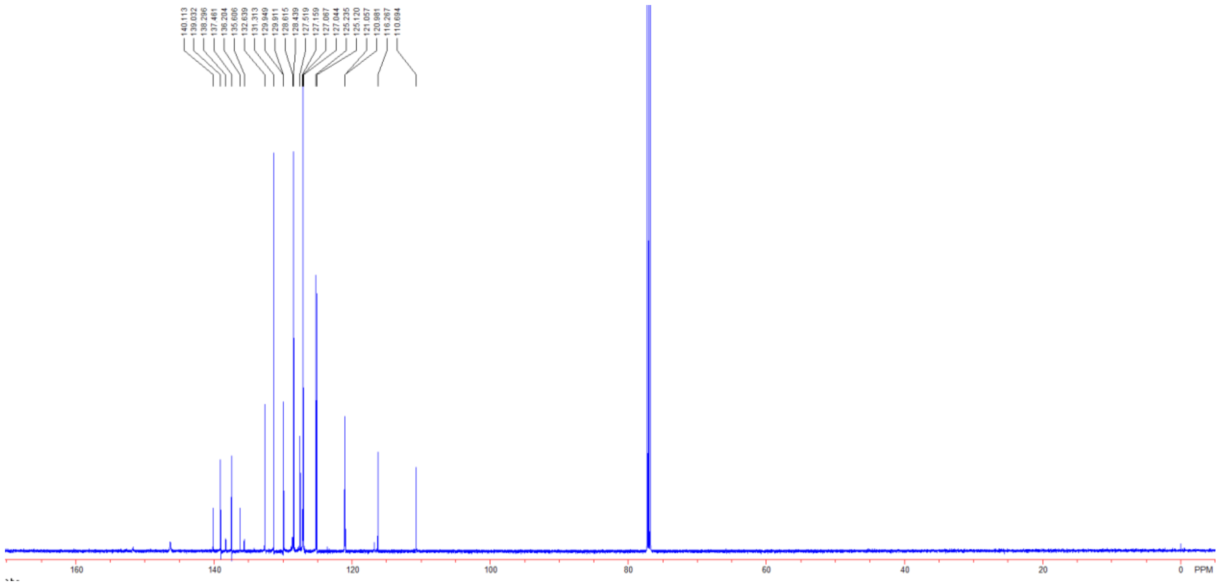

Figure SX30. ^{13}C NMR spectrum of **2c** in $CDCl_3$.

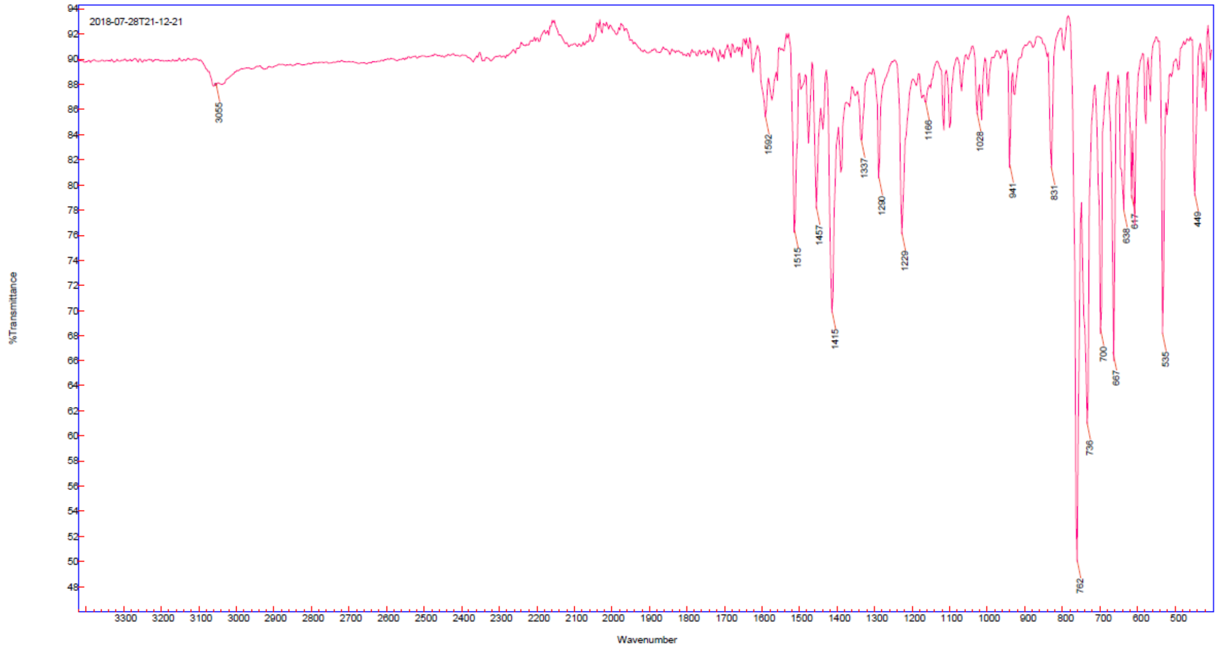

Figure SX31. FTIR spectrum of **2c** (measured by ATR technique).

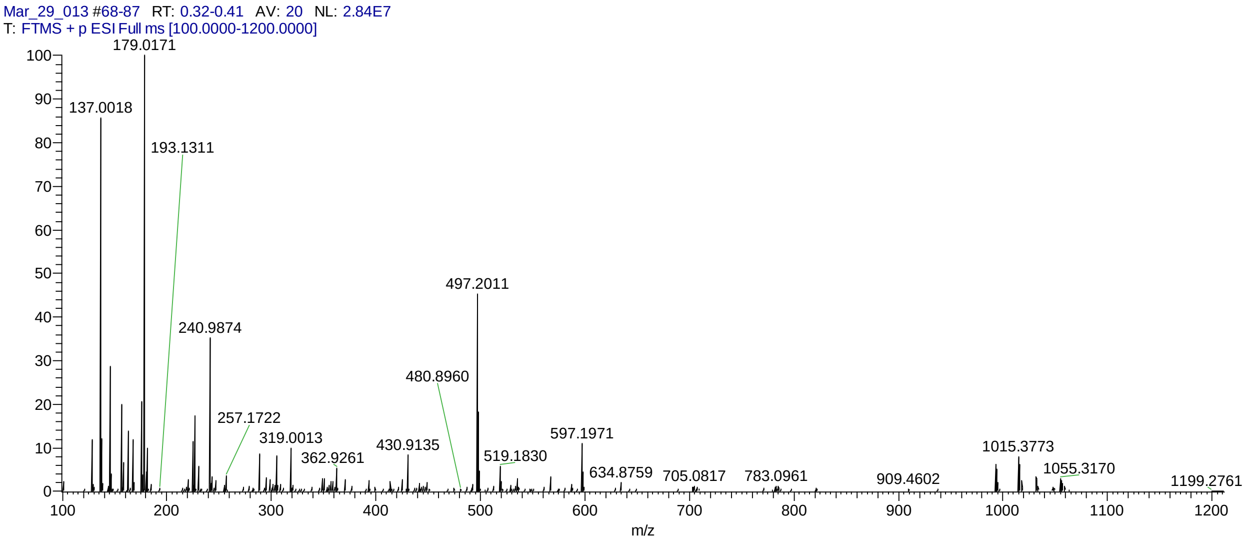

Figure SX32. Mass spectrum of **2c** in MeOH (HRMS, Thermo Scientific Orbitrap Fusion, Positive ionisation mode, Full Scan).

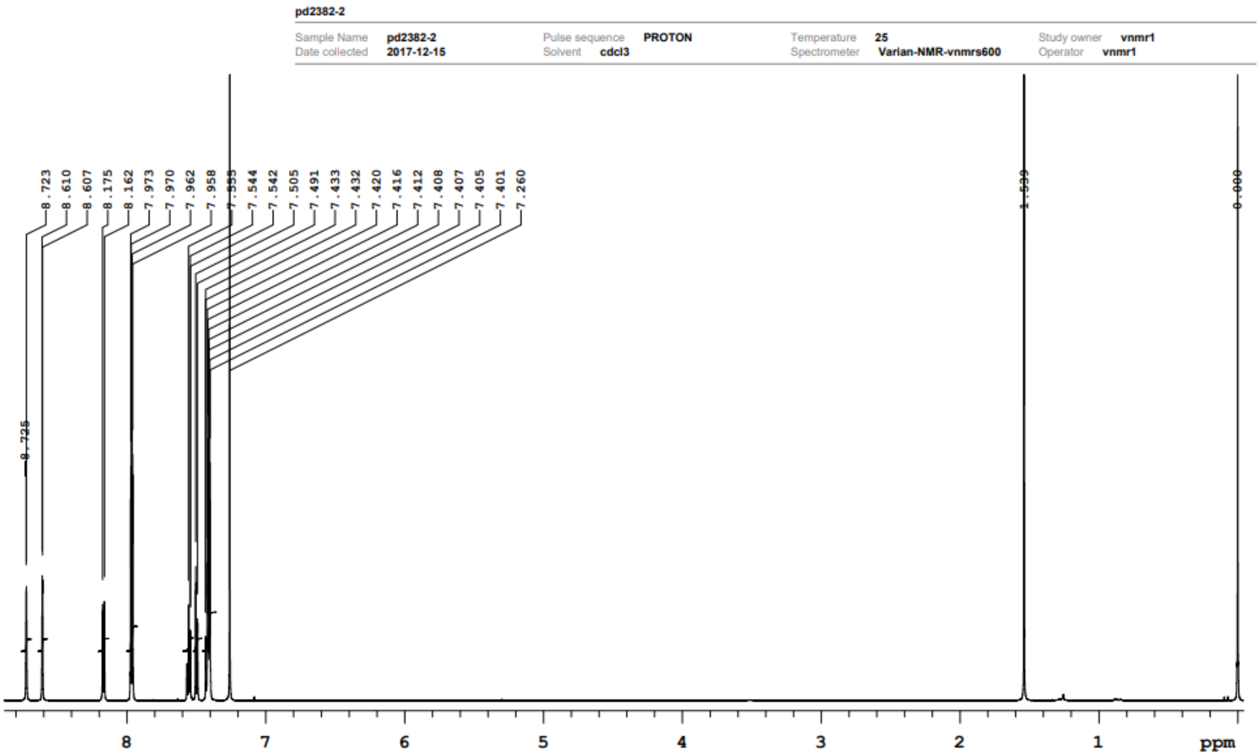

Figure SX33. ^{1}H NMR spectrum of intermediate C.

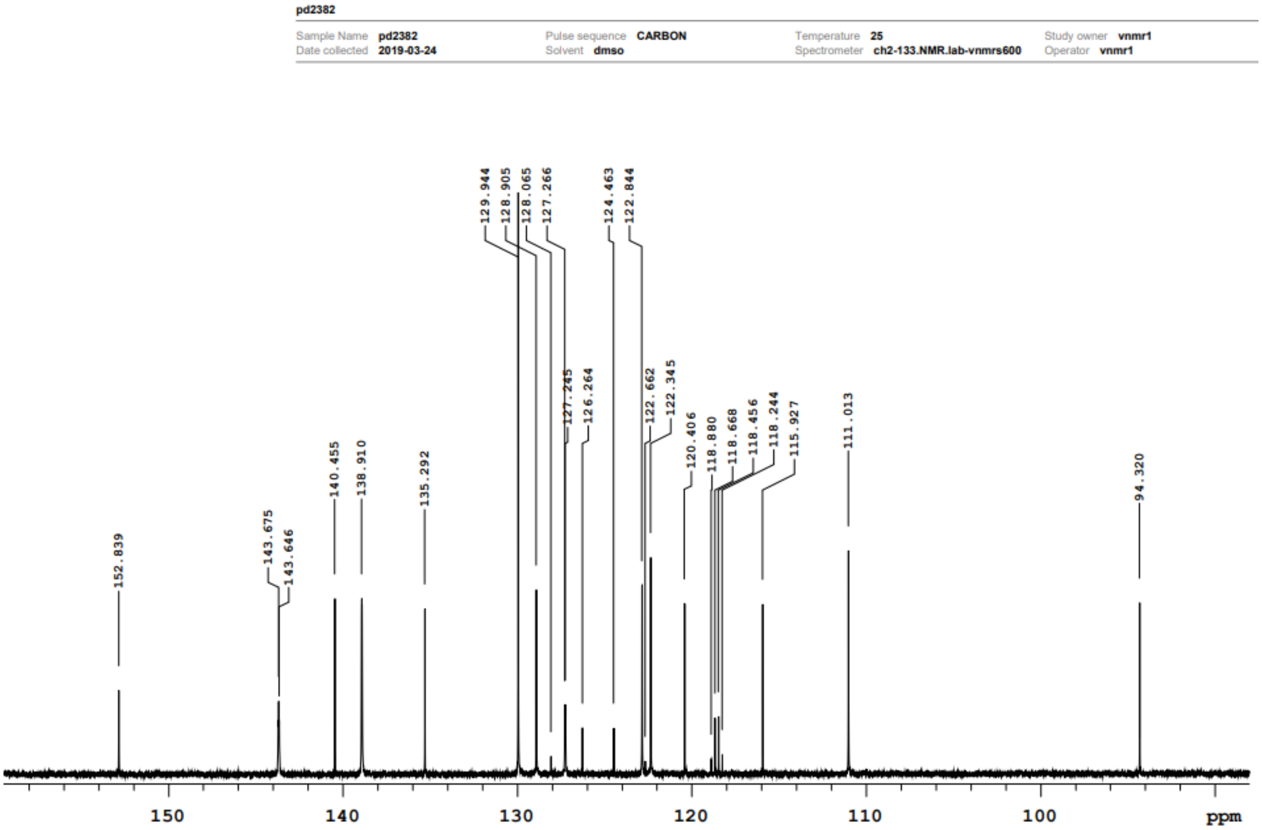

Figure SX34. ^{13}C NMR spectrum of intermediate C.

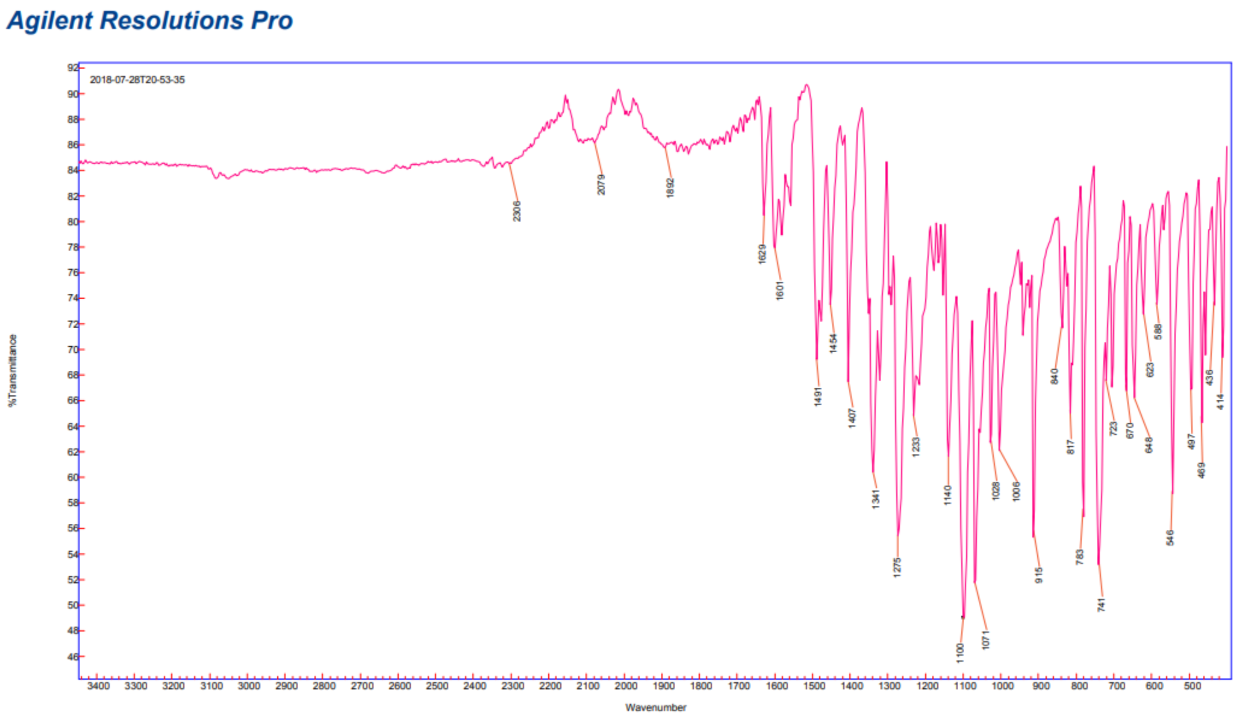

Figure SX35. FTIR spectrum of intermediate C (measured by ATR technique).

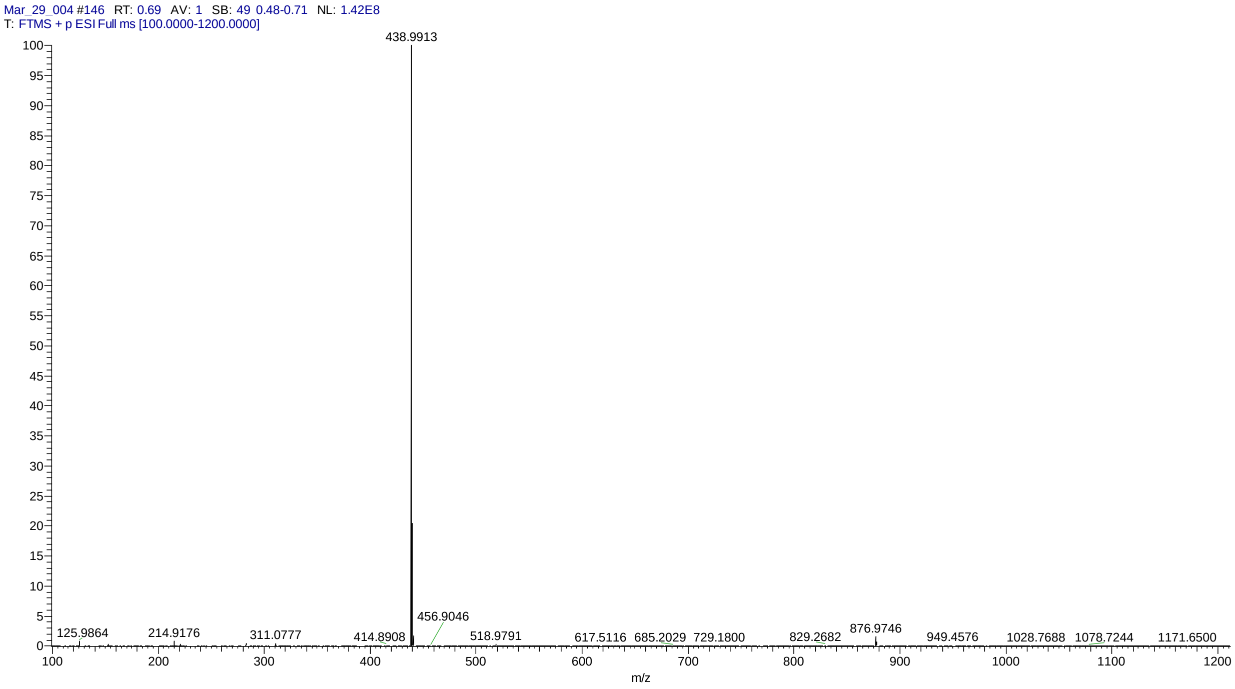

Figure SX36. Mass spectrum of intermediate C in MeOH (HRMS, Thermo Scientific Orbitrap Fusion, Positive ionisation mode, Full Scan).

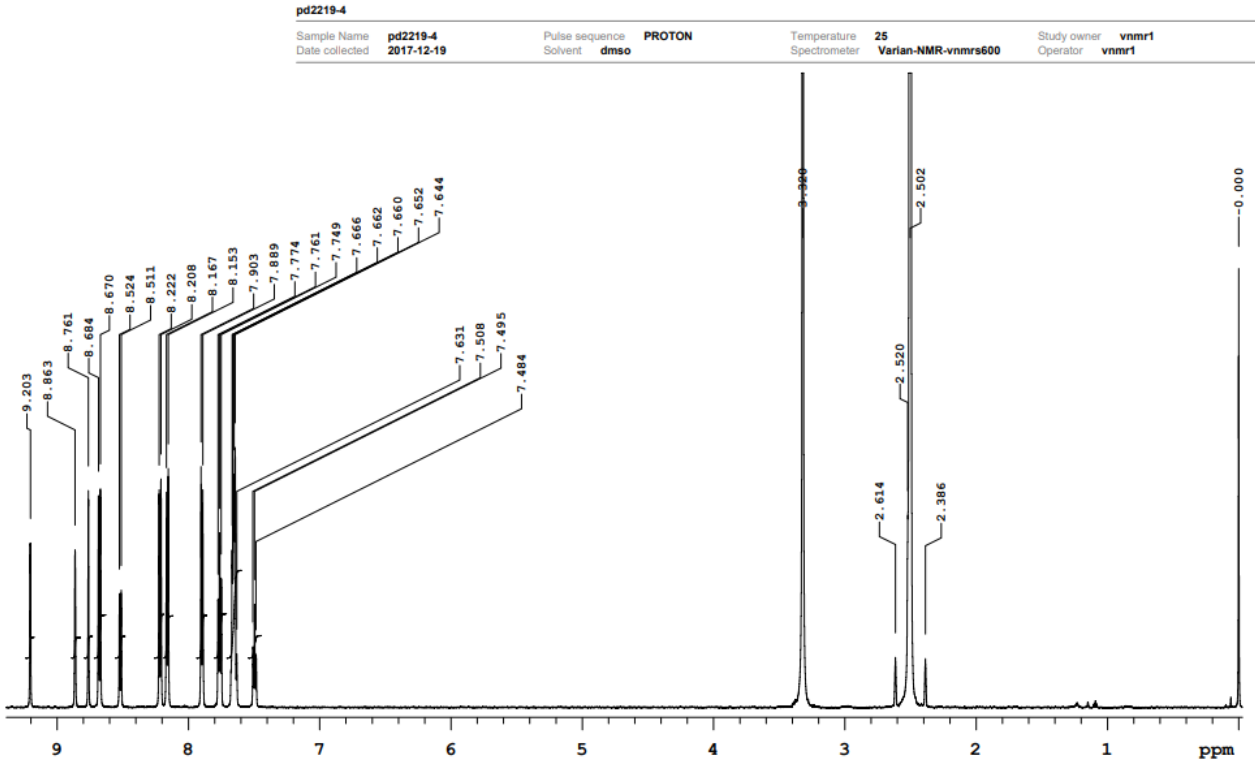

Figure SX37. ^{1}H NMR spectrum of **2d**.

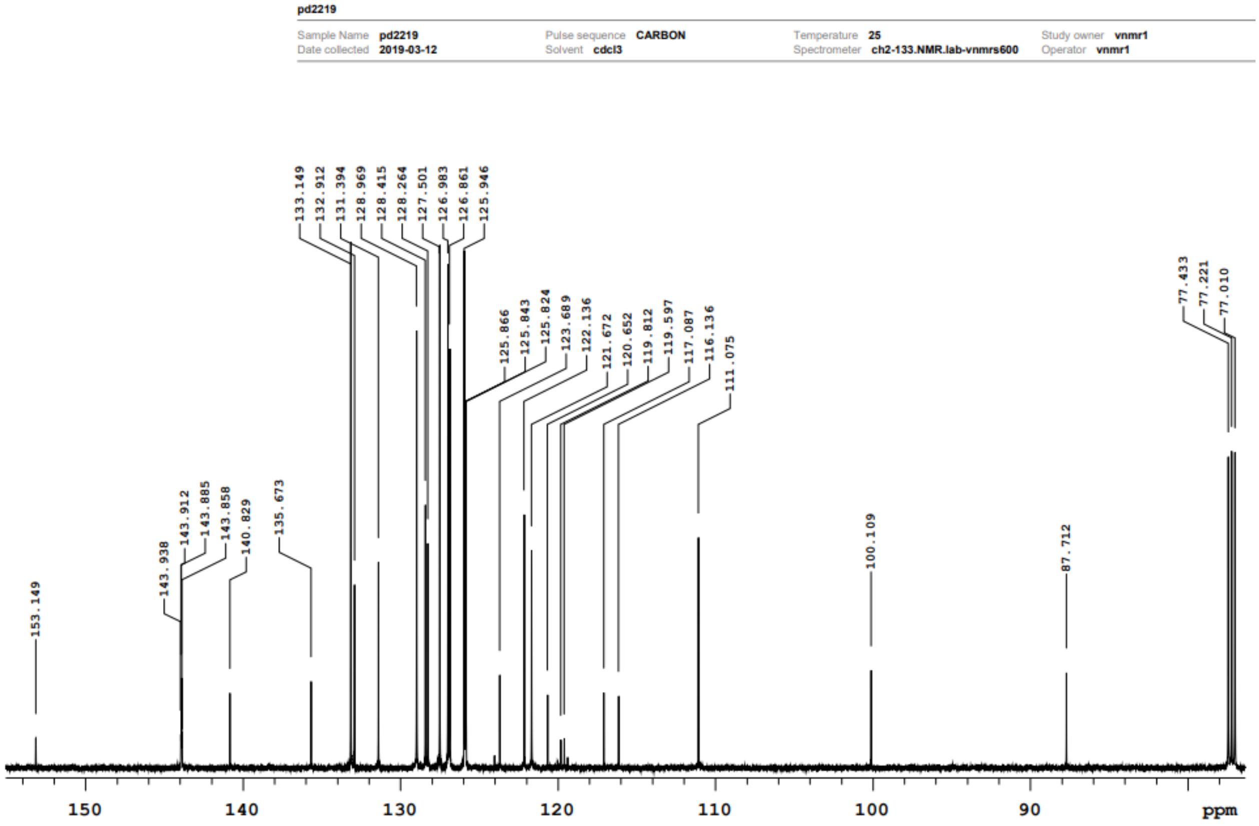

Figure SX38. ^{13}C NMR spectrum of **2d**.

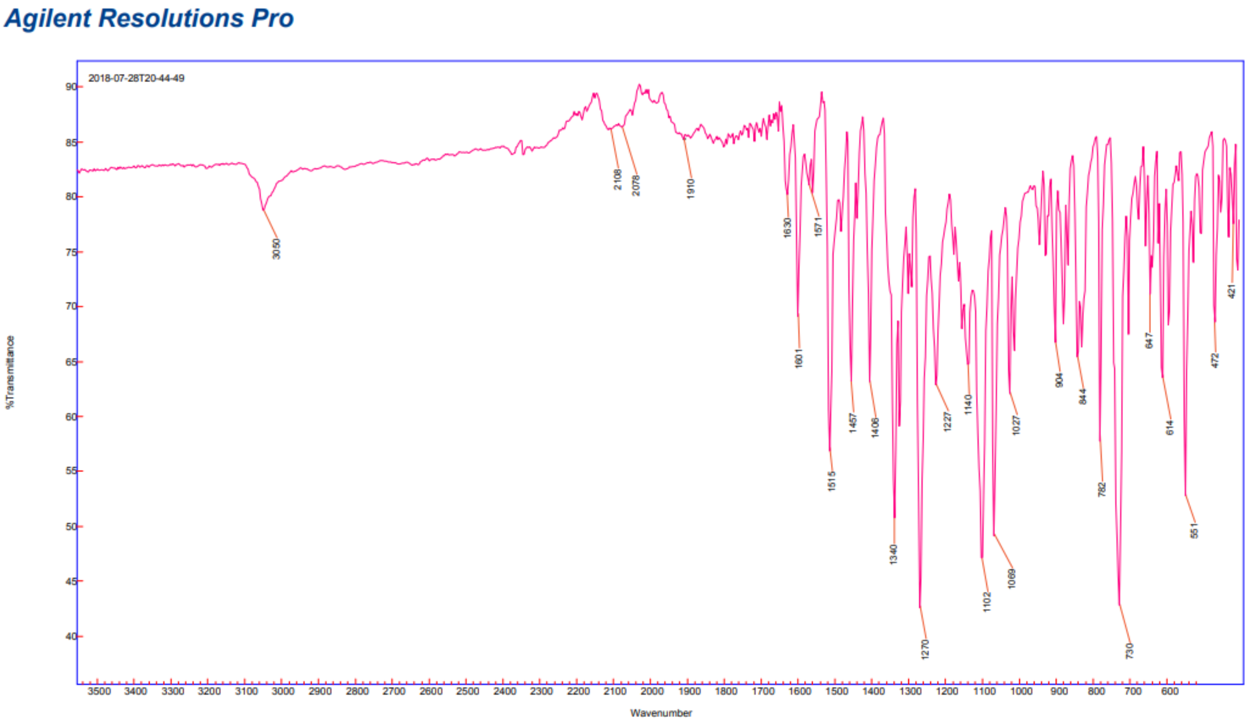

Figure SX39. FTIR spectrum of **2d** (measured by ATR technique).

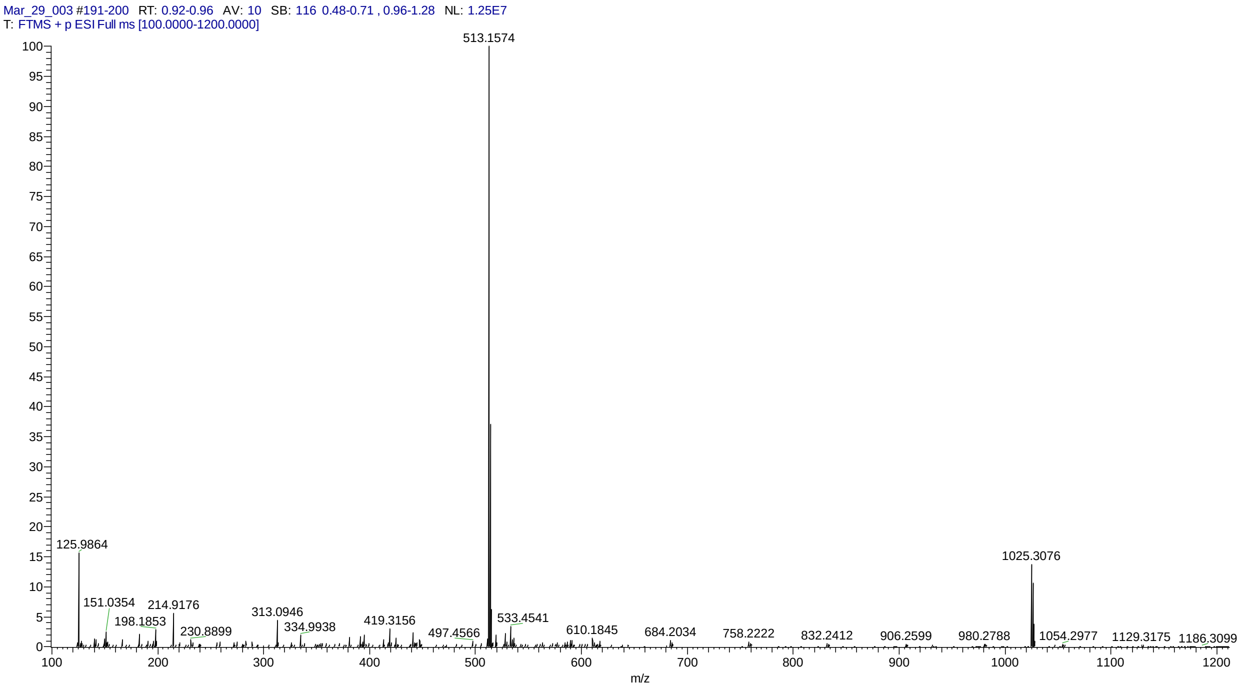

Figure SX40. Mass spectrum of **2d** in MeOH (HRMS, Thermo Scientific Orbitrap Fusion, Positive ionisation mode, Full Scan).

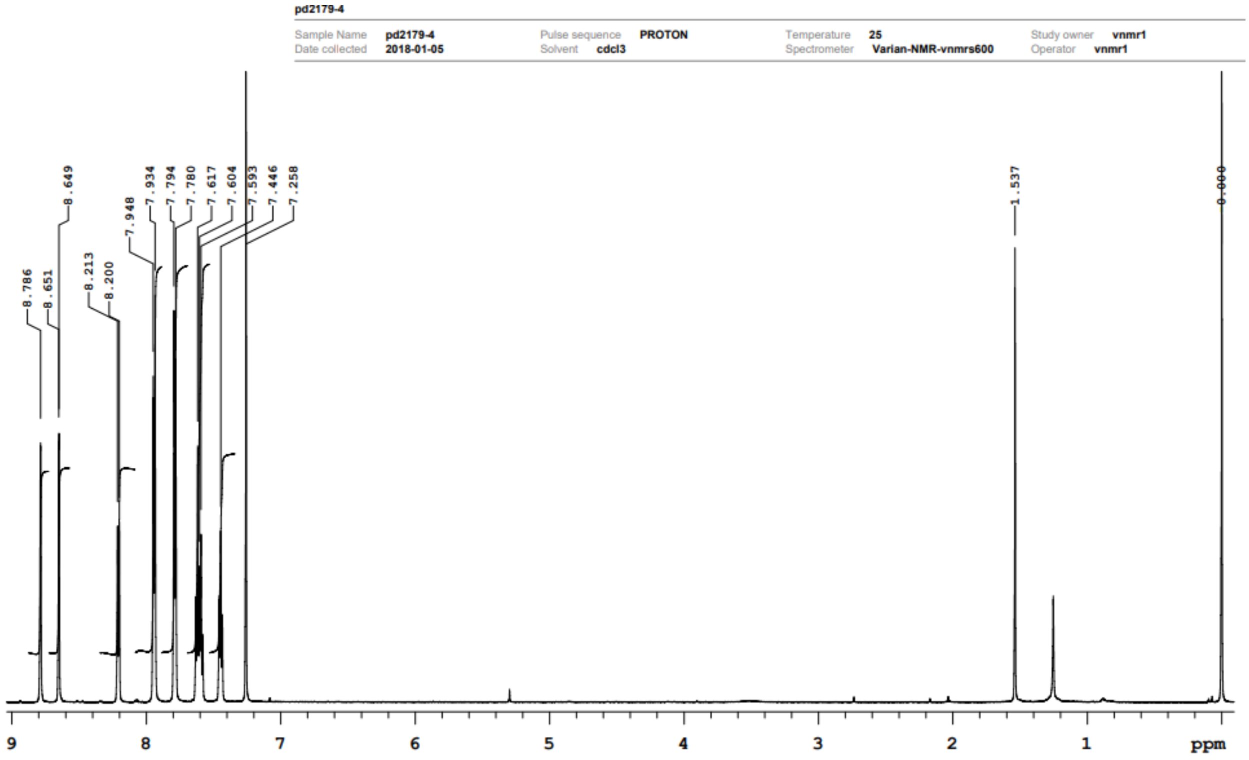

Figure SX41. ^{1}H NMR spectrum of **2e**.

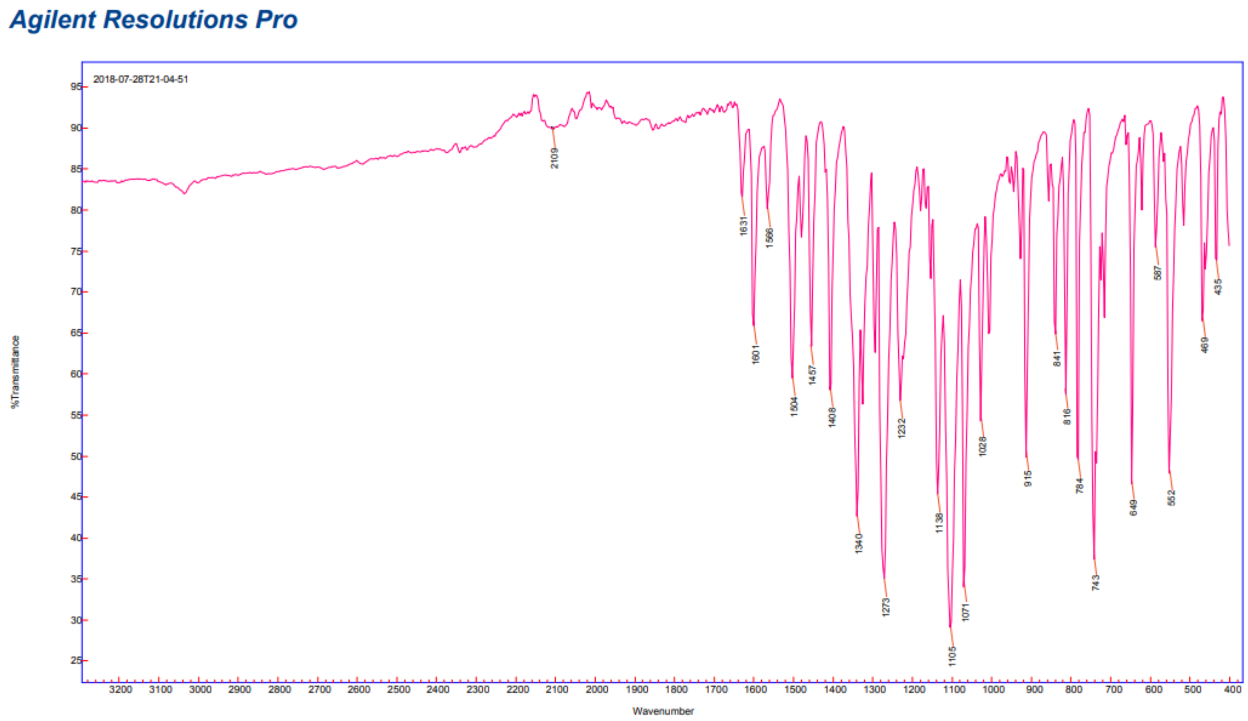

Figure SX42. FTIR spectrum of **2e** (measured by ATR technique).

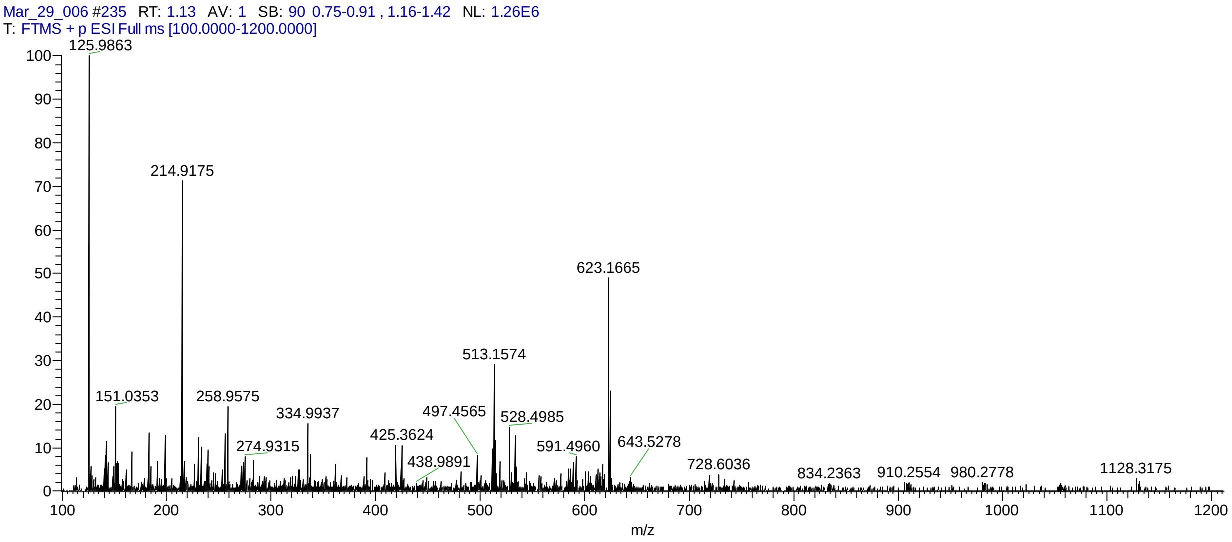

Figure SX43. Mass spectrum of **2e** in MeOH (HRMS, Thermo Scientific Orbitrap Fusion, Positive ionisation mode, Full Scan).
